# Online Coupling High‐Temperature Electrolysis with Carbonylation Reactions: A Powerful Method for Continuous Carbon Dioxide Utilization

**DOI:** 10.1002/anie.202420578

**Published:** 2025-04-07

**Authors:** Kristof Stagel, Kirsten Rath, Prasad M. Kathe, Michael Schnürch, Tobias M. Huber, Alexander K. Opitz, Katharina Bica‐Schröder

**Affiliations:** ^1^ Institute of Applied Synthetic Chemistry TU Wien Getreidemarkt 9/163 Wien 1060 Austria; ^2^ Institute of Chemical Technologies and Analytics TU Wien Getreidemarkt 9/164 Wien 1060 Austria

**Keywords:** Carbonylation, Catalysis, Electrochemistry, Flow chemistry, Sustainable chemistry

## Abstract

Despite being widely available, the broad utilization of CO_2_ for chemical production remains in its infancy. The difficulty of using CO_2_ as an important resource for the chemical industry lies in the high stability of the molecule and its associated inertia. In this work, we demonstrate how to overcome these limitations by utilizing a solid oxide electrolysis cell (SOEC) with an optimized cathode to produce dry CO through the high‐temperature electrolysis of CO_2_. We further integrate this process with a synthesis setup in a continuous‐flow coil reactor to boost reactivity in various carbonylation processes, including in amino‐, alkoxy‐, and phenoxycarbonylation, carbonylative Sonogashira couplings, or the synthesis of redox‐active esters. Ultimately, our approach offers a platform strategy for the rapid, scalable, and continuous production of carbonyl compounds directly from CO_2_ while eliminating the requirement for storing large CO quantities.

## Graphical abstract

A continuous process for carbonylation reactions based on the online coupling of dry, high‐temperature CO_2_ electrolysis with a coil reactor has been reported. The strategy relies on producing CO/CO_2_ mixtures onsite, without H_2_ or H_2_O entering the product gas, thus providing a safe and fast concept for the synthesis of several carbonyl compounds. The method demonstrated widespread applicability in amino‐, alkoxy‐, and phenoxycarbonylation, in carbonylative Sonogashira couplings, and in the synthesis of redox‐active esters. The corresponding products were isolated with excellent yields in just 40 min.

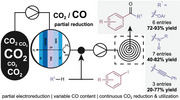

## Introduction

Since its first industrial applications,^[^ ^1^, ^2^ ^]^ carbon monoxide (CO) as a C1 building block has found widespread utilization in transition metal‐catalyzed carbonylation reactions on both industrial and laboratory scales.^[^ ^3^, ^4^, ^5^, ^6^, ^7^ ^]^ However, its intrinsic toxicity renders the use of carbon monoxide a severe risk, especially when it comes to an industrial application, where large amounts of this gas have to be stored and ultimately transported to the place of its use.^[^ ^8^, ^9^ ^]^ Furthermore, its low solubility^[^ ^10^, ^11^, ^12^ ^]^ in many solvents hampers reaction kinetics. To cope with solubility issues, most of the reactions can be carried out at elevated pressure, which conversely introduces complexities in the required instrumentation and exacerbates safety concerns.

The replacement of CO with less toxic and easier‐to‐manage alternatives is thus highly desirable, and several CO sources, including formates^[^ ^13^, ^14^ ^]^ or even stoichiometric amounts of metal carbonyls,^[^ ^15^ ^]^ have been used. These costly options suffer from low atom efficiency, making them less attractive from an application point of view. Most recently, considerable attention has been drawn to the utilization of carbon dioxide (CO_2_) as a non‐toxic, abundant, and low‐cost replacement of CO in the formation of valuable products. However, while the reactivity of CO_2_ is generally modest, its conversion to the desired precursor often requires an additional reaction step, thereby increasing the complexity of the synthesis. A viable compromise would be to rely on CO_2_ as a storage and transport form but to produce CO locally at the reaction site in exactly the required quantity so that it can be immediately converted again and thus consumed.

Existing approaches applying CO_2_ in carbonylation chemistry still face considerable limitations. The most common approaches for CO_2_‐based carbonylation processes are based on the reverse water gas shift reaction (rWGSR; CO_2_ + H_2_ ⇄ CO + H_2_O).^[^ ^16^, ^17^, ^18^, ^19^ ^]^ However, the inherent presence of H_2_O and H_2_ contained in the gas stream limits the broad applicability to a few reactions.^[^ ^20^ ^]^ Especially when moisture‐sensitive catalysts or substrates containing reducible functionalities are employed, the feed gas needs to be free of H_2_O and H_2_. This requirement underscores the significance of developing dry CO_2_ reduction processes to enable broader applicability in carbonylation chemistry.

All current methods that avoid the challenges linked to using H_2_ as a reducing agent for CO_2_ continue to encounter significant limitations. He et al. reported a photoreduction strategy for utilizing CO_2_ in carbonylation reactions.^[^ ^21^ ^]^ A Re‐based photocatalyst system was used for the in situ reduction of CO_2_ to CO, which was further employed in aminocarbonylations, alkoxycarbonylations, and carbonylative Suzuki couplings. However, the reduction of CO_2_ proved to be kinetically slow, and relatively long reaction times were required to form the corresponding products. Furthermore, additional photosensitizers are necessary for efficient two‐electron photoreduction, and catalyst aging, and thus limited long‐term stability prevents the application in continuous carbonylation processes.^[^ ^22^, ^23^, ^24^ ^]^

An alternative approach is based on the electrochemical reduction of CO_2_ by Jensen and co‐workers with an iron porphyrin catalyst.^[^ ^25^ ^]^ While a broad range of carbonylation products, including the pharmaceutically active derivatives butoxycaine, olaparib, and moclobemide, could be formed in a specifically designed two‐chamber reactor, the system suffered from slow CO release due to low current densities and thus long reaction times. Such slow CO_2_ electrolysis kinetics are a common issue in the case of the respective electrochemical cells operating at ambient temperatures.^[^ ^26^ ^]^ Moreover, CO_2_ reduction in electrolysis cells with aqueous electrolyte still cannot avoid the presence of H_2_O and H_2_ in the product gas stream–the first at partial pressure levels due to its vapor pressure, the latter as an undesired by‐product originating from water electrolysis. A novel approach has been developed by Gaudeau and co‐workers for the fast aminocarbonylation of *p*‐iodoanisole with hexylamine.^[^ ^20^ ^]^ The implemented technology relies on decomposing CO_2_ into CO by plasma and provides the corresponding amide with excellent yield (95%) in just 40 s. However, this unique technology for carbonylation, providing rapid access to amides, was limited to aminocarbonylation. Additionally, all CO_2_‐based carbonylative strategies reported in the literature are carried out in a batch‐wise fashion and are hampered by long reaction times. Most recently, a tandem electrocatalytic‐thermocatalysis system was reported for CO_2_ reduction, followed by oxidative carbonylation for the synthesis of ureas.^[^ ^27^, ^28^ ^]^ The group employed copper and cobalt catalysts for the carbonylative coupling of primary and secondary amines to form biologically active asymmetric ureas. The corresponding products were obtained with excellent yields.

Here, we showcase how to surmount these limitations and utilize a solid oxide electrolysis cell (SOEC) with an optimized, metal‐free CeO_2_‐based cathode to generate CO through high‐temperature electrolysis of dry CO_2_ and couple it online with the synthesis set‐up for various carbonylation reactions, ultimately presenting a platform strategy for the fast, scalable, and safe formation of carbonyl components directly from CO_2_.

## Results and Discussion

### Electrode Development for Dry CO_2_ Electrolysis

High‐temperature electrolysis of CO_2_ in SOECs is currently the most efficient method available to reduce CO_2_ to CO,^[^ ^29^, ^30^ ^]^ which also meets the requirement of carbonylation reactions, i.e., a gas stream leaving the cell that only contains the necessary components CO and CO_2_. In an SOEC, the electrochemical reduction of CO_2_ proceeds at the cell's cathode with the consumption of two electrons via the reaction in Equation 1.

(1) $${CO}_{2}+2e^{-}\to \mathrm{CO}+O^{2-}$$

The oxide ions are taken up by the cathode, transported through the oxide ion conducting electrolyte, and released at the anode as gaseous O_2_ while leaving their electrons behind (Equation 2), thus closing the electric circuit.

(2) $$O^{2-}\to \frac{1}{2}O_{2}+2e^{-},$$

Even though dry CO_2_ reduction in SOECs is in principle possible, it poses a major challenge for current state‐of‐the‐art electrode materials. Common SOEC cathodes are typically composites of ceramic oxide ion conductors and Ni metal,^[^ ^31^ ^]^ which catalyzes the formation of carbon deposits by the Boudouard reaction if the CO content is too high. Especially at higher overpotentials, this can lead to degradation and, in the worst case, to the destruction of the electrode since very high CO activities can be effective locally in the porous structure of the electrode close to the electrolyte.

A common coping strategy is to add steam to the feed gas, thus operating the SOEC in so‐called co‐electrolysis mode.^[^ ^32^ ^]^ However, as H_2_O and H_2_ are therefore contained in the gas stream, this option is not suitable for the application aimed at this work. Our novel approach avoids this problem by using a catalytically optimized electrode material, specifically Gd‐doped CeO_2_ (GDC). This decision was based on the following unique properties of GDC, which have been extensively explored on well‐defined model systems: Doping with Gd^3+^ (or equivalently Sm^3+^) generates oxygen vacancies, providing oxide ion conductivity at elevated temperatures.^[^ ^33^ ^]^ Under strongly reducing conditions–as they appear at a SOEC cathode under electrolysis conditions–the material becomes electronically conducting due to a valence change of Ce^4+^ to Ce^3+^, which represents a polaronic n‐type charge carrier.^[^ ^34^, ^35^, ^36^ ^]^ At the GDC surface, the Ce^3+^ concentration is even enhanced, which causes the material's high catalytic activity for redox reactions,^[^ ^37^, ^38^, ^39^, ^40^ ^]^ enabling CeO_2_‐based electrodes to catalyze CO oxidation and CO_2_ reduction.^[^ ^40^, ^41^, ^42^ ^]^ Moreover, at the same time, CeO_2_‐based cathodes offer the remarkable feature that carbon deposition on their surfaces is strongly kinetically inhibited, which makes this material an excellent candidate for the dry, high‐temperature splitting of CO_2_ in an SOEC.^[^ ^43^, ^44^, ^45^ ^]^ To demonstrate that the GDC used here (with composition Ce_0.9_Gd_0.1_O_1.95_) offers the same surface chemistry as the CeO_2_‐based materials investigated in the literature, we spectroscopically examined a GDC thin‐film electrode in a CO/CO_2_ atmosphere under cathodic polarization using near ambient pressure XPS. The spectra shown in the SI (Figure S3) show that a carbonate‐like intermediate of CO_2_ reduction^[^ ^41^ ^]^ also appears on the GDC used here, and that no graphite‐like carbon can be detected. Thus, it can be safely assumed that our GDC exhibits the same surface catalytic properties as the model‐type CeO_2_‐based SOEC cathodes described in the literature.

As already indicated above, in commercial SOECs, GDC is usually combined with metallic Ni, which provides a certain mechanical stability and electronic conductivity, but catalyzes the formation of carbon deposits during CO_2_ electrolysis. As already shown in earlier work,^[^ ^46^ ^]^ Ni is not needed as an electronic current collector for the special case of GDC cathodes due to its pronounced n‐type polaronic conductivity. Here, we make use of these insights, and the uniqueness of the present work lies in employing a Ni‐free, GDC‐based cathode for dry CO_2_ electrolysis in a SOEC lab prototype, leveraging insights from previous model studies to develop a highly porous, purely ceramic cathode for practical application. Another advantage of our approach–performing electrochemical reduction of dry CO_2_ in a SOEC with a pure GDC cathode–is also that the electrocatalyst for CO_2_ splitting is a ceramic material and not a scarce and noble metal with critical raw material status, as typically found in electrolysis cells with aqueous electrolyte.

**Figure 1:**
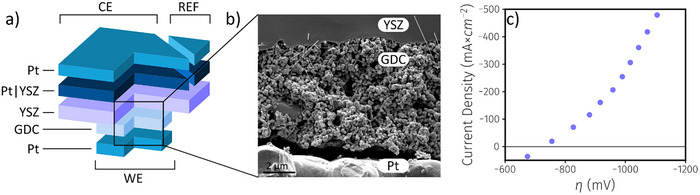
a) Sketch of a model‐type cell on which the kinetics and catalytic properties of the GDC cathode were investigated. The GDC10 electrode is smaller than the counter electrode to leave space for the gasket in the 2‐gas measurement set‐up. b) Post‐mortem SEM cross‐section of SOEC cathode region, showing the YSZ single crystal electrolyte, porous single‐phase GDC cathode, and porous Pt current collector. On the porous GDC cathode growth of some carbon nanotubes (i.e., the bright, straight needle‐shaped formations) is visible. In fact, it can be seen that the formation of the carbon nanotubes seems only to occur close to the YSZ electrolyte, which is in accordance with the fact that the electrochemical polarisation of GDC is highest at the interface to the electrolyte.^[^ ^47^, ^48^ ^]^ c) Current density versus overpotential curve of the WE with a gas flow of 30 mL min^−1^ of 1% CO in CO_2_ (η is given vs. 10 mbar O_2_ at REF).

To demonstrate the performance as well as the coking resilience of our pure GDC cathode without the risk of damaging the in‐house buildt SOEC lab prototype that was employed in the actual synthesis experiments–e.g., by irreversible processes that can occur when the coking threshold is intentionally exceeded–initial studies were performed on model‐type cells. These model cells consisted of an Y‐doped ZrO_2_ (YSZ) single crystal electrolyte, a Pt/YSZ counter and reference electrode (CE, REF), as well as a pure GDC working electrode (WE). These cells were tested in a 2‐gas set‐up with an atmosphere of 1% O_2_ in N_2_ on the CE and REF side and 1% CO in CO_2_ on the WE side at a temperature of 750 °C. The small amount of CO in the WE feed gas is required to provide thermodynamically well‐defined open circuit conditions. Cathodic bias was then applied to the WE in 200 mV steps. The sample geometry with a reference electrode (Figure 1a) allowed the determination of the overpotential η at the working electrode. Plotting the WE‐overpotential versus the current density results in the I‐η curve displayed in Figure 1c. It shows that applying an overpotential of −1105 mV (vs. 10 mbar O_2_ at REF) resulted in a current density close to −500 mA cm^−^^2^, which is an extraordinarily high value, considering that the cathode feed gas contains no H_2_O.

Subsequently, an attempt was made to provoke carbon deposition by further increasing the cathodic overpotential. Due to the onset of electron conductivity in the electrolyte at these extreme polarizations,^[^ ^49^ ^]^ the overpotential could no longer be reliably determined. What is noteworthy is that, unlike previously reported for model electrodes^[^ ^43^ ^]^ no coking‐induced current drop could be observed for our GDC cathodes even under these harsh conditions, although the cathodic polarization was so strongly reducing that the cell eventually failed due to mechanical stresses in the electrolyte.^[^ ^50^ ^]^ This result already suggests an extraordinarily high resistance against the formation of carbon deposits of the highly porous, metal‐free GDC cathodes. It should also be emphasised at this point that the thickness of the pure GDC electrode was chosen such, that the Pt current collector is sufficiently far away from the electrochemically active region at the GDC/YSZ interface; thus, effects on the electro‐catalytic behavior of GDC or the promotion of carbon growth by Pt can be safely excluded.^[^ ^46^ ^]^

After the electrolysis tests, a cross‐section of the model cell was investigated via scanning electron microscopy (SEM)–see Figure 1b. These investigations confirm that cell failure most likely occurred due to mechanical reasons caused by the chemical expansion of the YSZ electrolyte (with possible contributions also by chemical expansion of the GDC). In contrast to Ni‐containing SOEC cathodes, which would be deactivated by coking already at much lower cathodic polarization,^[^ ^51^ ^]^ the formation of only a few carbon nanotubes was observed in the SEM image of the GDC electrode. These carbon nanotubes also had no significant effect on the electrocatalytic activity of the pure GDC cathode. This confirms that coking was not the limiting factor of our pure, Ni‐free GDC working electrode, thus demonstrating that the peculiarity of our approach is as simple as it is effective: By overcoming the prevailing dogma that SOEC cathodes must contain Ni for sufficient electrochemical activity, we obtain pure GDC cathodes that form only isolated carbon nanotubes even under conditions where Ni‐containing cathodes would already be completely dysfunctional due to massive coking. Ultimately, these initial studies verified that the developed GDC electrode reliably enables CO_2_ electrolyzes for dry CO production, thus clearing the path for the envisioned conversion.

### Characterisation and Operation of the SOEC

**Figure 2:**
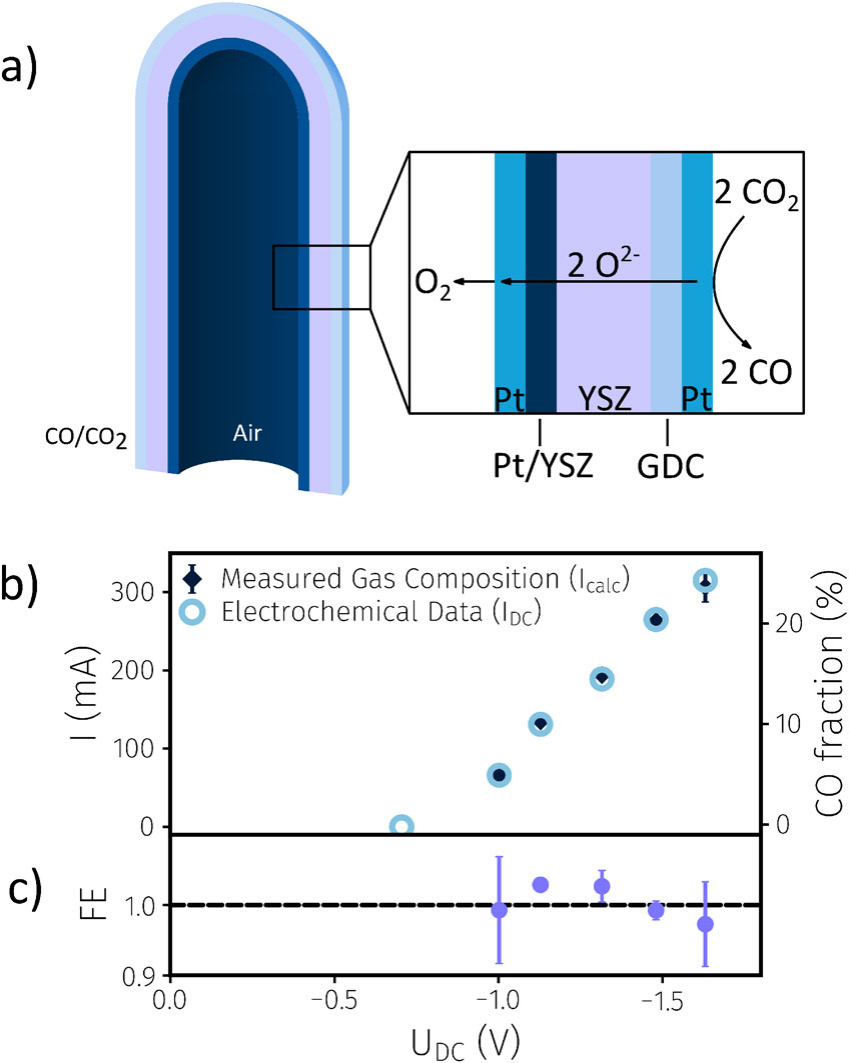
a) Sketched cross‐section of a tubular SOEC consisting of a GDC cathode, Pt/YSZ anode, and YSZ electrolyte, which was used for online coupling CO production via high‐temperature CO_2_ electrolysis with carbonylation reactions. b) Current–voltage curve (I_DC_ vs U_DC_) together with the current I_calc_ obtained from the measurement of the CO concentration in the product gas for a flow of 10 mL CO_2_ min^−1^. The two y‐axes are linked by Faraday's law (Equation S3)–hence, the measured gas composition is proportional to I_calc_. c) Faradaic efficiency FE plotted as a function of the voltage applied to the cell.

Before using the SOEC for CO production and in situ conversion, the electrochemical behavior of our tubular SOEC consisting of GDC cathode, Pt/YSZ anode, and YSZ electrolyte was characterized (Figure 2a). Therefore, the SOEC was heated to 750 °C in a tube furnace, and as feed gas, a mixture of ca. 20 mbar H_2_, 25 mbar H_2_O, and 950 mbar Ar was supplied to the GDC cathode (see SI for details). Under these conditions, an open cell voltage (OCV) of 0.971 V was measured, which is in good agreement with the theoretical voltage of 0.982 V expected from Nernst's equation, indicating that the cell is gas‐tight and both electrodes equilibrate with their gas atmospheres.

After this test, the atmosphere at the cathode was switched to CO_2_, and the faradaic efficiency of the electrolysis was determined at two different gas fluxes of 10 mL and 100 mL CO_2_ min^−1^. This was done by stepwise increasing the applied voltage to the SOEC and measuring the gas composition of the product gas. (Please note that, in contrast to the model samples, the SOEC lab prototype does not have a reference electrode in order to keep its design and manufacturing simpler–therefore, only the total cell voltage U_DC_ can be given in this case.) The comparison of the recorded *I–V* curve and the resulting CO concentration for feeding 10 mL CO_2_ min^−1^ to the SOEC is depicted in Figure 2b. As this figure shows, the current that can be calculated via Faraday's law from the feed gas flux and the measured CO concentration in the product gas nicely follows the electrical current passing through the cell (I_DC_). From the ratio of both the calculated and the measured current, the faradaic efficiency FE can be obtained (see Equation S3; Figure 2c), which is a measure of the electrode's CO selectivity. The average of all FE values measured at a gas flux of 10 mL min^−1^ amounts to 1.019 ± 0.039, thus indicating that the measured FE is statistically indistinguishable from 1 (the given uncertainty represents a 99.5% confidence interval). The same type of experiment was also repeated for a higher feed gas flux of 100 mL CO_2_ min^−1^ (Figure S5). Under these conditions, also an excellent agreement between product gas composition and electrical current was found. The somewhat smaller error bars in Figure S5 are due to the relatively higher accuracy of the mass flow controller at higher flow rates. Altogether, these results strongly suggest that our pure GDC cathode selectively splits CO_2_ into CO without the formation of elemental carbon (which would lead to CO‐related FE values significantly smaller than 1). Consequently, we can conclude that our SOEC lab‐prototype with a pure GDC electrode excellently meets the requirements for online coupling with carbonylation reactions.

### Studying the Suitability of CO in CO_2_ as a Feed Gas for Carbonylation

In order to know the operating point of the SOEC, the optimal CO/CO_2_ ratio for carbonylation reactions was determined ex situ by using CO/CO_2_ mixtures generated with mass flow controllers (MFC). We initially worked on the alkoxycarbonylation of 1‐octene under batch‐wise conditions in alcoholic solutions, employing Pd_2_(dba)_3_ as precursor complex, 1,2‐bis(di‐*tert*‐butylphosphinomethyl) benzene (dtbpx) as ligand, and methanesulfonic acid as proton source.^[^ ^52^ ^]^

**Figure 3:**
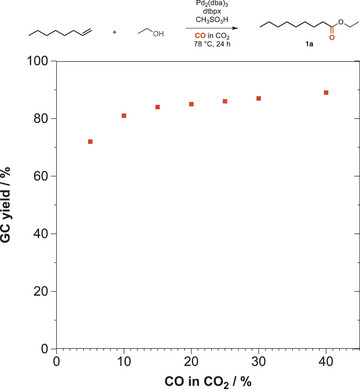
Synthesis of ethyl nonanoate (**1a**) with varying CO concentrations.

Several bottlenecks may arise in a conventional batch‐wise process involving gas bubbling into a reaction mixture with <10 mL solvent over an extended period of time. Obvious issues could be loss of solvent through gas saturation or the volatility of the product ester. While screening different reaction conditions, it was found that the concentration of 1‐octene played a critical role in getting higher yields (Table S1). Eventually, a substrate concentration of 1.66 M in ethanol as solvent and nucleophile at a gas flow rate of 11 mL min^−1^ was found as optimum, allowing to isolate ethyl nonanoate in a high yield of 80%. Once the experiments were optimized to overcome all practical bottlenecks, the concentration of CO in CO_2_ was varied to resolve the ideal composition of the employed gas mixture (Figure 3).

Variation of the CO content showed that a comparingly small content of CO in CO_2_ is sufficient to reach complete conversion; in fact, an increase of CO in CO_2_ beyond 15% only negligibly increased the yield of the obtained ester. Moreover, this behavior is not limited to aliphatic alkoxycarbonylations but has also been shown for the carbonylative synthesis of aromatic esters starting from aryl halides and phenols that are essential building blocks for a plethora of pharmaceuticals or agrochemicals.^[^ ^53^, ^54^, ^55^, ^56^ ^]^ Under slightly modified literature parameters^[^ ^57^ ^]^ in DMF, phenyl benzoate could be isolated in 52% using 15% CO in CO_2_ only (Figure S11). This is a robust finding in light of a continuous process, as it allows a reaction design with partial CO_2_ reduction and in situ consumption of the formed CO in a streamlined carbonylation process.

### Online Coupling of CO Generation in the SOEC with Continuous Carbonylation Reactions

With all required puzzle pieces optimized and in hand, we turned our attention to the design of a continuous carbonylation process in combination with high‐temperature electrolysis of CO_2_.

**Figure 4:**
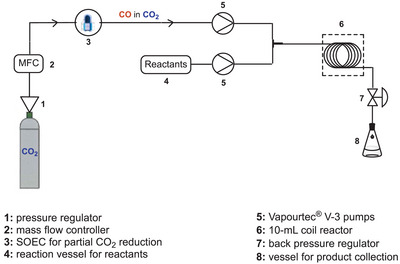
Schematic presentation of the continuous process.

To overcome the inherent limitations associated with batch processes,^[^ ^19^, ^21^, ^25^ ^]^ we opted for a continuous‐flow coil reactor with a high surface‐to‐volume ratio for shorter reaction times, improved yields, and precise dosing of reactants (Figure 4). Furthermore, due to the larger interfacial surface area in continuous mode, the gas availability in the liquid phase can be improved, potentially enhancing the gas solubility as otherwise inherent limitation.^[^ ^58^ ^]^

The coil reactor's geometry is very similar to the so‐called gas‐liquid tube‐in‐tube reactor, regularly used in continuous reactions for gas introduction. However, a gas‐liquid reactor is typically designed for hydrogenation, and molecules such as CO, CO_2_, or O_2_ are too big to pass the reactor's membrane. Additionally, the electrochemical cell operated with a gas output of ≈10 mL min^−1^; therefore, a coil reactor with a larger volume (10 mL) seemed a reasonable choice.

It is worth mentioning that residence time calculation for gas‐liquid systems above atmospheric pressure is challenging, as the gas gets dissolved in the liquid phase during the reaction. The gas consumption leads to the reduction of the gas flow rate and thus to increased residence time. On the other hand, the thermal expansion of gases results in shorter residence times at elevated temperatures.^[^ ^59^ ^]^ Therefore, the term “residence time” will be avoided, and reaction time will be used instead (calculated from the reaction mixture volume (10 mL) and the respective flow rate).

**Figure 5:**
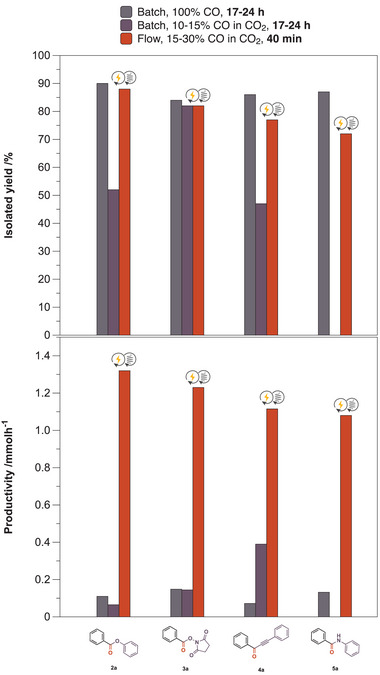
Phenoxycarbonylation (**2a**), synthesis of redox‐active esters (**3a**), carbonylative Sonogashira coupling (**4a**), and aminocarbonylation (**5a**) demonstrating the advantages of electro‐flow carbonylation with a SOEC in continuous flow (orange) compared to conventional processing (gray, purple). Isolated yields (top) and productivities (bottom) as metrics. Product **5a** could not be isolated in batch mode with low CO content.

At first, we sought to investigate the phenoxycarbonylation reaction in continuous mode by employing iodobenzene as substrate and phenol as the nucleophile. With respect to the product's polarity, in order to avoid obstruction, acetonitrile was used as the solvent (Table 1). We initially worked with a relatively high CO content of 50%, which resulted in complete conversion within 20 min reaction time (Table 1, entry 2). Once again, it should be pointed out that the online provision of such high CO concentrations is only feasible using an SOEC with the special pure GDC cathode, which underlines the uniqueness of the approach we have chosen. We further aimed to reduce the CO content to a possible minimum. 40% CO content still provided complete conversion within 20 min (Table 1, entry 3), but the reactivity dropped when mixtures with lower CO content were employed (Table 1, entries 4, 5). The temperature proved to be a less influential parameter of the reaction; hence, its increase by 15 °C only improved the conversion by 4% (Table 1, entry 4 vs 6). With an extended reaction time of 40 min and increased gas mixture flow rate, a CO content of 15% proved sufficient to provide complete conversion (Table 1, entry 9), which is in excellent agreement with the results from our batch‐mode screening reactions shown above. Ultimately, the product, phenyl benzoate (Figure 5, **2a**) was isolated with a high yield of 88%.

**Table 1:** Optimization of the phenoxycarbonylation of iodobenzene in continuous mode.

Entry^a)^	CO Content / %	Temperature / %	Reaction Flow Rate /µl min^−1^	Gas Mixture Flow Rate /mL min^−1^	Conversion / %^c)^
1^b)^	15	90	500	2.0	23
2	50	95	500	3.0	>99
3	40	95	500	3.0	>99
4	30	95	500	3.0	89
5	20	95	500	3.0	69
6	30	110	500	3.0	93
7	30	95	500	3.5	97
8	30	95	333	3.0	>99
9	15	100	250	3.6	>99
10	15	100	333	3.0	66
11	15	100	333	4.5	92

^a)^ The reactions were performed with 1 mmol iodobenzene, 2 mmol phenol, 0.03 mmol Pd(OAc)_2_, 0.03 mmol Xantphos, and 3 mmol Et_3_N in 10 mL acetonitrile; the BPR was set to 3 bar.

^b)^ Performed in 10 mL toluene with 5 mmol iodobenzene.

^c)^ Determined by GC‐MS analysis.

After establishing the continuous process for phenoxycarbonylations, we employed the gained experience for other reactions to broaden the scope of the process and take full advantage of the tunability and variable CO content produced by the electrochemical cell. Most recently, many strategies have been reported for using redox‐active esters (RAEs) as radical precursors in the emerging field of radical chemistry.^[^ ^60^, ^61^ ^]^ Carboxylic acid esters of *N*‐hydroxysuccinimide (NHS) have been utilized as invaluable intermediates in the synthesis of peptides^[^ ^62^ ^]^ and nucleotides.^[^ ^63^ ^]^ Their synthesis via carbonylative cross‐coupling was reported by Lou and co‐workers in 2003.^[^ ^64^ ^]^ Under the standard literature conditions, at a 3 mmol scale, employing a pre‐formed CO/CO_2_ mixture and iodobenzene as substrate, the transformation gave an excellent yield of 84% in batch mode, although a long reaction time of 17 h was required (Figure S13). The developed continuous process was versatile and equally applicable to RAE synthesis; in fact, the online coupling of the SOEC with a coil reactor yielded the desired product with 82% in 40 min only (Figure 5, **3a**). Similarly, shortened reaction times and high yields were found for carbonylative Sonogashira couplings (Figure 5, **4a**), thus demonstrating the advantages and versatility of the streamlined electrochemical set‐up with the continuous conversion of CO generated by the electrochemical cell.

The developed continuous process proved suitable for *C‐* and *O‐*nucleophiles. However, we further aimed to construct the much more challenging amide bond via carbonylation. The importance of these bonds cannot be accentuated enough; hence, the amide motif can be found in countless biologically active molecules, agrochemicals, or physiologically essential peptides.^[^ ^65^, ^66^, ^67^ ^]^ After carrying out several preliminary experiments, an approach similar to the previously utilized methods was identified for aminocarbonylations relying on Pd(OAc)_2_, and Xantphos in the presence of Et_3_N (Table S2). Initially, harsh conditions, such as 120 °C and a CO content of 50%, were required to force the reaction. Interestingly, the conversion did not drop significantly when the CO content was decreased to 30%, and the reaction was carried out at 110 °C. Moreover, the continuous method could be performed without employing any ligand, and the product, benzanilide (Figure 5, **5a**), could be isolated with a good yield of 72%.

**Figure 6:**
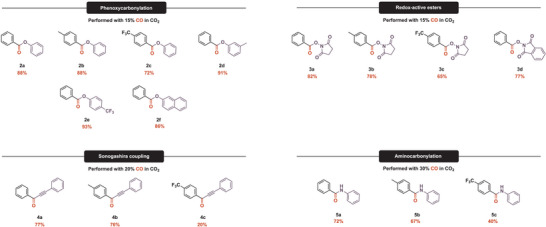
Scope of the continuous carbonylations with CO/CO_2_ mixtures.

Eventually, we broadened the substrate scope to provide a large pool of (redox‐active) esters, amides, and ketones (products **2a‐5c** in Figure 6). Overall, the corresponding products were isolated with good to excellent yields in short reaction times, which is a significant improvement compared to the results from the experiments conducted in batch mode. The lower yields obtained in the aminocarbonylations (Figure 6, **5a–c**) can be explained by an additional acidic workup step. Due to the similar eluting properties, the excessive nucleophile had to be removed by acidic extraction, and product loss may occur during that step. It is worth mentioning that 2‐naphthyl benzoate (Figure 6, **2f**), which is also known for its intestinal antiseptic property, could be isolated with an excellent yield of 86%. Substrates bearing the electron‐withdrawing trifluoromethyl group proved much less reactive, as the corresponding products (Figure 6, **2c, 3c, 4c, 5c**) were isolated with lower yields. The formation of the corresponding non‐carbonylative Sonogashira product can explain the inferior yield of compound **4c**.

## Conclusion

We successfully employed a solid oxide electrolysis cell (SOEC) to perform dry CO_2_ reduction. Online coupling the SOEC with a continuous flow set‐up allowed for the streamlined utilization of the formed CO in carbonylation processes. One key factor for this success is the introduction of a pure GDC cathode into the high‐temperature cell, delivering high performance without the need for otherwise state‐of‐the‐art nickel. This configuration allows the dry electrolysis of CO_2_ without degradation due to coke formation at the SOEC cathode. Therefore, without the need to add water vapor to the feed gas, the CO/CO_2_ mixture produced meets the stringent criteria for carbonylations, facilitating the online provision of CO_2_ as a C1 building block in these reactions.

The developed process demonstrated widespread applicability in amino‐, alkoxy‐, and phenoxycarbonylations, in the synthesis of redox‐active esters, and in carbonylative Sonogashira couplings. The applicability of flow chemistry technologies revolutionizes carbonylation reactions as they render the overall process less time‐consuming and provide a tunable and easily scalable synthesis of these carbonyl compounds. Moreover, this continuous synthesis process seamlessly integrates with high‐temperature CO_2_ electrolysis, thus forming the foundation for the efficient conversion of onsite generated CO. Ultimately, this strategy presents a novel platform for minimizing the need for large quantities of CO and facilitates the production of carbonyl compounds, able to circumvent problems associated with the current state of the art.

## Supporting Information

The authors have cited additional references within the Supporting Information.^[^ ^68^, ^69^, ^70^, ^71^, ^72^, ^73^, ^74^, ^75^, ^76^, ^77^, ^78^, ^79^, ^80^, ^81^, ^82^, ^83^, ^84^, ^85^, ^86^, ^87^, ^88^, ^89^, ^90^, ^91^, ^92^, ^93^, ^94^, ^95^ ^]^

## Conflict of Interests

The authors declare no conflict of interest.

## Supporting information



Supporting Information

## Data Availability

The data that support the findings of this study are available in the Supporting Information of this article.
