# Supplementary material for: Online Coupling High‐Temperature Electrolysis with Carbonylation Reactions: A Powerful Method for Continuous Carbon Dioxide Utilization
Source: Angew Chem Int Ed Engl. 2025 Apr 7;64(22):e202420578. doi: 10.1002/anie.202420578 (PMC12105702; doi:10.1002/anie.202420578)
Supplement: Supplementary file 1 — Supporting Information [file ANIE-64-e202420578-s001.pdf]

Supporting Information

Online Coupling High-Temperature Electrolysis with Carbonylation Reactions: A Powerful Method for Continuous Carbon Dioxide Utilization

Kristof Stagel,[a,†] *Kirsten Rath,*[b,†] *Prasad M. Kathe,*[a] *Michael Schnürch,*[a] *Tobias M. Huber,*[b] *Alexander K. Opitz,*[b,*] *and Katharina Bica-Schröder*[a,*]

[a] Institute of Applied Synthetic Chemistry, TU Wien, Getreidemarkt 9/163, 1060 Vienna, Austria

[b] Institute of Chemical Technologies and Analytics, TU Wien, Getreidemarkt 9/164, 1060 Vienna, Austria

[†] Equal contribution.

* Corresponding authors:
Katharina Bica-Schröder. E-mail: katharina.schroeder@tuwien.ac.at;
Alexander K. Opitz. E-mail: alexander.opitz@tuwien.ac.at

Table of Contents

1. General Remarks 2
2. Methods 3
3. Near Ambient Pressure XPS Measurements on Well-Defined GDC Thin-Film Electrodes 5
4. Set-Up of the Continuous-Flow Experiments 7
5. Solid Oxide Electrolysis Cell (SOEC) 8
6. Current-Voltage Curve of the High Feed Gas Flux Experiment 9
7. Chromatograms of CO_2 Electrolysis at 10 mL/min Gas Flux 10
8. Optimization of the Batch-Wise Synthesis of Nonanoic Acid Esters 11
10. Optimization of the Aminocarbonylation Reactions in Continuous Mode 12
11. Procedure for the Synthesis of Ethyl Nonanoate 14
12. Procedure for the Batch-Wise Synthesis of Phenyl Benzoate 15
13. General Procedure for the Continuous Synthesis of Benzoic Acid Ester Derivatives 16
14. Procedure for the Batch-Wise Synthesis of *N*-(Benzoyloxy)-succinimide 18
15. General Procedure for the Continuous Synthesis of Redox-Active Esters 19
16. Procedure for the Batch-Wise Synthesis of Diphenylpropynone 21
17. General Procedure for the Continuous Carbonylative Sonogashira Couplings 22
18. General Procedure for the Continuous Aminocarbonylations 24
19. NMR Spectra of 1a-5c 26
20. References 46

1. General Remarks

Unless otherwise noted, all purchased chemicals from commercial suppliers were used without further purification.

Column chromatography was performed on standard glass columns using Merck (40-60 μm) silica gel with pre-distilled solvents. For TLC analysis, pre-coated aluminum-backed plates were used (Merck, silica gel 60 F_{254}). All compounds were detected at 254 nm.

^{1}H-, ^{13}C- and ^{19}F spectra were recorded from $CDCl_3$ or $(CD_3)_2SO$ solutions on a Bruker Avance UltraShield 400 MHz (^{1}H: 400 MHz, ^{13}C: 101 MHz, ^{19}F: 376 MHz) NMR instrument. Chemical shifts are reported in parts per million (ppm) and were calibrated to the residual solvent signal (e.g., $CDCl_3$, ^{1}H: 7.26 ppm, ^{13}C: 77.0 ppm). Coupling constants are reported in hertz (Hz). The assignments are based on the comparison with reported spectra.

GC analysis was performed on a Thermo Scientific Focus, employing a BGB5 column using an FID detector. A linear temperature program was used, starting with a temperature of 50 °C, followed by a ramp rate of 15°C/min up to a final temperature of 240 °C. For the optimization of the synthesis of ethyl nonanoate, the yield has been determined by using *n*-dodecane as internal standard.

Continuous-flow experiments were performed with the aid of a Vapourtec® E-Series flow chemistry device.

Mass flow controllers were purchased from Brooks Instruments; SLA5850 series with four channel secondary electronics (Model 0254). The MFCs were purchased with a configuration that allowed a maximum inlet pressure of 7.5 atm and an outlet pressure of 1 atm. Carbon monoxide and carbon dioxide were provided from the respective cylinders equipped with a low-pressure regulator, outputting 5 bar (2 bar when the electrochemical cell was employed). For the continuous-flow experiments, the gases were introduced using a V-3 pump after passing through the gas mixer.

2. Methods

Fabrication of the Solid Oxide Electrolyzer

For preparing the SOEC employed for the *in situ* supply of CO, a dense sintered tube made of yttria-stabilized zirconia (YSZ, purchased from McDanel Advanced Ceramics), which was closed on one side, was used as electrolyte. On the outer side of the tube, the cathode was applied as a three-layer electrode by depositing pastes of GDC, GDC/Pt, and Pt, similar to the electrodes used by Nenning *et al*.[1] Located directly adjacent to the electrolyte, the electrochemically active layer consisted of porous pure GDC (Treibacher, AT). The topmost porous Pt layer acts as an electronic current collector, and it has already been proven that this layer does not contribute any catalytic activity to the electrode[1] owing to its distance to the electrode/electrolyte interface. A GDC/Pt composite layer was applied between the GDC and Pt layers, which ensures a gradual transition between the two materials and thus provides good adhesion. (Please note that for commercial applications, Ni instead of Pt would be a much more suitable choice, but for the lab-based application here, Pt paste was used for convenience.)

For the air electrode, Pt/YSZ paste was applied to the inner side of the tube. After both electrodes were applied in the form of pastes, the whole assembly was sintered in air at 1200°C for 3 h. In order to operate the cell obtained in this way as SOEC, the tube was bonded with the open side into a brass KF40 flange. The air electrode was contacted with a thick silver wire, and the CO_2-splitting electrode with a Ni wire. The entire SOEC was capped with a one-side closed quartz tube that fits as closely as possible to minimize dead volume. In order to bring the SOEC to its operating temperature of 750°C, the entire set-up was heated with a tube furnace.

For the *in situ* production of CO, the outer compartment of the SOEC was supplied with CO_2 via a mass flow controller, and the GDC electrode was cathodically (i.e. negatively) polarized. The resulting CO concentration at the SOEC outlet is a result of the gas flux and the electrochemical current. For example, a gas flux of 10 mL CO_2/min and an electrochemical current of 195 mA result in a gas composition of 15 vol% CO and 85 vol% CO_2. For the examination of the Faraday efficiency of the cell, please refer to the results section.

Model-type Cells for Studying the Kinetics of Pure GDC Electrodes

Additional model-type samples were prepared to explore the electrochemical behaviour of the GDC cathode alone and to demonstrate their coking resilience. They consist of a single crystalline YSZ electrolyte and porous, spin-coated, pure GDC working electrode with a porous Pt current collector on top, which is identical to the cathodes used in the SOEC described above and the Ni-free GDC electrodes

mentioned before.[1] As counter electrode and reference electrode, layers of YSZ/Pt and Pt pastes were applied to the other side of the YSZ single crystal. An X-ray diffraction pattern of the counter electrode is depicted in Fig. S1, which only shows reflections from the ceramic electrode GDC and the Pt current collector.

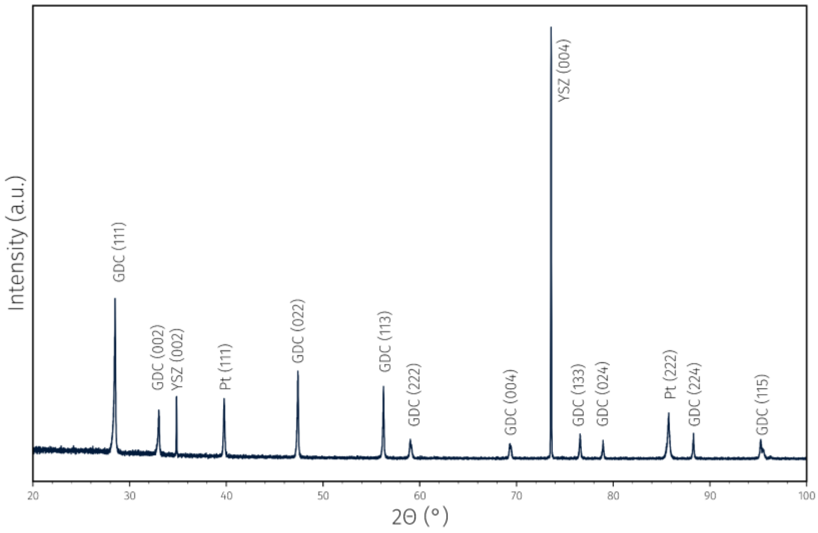

Fig. S1. Diffraction pattern of the GDC electrode with Pt-current collector in model-type cells.

The relationship between the electrochemical current I and the flux $\dot{V}$ of the product CO is given by Faraday's law

$$It = nzF \tag{S1}$$

which, by inserting the ideal gas equation ($pV = nRT$) and substituting $\dot{V} = V/t$ reads

$$I = \dot{V}\frac{p}{RT}zF \tag{S2}$$

In **Eqs. S1** and **S2**, t denotes the time, n the number of moles, z the number of transferred charges (which is 2 for the reaction in **Eq. S1**), p the pressure, V the volume, and T the temperature. R and F are the ideal gas constant and Faraday's constant, respectively. The faradaic efficiency FE is given by the ratio of the product flux (converted to a current I_{calc} by **Eq. S2**) and the measured respective DC current I_{DC} (**Eq. S3**):

$$FE = \frac{I_{calc}}{I_{DC}} \tag{S3}$$

3. Near Ambient Pressure XPS Measurements on Well-Defined GDC Thin-Film Electrodes

To demonstrate that the GDC used here with the composition $Ce_{0.9}Gd_{0.1}O_{1.95}$ has the same surface chemistry as the CeO_2-based materials investigated in the literature, near ambient pressure X-ray photoelectron spectroscopy (NAP-XPS) measurements were carried out on GDC thin-film electrodes with an embedded current collector. The model-type thin-film electrodes were required, as the electrochemically active zone of our real, porous GDC electrodes is spectroscopically not accessible by XPS due to its relatively low information depth (inelastic mean free path of photoelectrons) of about 2 nm.

The fabrication of the model cells with thin-film GDC working electrode was produced in the same way as in our previously reported *in situ* XPS and GDC model electrode experiments – see Refs [2]. A sketch of the sample used is shown in Fig. S2. - for further details on the instrument please refer to Rameshan *et al*.[3] Examples of the O1s spectra recorded on GDC thin-film electrodes that were cathodically polarized in 1 mbar CO/CO_2 are shown in Fig. S3. In these spectra, upon sufficiently cathodic polarization (-500 mV, bottom panel) the evolution of a carbonate-type intermediate at ca. 532 eV binding energy (denoted O_{ads}) is clearly visible, very similar to literature studies on high-temperature CO_2 splitting employing Sm-doped CeO_2.[4]

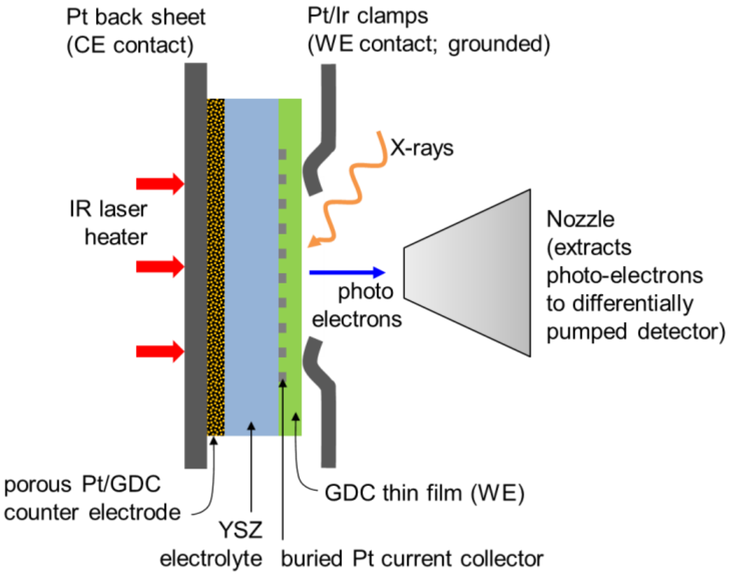

Fig. S2. Schematic cross section of a sample with a GDC thin-film electrode featuring a buried Pt current collector in the NAP-XPS set-up.

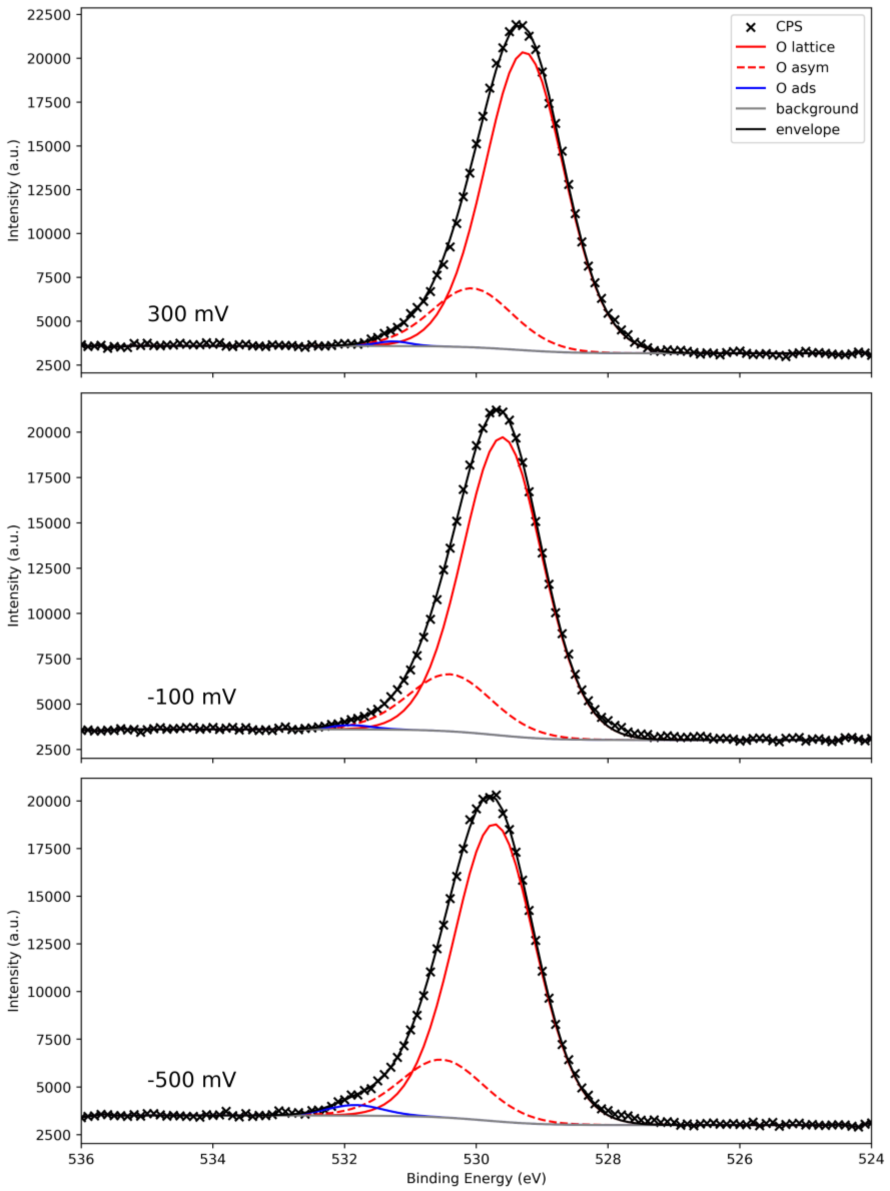

Fig. S3. O1s XPS spectra recorded on GDC thin-film electrodes in 1 mbar of a 1:10 mixture of CO:CO_2 at different applied voltages. At the strongest cathodic polarization, an additional feature at ca. 532 eV binding energy is clearly visible.

4. Set-Up of the Continuous-Flow Experiments

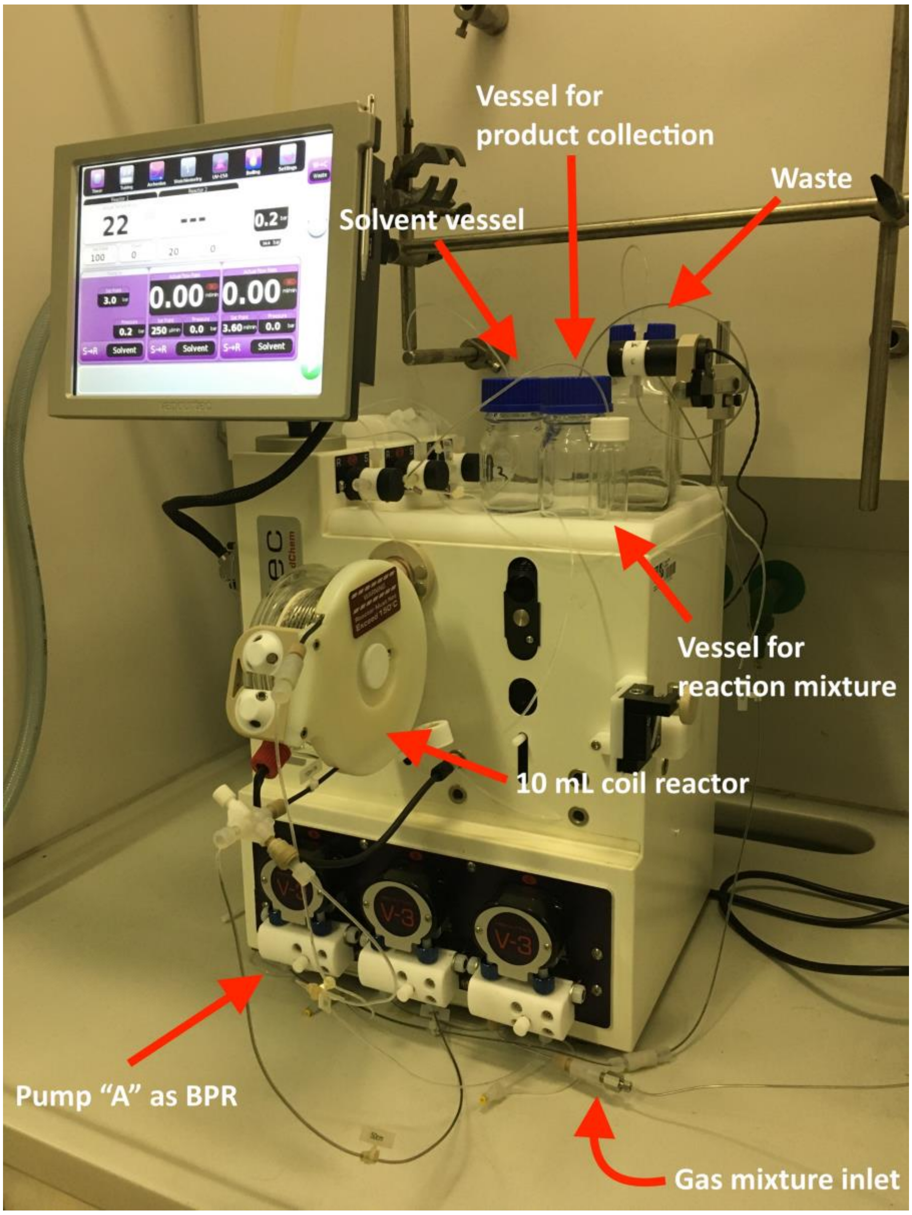

Figure S4. Set-up of the device operating in continuous mode.

5. Solid Oxide Electrolysis Cell (SOEC)

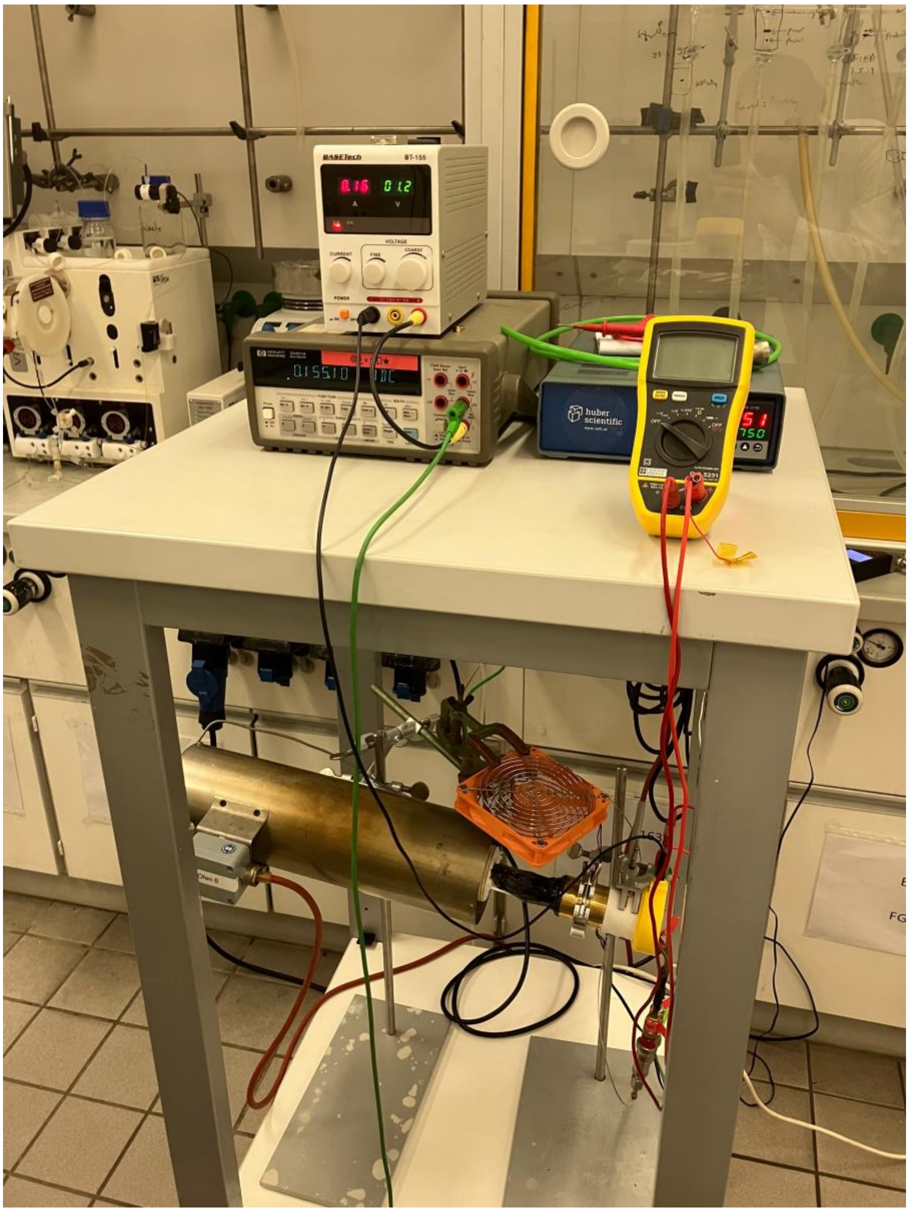

Figure S5. Solid oxide electrolysis cell for partial CO_2 reduction.

6. Current-Voltage Curve of the High Feed Gas Flux Experiment

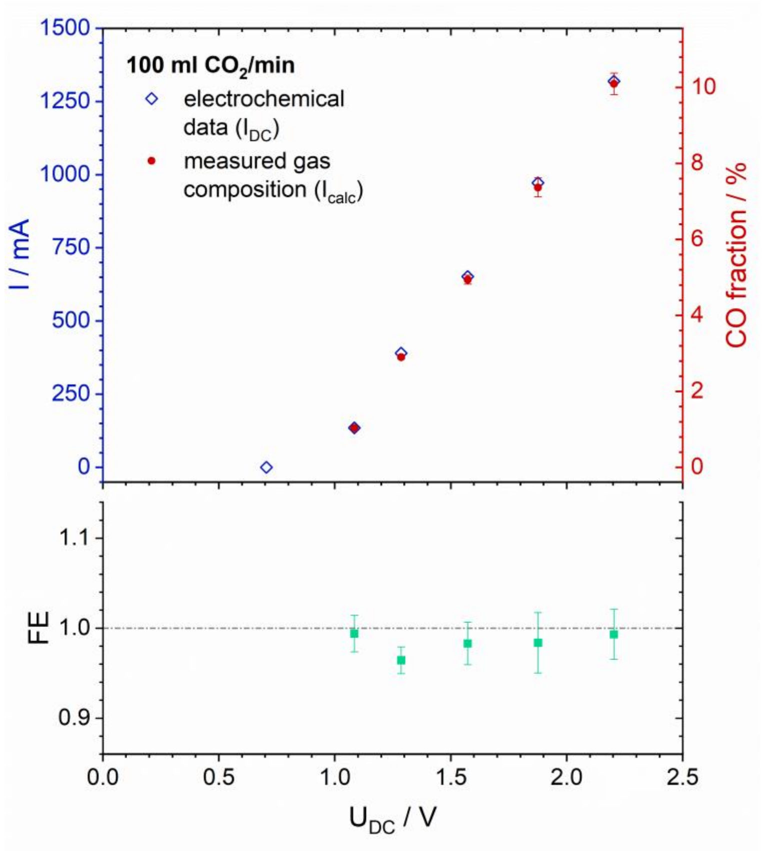

Figure S6. Comparison of the recorded I-V-curve and the resulting CO concentration for feeding 100 ml CO_2/min to the lab SOEC.

7. Chromatograms of CO_2 Electrolysis at 10 mL/min Gas Flux

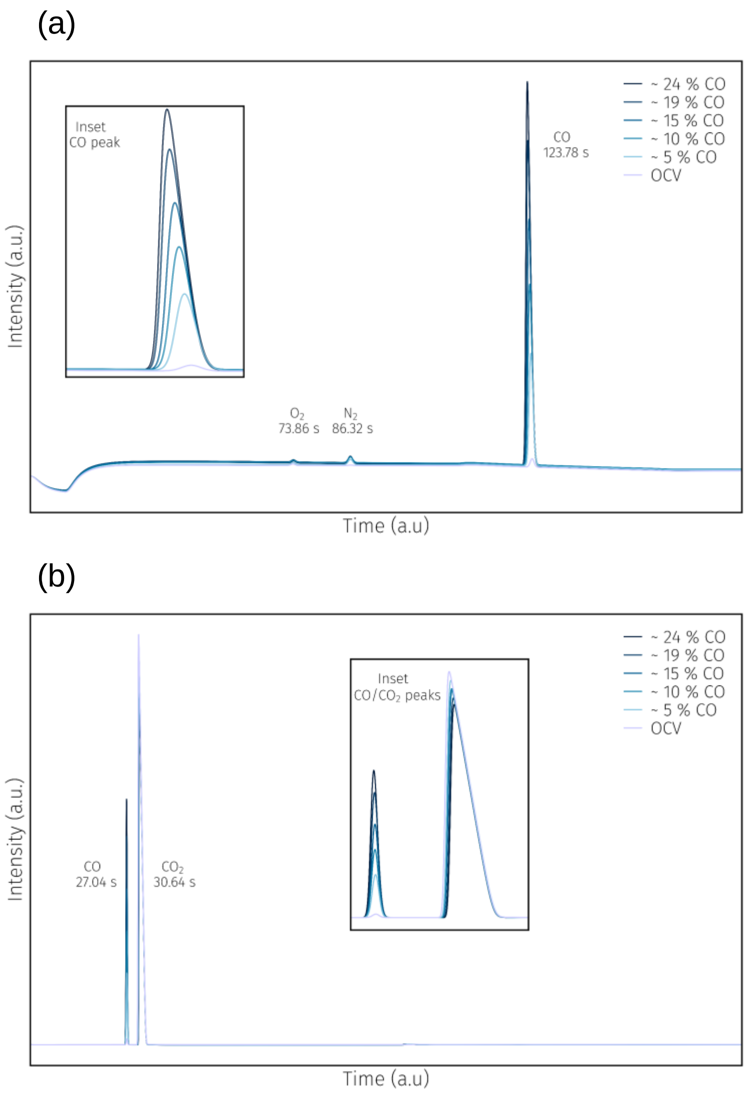

Fig. S7. Chromatograms of gas leaving the SOEC lab prototype with our pure GDC cathode at a flux of 10 mL/min corresponding to the data in Fig. 2b. (a) Signal intensity recorded with a micro-GC module employing a mol-sieve-column for gas separation. In this case, the CO_2 is separated by a backflush system before injection. Consequently, only the CO (and air traces) are visible. (b) Signal intensity recorded with a micro-GC module employing a CO_2-compatible column, thus showing both gas species.

8. Optimization of the Batch-Wise Synthesis of Nonanoic Acid Esters

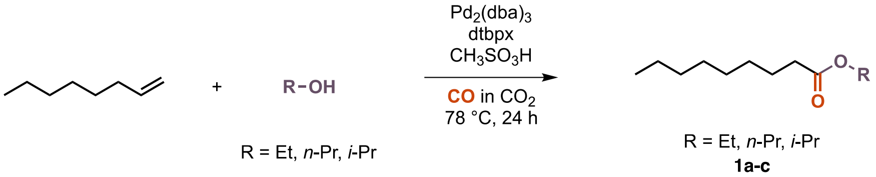

Figure S8. Synthesis of nonanoic acid esters.

Table S1. Optimization of the synthesis of nonanoic acid esters.

Entry[a]	Solvent/ Nucleophile	Substrate concentration /moldm^{-3}	CO flow rate /mLmin^{-1}	CO_2 flow rate /mLmin^{-1}	Yield[f] /%
1	*i*-PrOH	0.25	2.5	40	6
2	*n*-PrOH	0.25	2.5	40	14
3	*n*-PrOH	0.25	6.0	30	22
4	*n*-PrOH	0.75	1.0	30	39
5	*n*-PrOH	0.25	1.0	25	13
6	*n*-PrOH	0.25	1.0	10	8
7[b]	*n*-PrOH	0.25	1.0	10	16
8[c]	*n*-PrOH	0.25	2.5	40	9
9	*n*-PrOH	1.00	1.5	20	49
10	*n*-PrOH	1.66	1.5	20	58
11[d]	EtOH	1.66	1.0	10	79
12[d][e]	EtOH	1.66	1.0	10	46

[a] Performed with 0.8% $Pd_2(dba)_3$, 4 mol% dtbpx, 12 mol% $MeSO_3H$ at 78 °C, reaction time: 24 h. [b] Double catalyst, ligand, and acid loading. [c] Solvent recharged after 18 h (1 mL). [d] 8 mol% $MeSO_3H$. [e] Gas not bubbled but saturated gas passed from an intermediate solvent reservoir. [f] Determined by GC analysis using dodecane as internal standard.

10. Optimization of the Aminocarbonylation Reactions in Continuous Mode

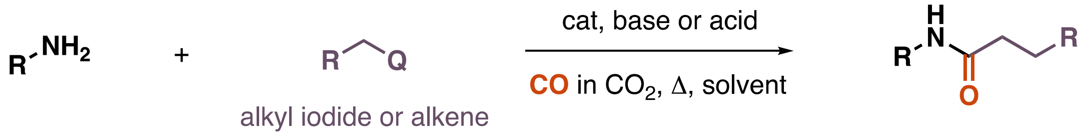

Figure S9. Aminocarbonylation parameter screening.

Table S2. Parameter screening of aminocarbonylations.

Entry	Reactants	CO amount /%	Temperature, reaction time, catalyst, base/acid, additive, solvent	Conversion[e] /%
1[a][5]	Pyrrole, iodobenzene	50	90 °C, 24 h, $Pd(PPh_3)_4$, K_3PO_4, DMF	66[f]
2[a]	Pyrrole, iodobenzene	15	90 °C, 24 h, $Pd(PPh_3)_4$, DBU, DMF	0
3[a]	Pyrrole, iodobenzene	15	90 °C, 24 h, $Pd(PPh_3)_4$, Et_3N, DMF	33[f]
4[a]	Pyrrole, iodobenzene	15	90 °C, 24 h, $Pd(PPh_3)_4$, Et_3N, toluene	0
5[a][6]	Pyrrole, iodobenzene	15	100 °C, 24 h, $Pd(OAc)_2$, cataCXium® A, TMEDA, DMF	0
6[b][5]	Pyrrole, iodobenzene	15	60 °C, 24 h, $Pd(OAc)_2$, PPh_3, Et_3N, THF	0
7[b][7]	Aniline, iodobenzene	15	100 °C, 24 h, $Pd(OAc)_2$, [BMIM]Cl, KO^{t}Bu, Et_3N, MeCN	0
8[b]	Pyrrole, iodobenzene	15-50	90 °C, 40 min, $Pd(PPh_3)_4$, Et_3N, toluene	0
9[b][8]	Aniline, 1-octene	15	150 °C, 40 min, $Pd(OAc)_2$, dtbpx, $MeSO_3H$, NaI, β-napthol, toluene	0
10[b]	Aniline, 1-octene	15	100 °C, 40 min, $Pd(OAc)_2$, Xantphos, $MeSO_3H$, NaI, β-napthol, toluene	0
11[b][c]	Aniline, iodobenzene	15	100 °C, 40 min, $Pd(OAc)_2$, Xantphos, Et_3N, MeCN	0
12[b][d]	Aniline, iodobenzene	15	100 °C, 40 min, $Pd(OAc)_2$, Xantphos, Et_3N, MeCN	0
13[b][c]	Aniline, iodobenzene	50	120 °C, 40 min, $Pd(OAc)_2$, Xantphos, Et_3N, MeCN	>99 (57[f])
14[b][c]	Aniline, iodobenzene	30	110 °C, 40 min, $Pd(OAc)_2$, Xantphos, Et_3N, MeCN	91

15[b]	Aniline, iodobenzene	30	110 °C, 40 min, $Pd(OAc)_2$, Et_3N, MeCN	94 (71[f])
16[b]	Aniline, iodobenzene	30	110 °C, 40 min, $Pd(OAc)_2$, MeCN	0

[a] Performed under batch-wise conditions. [b] Performed in continuous mode. [c] Performed with 5 mol% $Pd(OAc)_2$ and 10 mol% Xantphos. [d] Performed with 3 mol% $Pd(OAc)_2$ and 6 mol% Xantphos. [e] Determined by GC-MS analysis. [f] Isolated yields.

11. Procedure for the Synthesis of Ethyl Nonanoate

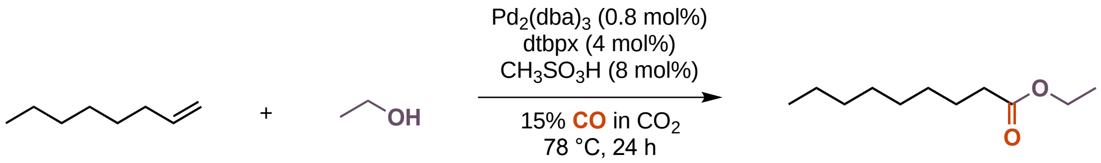

Figure S10. Synthesis of ethyl nonanoate.

In a 3-neck round-bottom flask equipped with a stir bar, tris(dibenzylideneacetone)dipalladium (51 mg, 0.8 mol%) was added, followed by 1,2-bis((di-tert-butylphosphaneyl)methyl)benzene (110 mg, 4 mol%) inside a glove box. Dry ethanol (6 mL) was then added. Following this, the substrate 1-octene (1.1 mL) was added. Finally, methanesulfonic acid (36 µL, 8 mol%) was added to the reaction vessel. The flask was then disconnected from the Argon line, and a quick-fit fitted with a septum, through which a 1/8'' steel tubing was passed, was connected. The reaction vessel was then connected to a cryostat chilled (chilled to a temperature below -5 °C) condenser. The tubing at the gas outlet from the CO_2 electrolysis set-up (155 mA corresponding to 15% CO content) went on to the 1/8'' steel tubing. The 1/8'' steel tubing was dipped in the reaction vessel in a manner that constant bubbling was seen, and there was no hindrance to the stirring bar. A stirring speed of 200 rpm was employed. The 3-neck round-bottom flask was then placed in a preheated oil bath (at 78 °C). After 24 h, the reaction mixture was cooled down to room temperature and transferred to a round-bottom flask for bulb-to-bulb distillation. Initially, at a vacuum of 900 mbar, the round-bottom flask was heated to 90 °C to remove the ethanol. After the complete removal of ethanol, the vacuum was slowly increased to 100 mbar and finally to 25 mbar. Thereafter increasing the temperature to 125 °C resulted in the product ethyl nonanoate being distilled off. The product was obtained as a colourless oil (82%, 1.06 g) and was confirmed analytically to be pure. The spectral data matches the one reported in the literature.[9]

Ethyl nonanoate (1a)

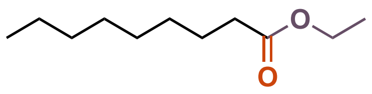

^{1}**H NMR** (400 MHz, $CDCl_3$) δ 4.11 (q, *J* = 7.1 Hz, 2H), 2.27 (t, *J* = 7.6 Hz, 2H), 1.61 (t, *J* = 7.3 Hz, 2H), 1.26 (dt, *J* = 14.2, 6.4 Hz, 13H), 0.92 – 0.82 (m, 3H). ^{13}**C NMR** (101 MHz, $CDCl_3$) δ 174.05, 60.26, 34.53, 31.94, 29.36, 29.29, 29.25, 25.13, 22.77, 14.38, 14.21.

12. Procedure for the Batch-Wise Synthesis of Phenyl Benzoate

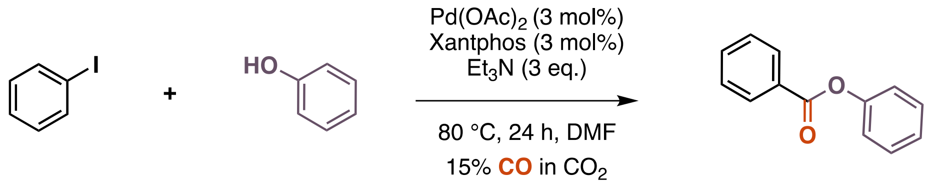

Figure S11. Synthesis of phenyl benzoate.

$Pd(OAc)_2$ (3 mol%, 13,4 mg) followed by Xantphos (3 mol%, 52 mg) and iodobenzene (3 mmol, 612 mg), phenol (570 mg, 6 mmol) were added to a 3-neck round-bottom flask under argon counterflow. This was immediately followed by the addition of dry DMF (6 mL). Finally, the base triethylamine (9 mmol, 1.25 mL) was added, and the flask containing the reaction mixture was attached without delay to the steel tubing coming from the electrochemical cell (155 mA corresponding to 15% CO content). Upon equipping the reaction flask with a condenser and ensuring a constant gas flow, the reaction vessel was lowered into a pre-heated oil bath at 80 °C and maintained at this temperature for 24 h. A stirring speed of 200 rpm was employed. The reaction mixture was diluted with 10 mL ethyl acetate and transferred to a separating funnel. The reaction mixture was swirled to ensure that the product was well distributed in ethyl acetate/DMF. Following this, the organic phases were washed with 5-6 mL of water 4 to 5 times till no more emulsion resulting from DMF in the aqueous phase was seen. The aqueous phase was then re-extracted to check if any product remained behind. The organic phase was dried with $MgSO_4$ and filtered. Rotary evaporation of the solvent gave the crude product, which was bound to silica and subjected to column chromatography with PE/Et_2O 95:5. The final pure product was isolated as a white solid (309 mg, 52%).

13. General Procedure for the Continuous Synthesis of Benzoic Acid Ester Derivatives

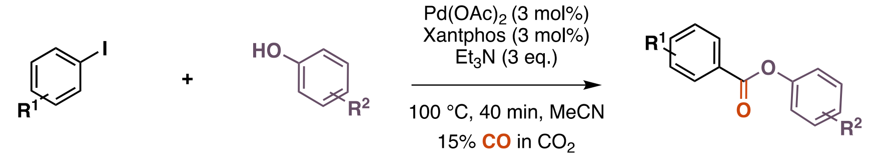

R^1 = H, *p*-CH_3, *p*-CF_3

R^2 = H, *m*-CH_3, *p*-CF_3, C3=C4-CH=CH-CH=CH-C3

2a: R^1 = H, R^2 = H,
2b: R^1 = *p*-CH_3, R^2 = H,
2c: R^1 = *p*-CF_3, R^2 = H,
2d: R^1 = H, R^2 = *m*-CH_3,
2e: R^1 = H, R^2 = *p*-CF_3
2f: R^1 = H, R^2 = C3=C4-CH=CH-CH=CH-C3

Figure S12. Synthesis of compounds **2a-f**.

A 30-mL vial with septum was charged with the corresponding aryl iodide (1 eq., 1 mmol), the corresponding phenol derivative (2 eq., 2 mmol), $Pd(OAc)_2$ (3 mol%, 0.03 mmol, 6.74 mg), Xantphos (3 mol%, 0.03 mmol, 17.4 mg), and triethylamine (3 eq., 3 mmol, 418 µL). The reactants were dissolved in 10 mL acetonitrile. The solvent bottle was charged with MeCN. The reactor was heated up to the desired temperature (100 °C). Pump A was used as a back-pressure regulator (BPR, 3 bar). Pump B was connected to the vial with the reaction mixture; pump C was connected to the gas tube, where the pre-mixed gas mixture was introduced. The gases were supplied from the respective cylinders and were pre-mixed with the aid of two mass flow controllers (CO: 0.64 mL/min; and CO_2: 3.6 mL/min). Alternatively, pump C was connected to the solid-oxide electrochemical cell (155 mA, corresponding to 15% CO content), employing a flow rate of 8.0 mL/min. The tubing was primed with the reagent mixture and acetonitrile, respectively. The reactor (10-mL coil reactor) was initially rinsed by a CO/CO_2/MeCN flow for several minutes. Then, the reaction mixture was supplied to the reactor (pump B: 0.25 mL/min; pump C: 3.6 mL/min (or 8.0 mL/min if the electrochemical cell was employed)). After the whole volume of the reaction mixture was pumped through the reactor, the vial was rinsed with pure MeCN, and the residue was pumped through the reactor. The product was collected for 50 minutes. Rotary evaporation of the solvent gave the crude product, which was bound to silica and subjected to column chromatography.

Phenyl benzoate (2a)[10]

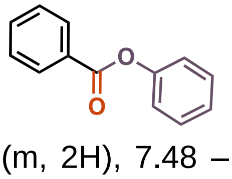

Flash column chromatography (petroleum ether/Et_2O 20:1, R_f=0.65) afforded the product as white solid (174 mg, 88%). **^{1}H NMR** (400 MHz, $CDCl_3$) δ 8.27 – 8.17 (m, 2H), 7.69 – 7.61 (m, 1H), 7.56 – 7.49 (m, 2H), 7.48 – 7.40 (m, 2H), 7.33 – 7.19 (m, 3H). **^{13}C NMR** (101 MHz, $CDCl_3$) δ 165.53, 151.11, 133.72, 130.32, 129.75, 129.64, 128.71, 126.03, 121.86.

Phenyl 4-methylbenzoate (2b)[11]

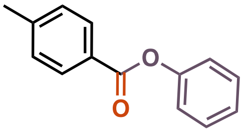

Flash column chromatography (petroleum ether/Et_2O 20:1, R_f=0.59) afforded the product as white solid (186 mg, 88%). **^{1}H NMR** (400 MHz, $CDCl_3$) δ 8.12 – 8.05 (m, 2H), 7.47 – 7.38 (m, 2H), 7.34 – 7.19 (m, 6H), 2.46 (s, 3H).**^{13}C NMR** (101 MHz, $CDCl_3$) δ 165.39, 151.18, 144.54, 130.36, 129.60, 129.43, 126.98, 125.92, 121.91, 21.90.

Phenyl 4-(trifluoromethyl)benzoate (2c)[12]

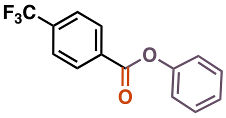

Flash column chromatography (petroleum ether/Et_2O 20:1, R_f=0.67) afforded the product as crystalline white solid (192 mg, 72%). **^{1}H NMR** (400 MHz, CDCl3) δ 8.33 (dp, J = 7.7, 0.9 Hz, 2H), 7.83 – 7.75 (m, 2H), 7.50 – 7.42 (m, 2H), 7.34 – 7.28 (m, 1H), 7.25 – 7.20 (m, 2H). **^{13}C NMR** (101 MHz, $CDCl_3$) δ 164.14, 150.81, 135.02, 132.99, 130.72, 129.76, 126.37, 125.79, 125.75, 122.36, 121.68. **^{19}F NMR** (376 MHz, CDCl3) δ -63.15.

***m*-Tolyl benzoate (2d)[13]**

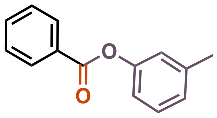

Flash column chromatography (petroleum ether/Et_2O 20:1, R_f=0.61) afforded the product as colourless liquid (194 mg, 91%). **^{1}H NMR** (400 MHz, CDCl3) δ 8.24 – 8.16 (m, 2H), 7.64 (ddt, J = 8.7, 6.9, 1.4 Hz, 1H), 7.56 – 7.47 (m, 2H), 7.32 (t, J = 7.8 Hz, 1H), 7.13 – 6.99 (m, 3H), 2.40 (d, J = 0.8 Hz, 3H). **^{13}C NMR** (101 MHz, $CDCl_3$) δ 165.44, 151.06,139.83, 133.66, 130.30, 129.82, 129.35, 128.69, 126.84, 122.45, 118.78, 21.49.

4-(Trifluoromethyl)phenyl benzoate (2e)[14]

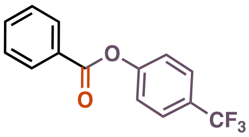

Flash column chromatography (petroleum ether/Et_2O 20:1, R_f=0.67) afforded the product as crystalline, white solid (247 mg, 93%). **^{1}H NMR** (400 MHz, CDCl3) δ 8.24 – 8.18 (m, 2H), 7.76 – 7.64 (m, 3H), 7.58 – 7.50 (m, 2H), 7.39 – 7.33 (m, 2H). **^{13}C NMR** (101 MHz, $CDCl_3$) δ 164.81, 153.63, 134.12, 130.42, 128.85, 128.18, 127.03, 122.41. **^{19}F NMR** (376 MHz, CDCl3) δ -62.20.

Naphtalen-2-yl benzoate (2f)[15]

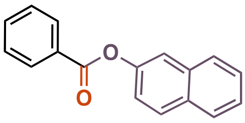

Flash column chromatography (petroleum ether/Et_2O 10:1, R_f=0.68) afforded the product as crystalline, white solid (214 mg, 86%). **^{1}H NMR** (400 MHz, CDCl3) δ 8.27 (dt, J = 8.4, 1.3 Hz, 2H), 7.95 – 7.82 (m, 3H), 7.73 – 7.70 (m, 1H), 7.70 – 7.63 (m, 1H), 7.58 – 7.46 (m, 4H), 7.38 (ddd, J = 8.9, 2.4, 1.2 Hz, 1H). **^{13}C NMR** (101 MHz, $CDCl_3$) δ 165.51, 148.75, 133.97, 133.79, 131.67, 130.37, 129.72, 129.61, 128.76, 127.95, 127.83, 126.73, 125.88, 121.39, 118.84.

14. Procedure for the Batch-Wise Synthesis of *N*-(Benzoyloxy)-Succinimide

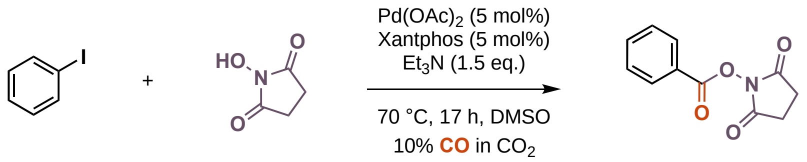

Figure S13. Synthesis of *N*-(benzoyloxy)-succinimide.

In a dry 3-neck round-bottom flask (25 mL) was added $Pd(OAc)_2$ (33,6 mg, 5 mol%), Xantphos (86 mg, 5 mol%), iodobenzene (3 mmol, 612 mg) and *N*-hydroxysuccinimide (4,2 mmol, 482 mg). The contents of the reaction vessel were dissolved in DMSO (6 mL). This was followed by the addition of triethylamine (4.5 mmol, 0.62 mmol). A mixture of CO and CO_2 was then bubbled through this mixture for 17 hours. After reaction completion, the reaction mixture was diluted with 10 mL ethyl acetate and stirred for 10 mins. The mixture was then transferred to a separating funnel. The organic phase was washed with H_2O (5 x 7 mL) and brine (1 x 1 mL). The organic phase was then dried over dry $MgSO_4$. The residue was bound to silica gel and subjected to column chromatography with PE/EtOAc 3:1 to give the product as a crystalline solid (552 mg, 84 %).

15. General Procedure for the Continuous Synthesis of Redox-Active Esters

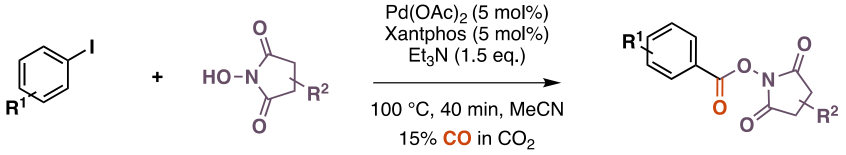

R^1 = H, *p*-CH_3, *p*-CF_3 R^2 = H, C3=C4-CH=CH-CH=CH-C3

3a: R^1 = H, R^2 = H,
3b: R^1 = *p*-CH_3, R^2 = H,
3c: R^1 = *p*-CF_3, R^2 = H,
3d: R^1 = H, R^2 = C3=C4-CH=CH-CH=CH-C3

Figure S14. Synthesis of compounds **3a-d**.

A 30-mL vial with septum was charged with the corresponding aryl iodide (1 eq., 1 mmol), the corresponding *N*-hydroxyimide (1.4 eq., 1.4 mmol), $Pd(OAc)_2$ (5 mol%, 0.05 mmol, 11.2 mg), Xantphos (5 mol%, 0.05 mmol, 28.9 mg) and triethylamine (1.5 eq., 1.5 mmol, 209 µL), and the reactants were dissolved in 10 mL acetonitrile. The solvent bottle was charged with MeCN. The reactor was heated up to the desired temperature (100 °C). Pump A was used as a back-pressure regulator (BPR, 3 bar). Pump B was connected to the vial with the reaction mixture; pump C was connected to the gas tube, where the pre-mixed gas mixture was introduced. The gases were supplied from the respective cylinders and were pre-mixed with the aid of two mass flow controllers (CO: 0.64 mL/min; and CO_2: 3.6 mL/min). Alternatively, pump C was connected to the solid-oxide electrochemical cell (155 mA, corresponding to 15% CO content), employing a flow rate of 8.0 mL/min. The tubing was primed with the reagent mixture and acetonitrile, respectively. The reactor (10 mL coil reactor) was initially rinsed by a CO/CO_2/MeCN flow for several minutes. Then, the reaction mixture was supplied to the reactor (pump B: 0.25 mL/min; pump C: 3.6 mL/min (or 8.0 mL/min if the electrochemical cell was employed)). After the whole volume of the reaction mixture was pumped through the reactor, the vial was rinsed with pure MeCN, and the residue was pumped through the reactor. The product was collected for 50 minutes. Rotary evaporation of the solvent gave the crude product, which was bound to silica and subjected to column chromatography.

2,5-Dioxopyrrolidin-1-yl benzoate (3a)[16]

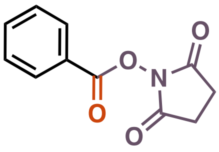

Flash column chromatography (petroleum ether/EtOAc 3:1, R_f=0.34) afforded the product as crystalline, brownish-white solid (180 mg, 82%). **^{1}H NMR** (400 MHz, DMSO) δ 8.11 – 8.04 (m, 2H), 7.87 – 7.78 (m, 1H), 7.68 – 7.60 (m, 2H), 2.88 (s, 4H). **^{13}C NMR** (101 MHz, DMSO) δ 170.34, 161.80, 135.60, 129.99, 129.57, 124.51, 25.56.

2,5-dioxopyrrolidin-1-yl 4-methylbenzoate (3b)[17]

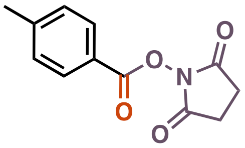

Flash column chromatography (petroleum ether/EtOAc 3:1, R_f=0.34) afforded the product as crystalline, pinkish solid (182 mg, 78%). **^{1}H NMR** (400 MHz, DMSO) δ 8.02 – 7.96 (m, 2H), 7.46 (d, J = 8.0 Hz, 2H), 2.89 (s, 4H), 2.44 (s, 3H). **^{13}C NMR** (101 MHz, DMSO) δ 170.41, 161.74, 146.46, 130.10, 130.03, 121.71, 25.55, 21.40.

2,5-dioxopyrrolidin-1-yl 4-(trifluoromethyl)benzoate (3c)[17]

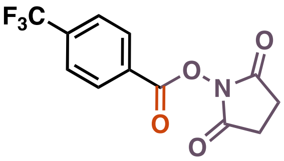

Flash column chromatography (petroleum ether/EtOAc 3:1, R_f=0.37) afforded the product as crystalline, brownish-white solid (187 mg, 65%). **^{1}H NMR** (400 MHz, DMSO) δ 8.31 (d, *J* = 8.1 Hz, 2H), 8.04 (d, *J* = 8.3 Hz, 2H), 2.91 (s, 4H). **^{13}C NMR** (101 MHz, DMSO) δ 170.14, 160.96, 134.81, 130.99, 128.32, 126.56, 124.77, 25,59. **^{19}F NMR** (376 MHz, DMSO) δ -62.03.

1,3-dioxoisoindolin-2-yl benzoate (3d)[18]

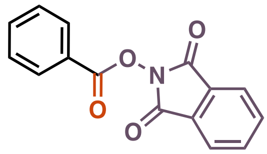

Flash column chromatography (petroleum ether/EtOAc 7:1, R_f=0.51) afforded the product as white solid (206 mg, 77%). **^{1}H NMR** (400 MHz, $CDCl_3$) δ 8.23 – 8.17 (m, 2H), 7.93 (dd, *J* = 5.5, 3.1 Hz, 2H), 7.82 (dd, *J* = 5.5, 3.1 Hz, 2H), 7.70 (ddt, *J* = 7.9, 7.1, 1.3 Hz, 1H), 7.58 – 7.50 (m, 2H). **^{13}C NMR** (101 MHz, $CDCl_3$) δ 162.96, 162.21, 135.03, 134.93, 130.80, 129.18, 129.02, 125.45, 124.17.

16. Procedure for the Batch-Wise Synthesis of Diphenylpropynone

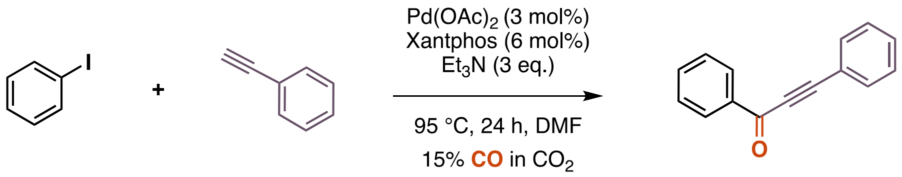

Figure S15. Synthesis of diphenylpropynone.

$Pd(OAc)_2$ (3 mol%, 13,4 mg) followed by Xantphos (6 mol%, 70 mg) and iodobenzene (2 mmol, 222 µL), phenylacetylene (3,5 mmol, 384 µL) were added to a 3-neck round-bottom flask under argon counterflow. This was immediately followed by the addition of dry DMF (6 mL). Finally, the base triethylamine (6 mmol, 831 µL) was added, and the flask containing the reaction mixture was attached without delay to the steel tubing coming from the electrochemical cell (155 mA, corresponding to 15% CO content). Upon equipping the reaction flask with a condenser and ensuring a constant gas flow, the reaction vessel was lowered into a pre-heated oil bath at 95 °C and maintained at this temperature for 24 h. A stirring speed of 200 rpm was employed. The reaction mixture was diluted with 10 mL ethyl acetate and transferred to a separating funnel. The reaction mixture was swirled to ensure that the product is well distributed in ethyl acetate/DMF. Following this, the organic phases were washed with 5-6 mL of water 4 to 5 times till no more emulsion resulting from DMF in the aqueous phase was seen. The aqueous phase was then re-extracted to check if any product remained behind. The organic phase was dried with $MgSO_4$ and filtered. Rotary evaporation of the solvent gave the crude product, which was bound to silica and subjected to column chromatography with PE/Et_2O 95:5. The final pure product was isolated as a yellow oil (194 mg, 47%).

17. General Procedure for the Continuous Carbonylative Sonogashira Couplings

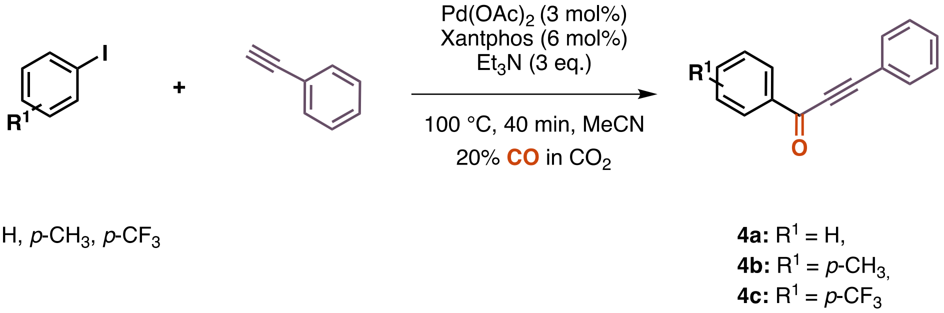

Figure S16. Synthesis of compounds **4a-c**.

A 30-mL vial with septum was charged with the corresponding aryl iodide (1 eq., 1 mmol), phenylacetylene (1.75 eq., 1.75 mmol, 192 µL), $Pd(OAc)_2$ (3 mol%, 0.03 mmol, 6.74 mg), Xantphos (6 mol%, 0.06 mmol, 34.7 mg) and triethylamine (3 eq., 3 mmol, 418 µL), and the reactants were dissolved in 10 mL acetonitrile. The solvent bottle was charged with MeCN. The reactor was heated up to the desired temperature (100 °C). Pump A was used as a back-pressure regulator (BPR, 3 bar). Pump B was connected to the vial with the reaction mixture; pump C was connected to the gas tube, where the pre-mixed gas mixture was introduced. The gases were supplied from the respective cylinders and were pre-mixed with the aid of two mass flow controllers (CO: 0.9 mL/min; and CO_2: 3.6 mL/min). Alternatively, pump C was connected to the solid-oxide electrochemical cell (207 mA, corresponding to 20% CO content), employing a flow rate of 8.0 mL/min. The tubing was primed with the reagent mixture and acetonitrile, respectively. The reactor (10-mL coil reactor) was initially rinsed by a CO/CO_2/MeCN flow for several minutes. Then, the reaction mixture was supplied to the reactor (pump B: 0.25 mL/min; pump C: 3.6 mL/min (or 8.0 mL/min if the electrochemical cell was employed)). After the whole volume of the reaction mixture was pumped through the reactor, the vial was rinsed with pure MeCN, and the residue was pumped through the reactor. The product was collected for 50 minutes. Rotary evaporation of the solvent gave the crude product, which was bound to silica and subjected to column chromatography.

1,3-diphenylprop-2-yn-1-one (4a)[19]

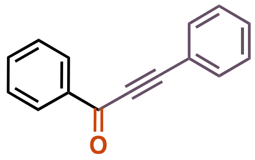

Flash column chromatography (petroleum ether/Et_2O 20:1, R_f=0.50) afforded the product as yellow oil (159 mg, 77%). **^{1}H NMR** (400 MHz, $CDCl_3$) δ 8.27 – 8.20 (m, 2H), 7.73 – 7.67 (m, 2H), 7.67 – 7.61 (m, 1H), 7.56 – 7.46 (m, 3H), 7.46 – 7.39 (m, 2H). **^{13}C NMR** (101 MHz, CDCl3) δ 178.18, 137.05, 134.27, 133.23, 130.94, 129.73, 128.84, 128.78., 120.30, 93.25, 87.04.

3-phenyl-1-(*p*-tolyl)prop-2-yn-1-one (4b)[20]

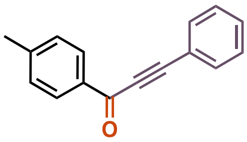

Flash column chromatography (petroleum ether/Et_2O 20:1, R_f=0.54) afforded the product as yellow solid (167 mg, 76%).**^{1}H NMR** (400 MHz, $CDCl_3$) δ 8.15 – 8.09 (m, 2H), 7.71 – 7.65 (m, 2H), 7.55 – 7.38 (m, 3H), 7.36 – 7.28 (m, 2H), 2.45 (s, 3H). **^{13}C NMR** (101 MHz, $CDCl_3$) δ 177.88, 145.38, 134.78, 133.17, 130.81, 129.87, 129.49, 128.81, 120.43, 92.47, 87.12, 21.99.

3-phenyl-1-(4-(trifluoromethyl)phenyl)prop-2-yn-1-one (4c)[21]

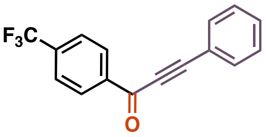

Flash column chromatography (petroleum ether/Et_2O 25:1, R_f=0.41) afforded the product as yellow solid (55 mg, 20%). **^{1}H NMR** (400 MHz, $CDCl_3$) δ 8.33 (dq, J = 7.7, 0.9 Hz, 2H), 7.82 – 7.76 (m, 2H), 7.73 – 7.67 (m, 2H), 7.55 – 7.49 (m, 1H), 7.48 – 7.42 (m, 2H). **^{13}C NMR** (101 MHz, $CDCl_3$) δ 176.88, 139.54, 135.52, 135.19, 133.36, 131.36, 129.96, 128.96, 125.93, 125.89, 125.85, 125.82, 125.05, 122.34, 119.83, 94.64, 86.73. **^{19}F NMR** (376 MHz, $CDCl_3$) δ -63.13.

18. General Procedure for the Continuous Aminocarbonylations

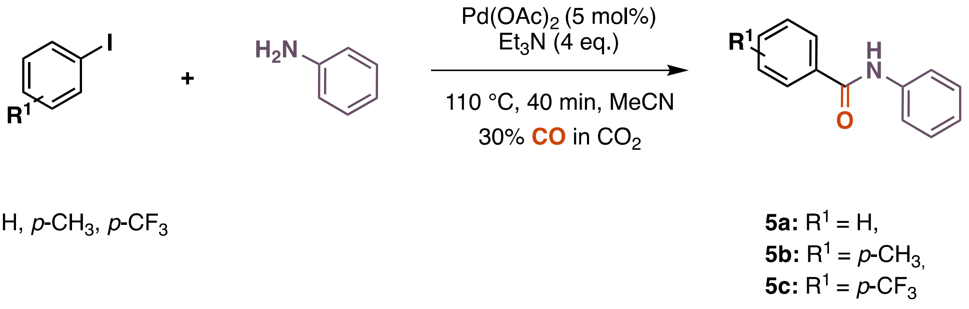

Figure S17. Synthesis of compounds **5a-c**.

A 30-mL vial with septum was charged with the corresponding aryl iodide (1 eq., 1 mmol), aniline (3 eq., 3 mmol), $Pd(OAc)_2$ (5 mol%, 0.05 mmol, 11.2 mg), and triethylamine (4 eq., 4 mmol, 557 µL), and the reactants were dissolved in 10 mL acetonitrile. The solvent bottle was charged with MeCN. The reactor was heated up to the desired temperature (110 °C). Pump A was used as a back-pressure regulator (BPR, 4 bar). Pump B was connected to the vial with the reaction mixture; pump C was connected to the gas tube, where the pre-mixed gas mixture was introduced. The gases were supplied from the respective cylinders and were pre-mixed with the aid of two mass flow controllers (CO: 1.55 mL/min; and CO_2: 3.6 mL/min). The tubings were primed with the reagent mixture and acetonitrile, respectively. The reactor (10-mL coil reactor) was initially rinsed by a CO/CO_2/MeCN flow for several minutes. Then, the reaction mixture was supplied to the reactor (pump B: 0.25 mL/min; pump C: 3.6 mL/min). After the whole volume of the reaction mixture was pumped through the reactor, the vial was rinsed with pure MeCN, and the residue was pumped through the reactor. The product was collected for 50 minutes. After evaporation of the solvent, the residue was taken up in 20 mL DCM and was washed thrice with 1 N HCl solution. The organic phases were collected and dried over anhydrous Na_2SO_4. After removing the solvent, the crude product was bound to silica and subjected to column chromatography.

N-phenylbenzamide (5a)[22]

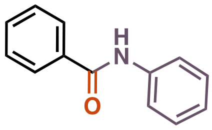

Flash column chromatography (petroleum ether/EtOAc 7:1, R_f=0.42) afforded the product as yellowish-white, crystalline solid (139 mg, 71%). **^{1}H NMR** (400 MHz, DMSO) δ 10.25 (s, 1H), 7.98 – 7.93 (m, 2H), 7.80 – 7.75 (m, 2H), 7.62 – 7.50 (m, 3H), 7.40 – 7.30 (m, 2H),

7.10 (tt, J = 7.3, 1.2 Hz, 1H). **^{13}C NMR** (101 MHz, DMSO) δ 165.61, 139.18, 135.01, 131.57, 128.63, 128.41, 127.67, 123.70, 120.40.

4-methyl-N-phenylbenzamide (5b)[23]

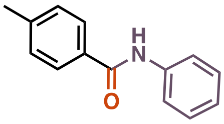

Flash column chromatography (petroleum ether/EtOAc 3:1, R_f=0.47) afforded the product as yellowish-white, crystalline solid (134 mg, 63%). **^{1}H NMR** (400 MHz, DMSO) δ 10.14 (s, 1H), 7.91 – 7.82 (m, 2H), 7.81 – 7.71 (m, 2H), 7.33 (t, *J* = 7.9 Hz, 4H), 7.08 (tt, *J* = 7.2, 1.2 Hz, 1H), 2.38 (s, 3H). **^{13}C NMR** (101 MHz, DMSO) δ 165.34, 141.53, 139.24, 132.08, 128.89, 128.56, 127.68, 123.53, 120.34, 21.00.

N-phenyl-4-(trifluoromethyl)benzamide (5c)[23]

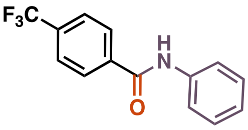

Flash column chromatography (petroleum ether/EtOAc 7:1, R_f=0.44) afforded the product as yellowish, crystalline solid (105 mg, 40%). **^{1}H NMR** (400 MHz, DMSO) δ 10.46 (s, 1H), 8.14 (d, *J* = 8.1 Hz, 2H), 7.91 (d, *J* = 8.2 Hz, 2H), 7.78 (d, *J* = 8.0 Hz, 2H), 7.41 – 7.29 (m, 2H), 7.12 (td, *J* = 7.3, 1.2 Hz, 1H). **^{13}C NMR** (101 MHz, DMSO) δ 164.39, 138.82, 131.51, 131.19, 128.67, 128.59, 125.39, 125.36, 124.02, 120.45. **^{19}F NMR** (376 MHz, DMSO) δ -61.34.

19. NMR Spectra of 1a-5c

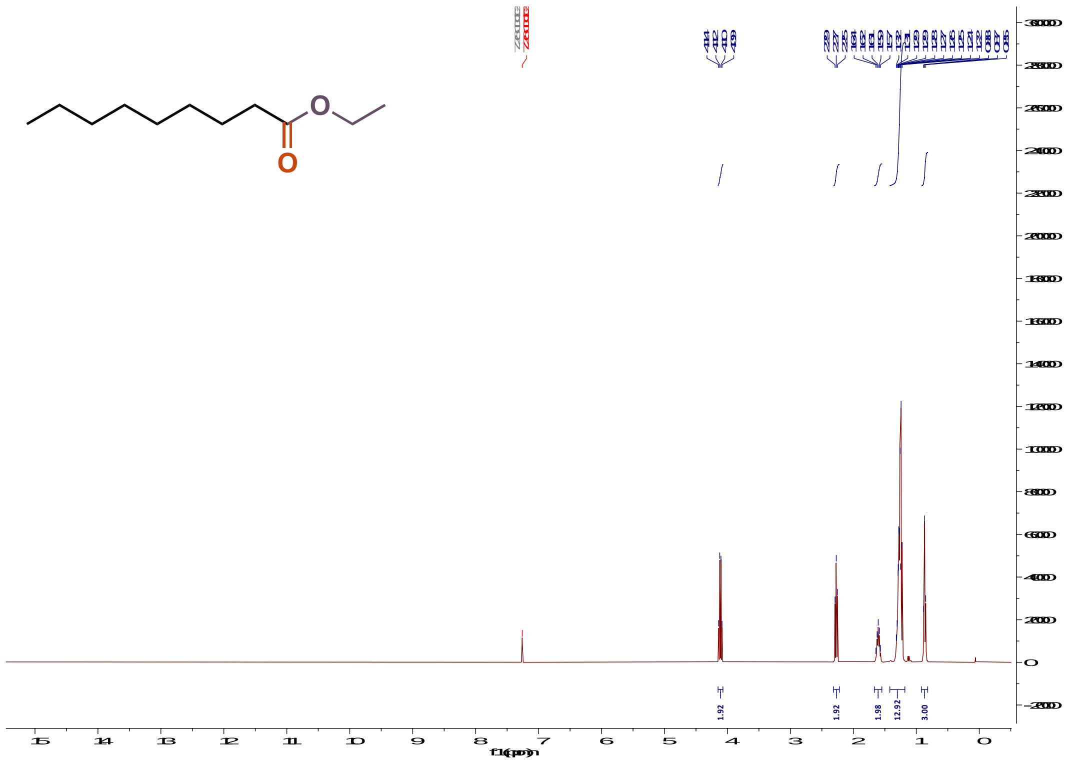

Figure S18. ^{1}H-NMR of **1a**.

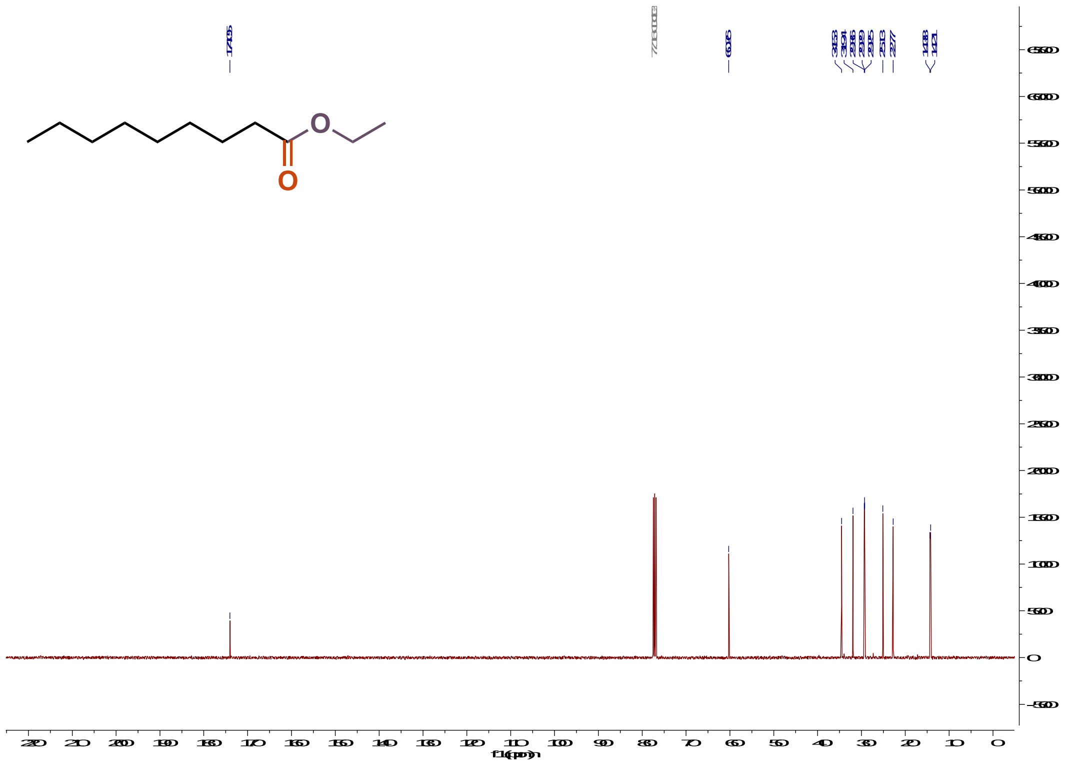

Figure S19. ^{13}C-NMR of **1a**.

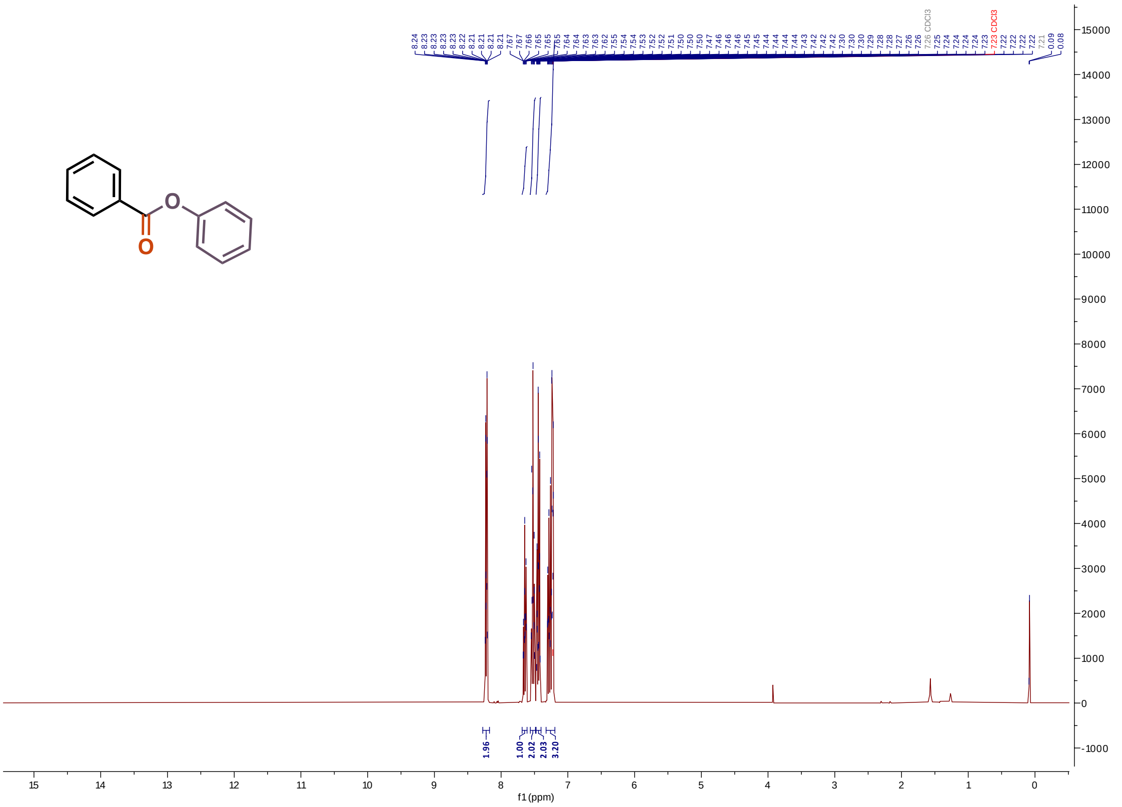

Figure S20. ^{1}H-NMR of **2a**.

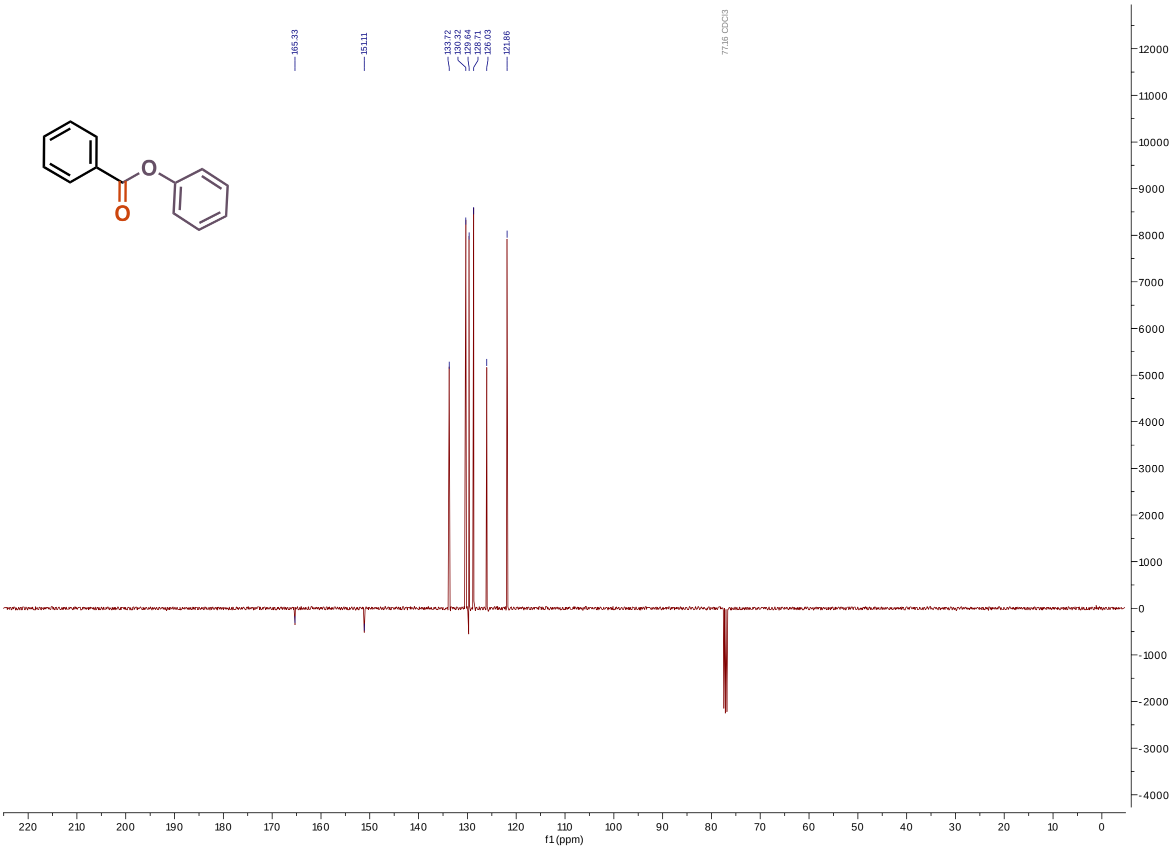

Figure S21. ^{13}C-NMR (APT) of **2a**.

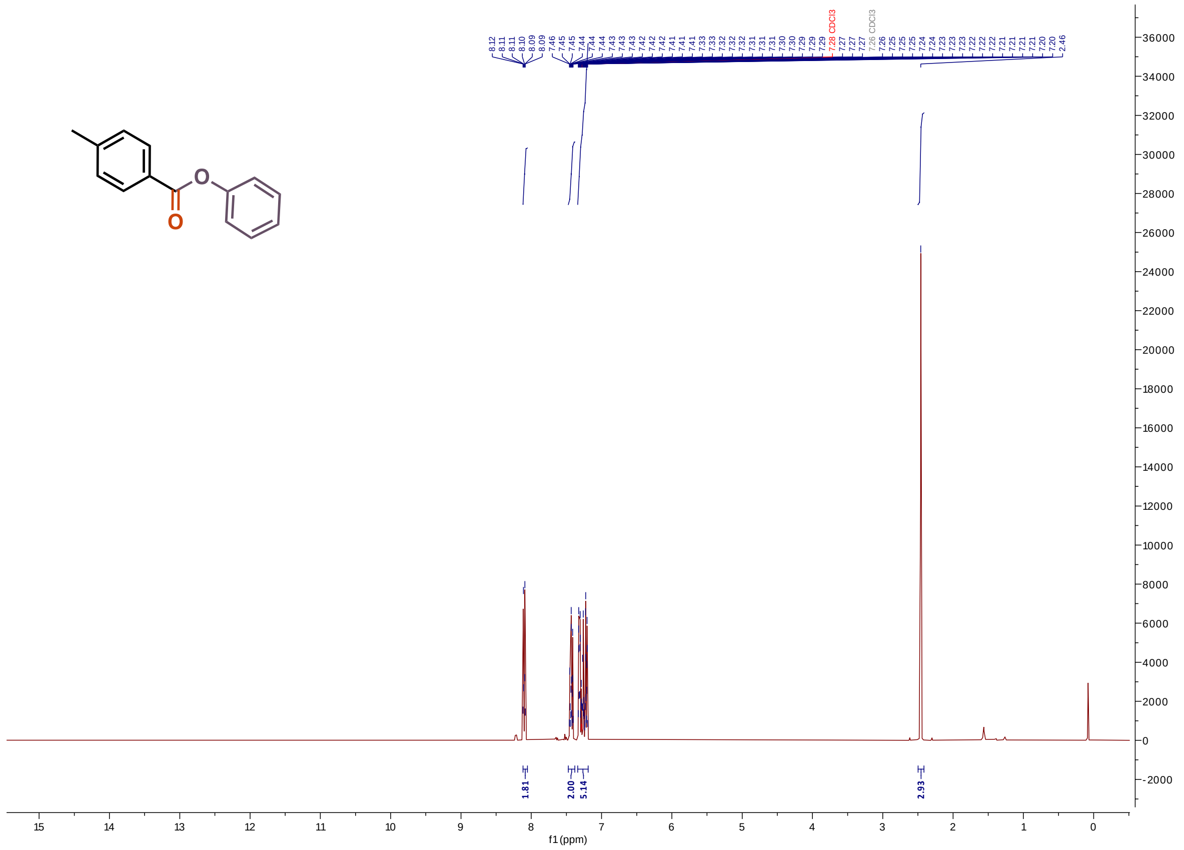

Figure S22. ^{1}H-NMR of **2b**.

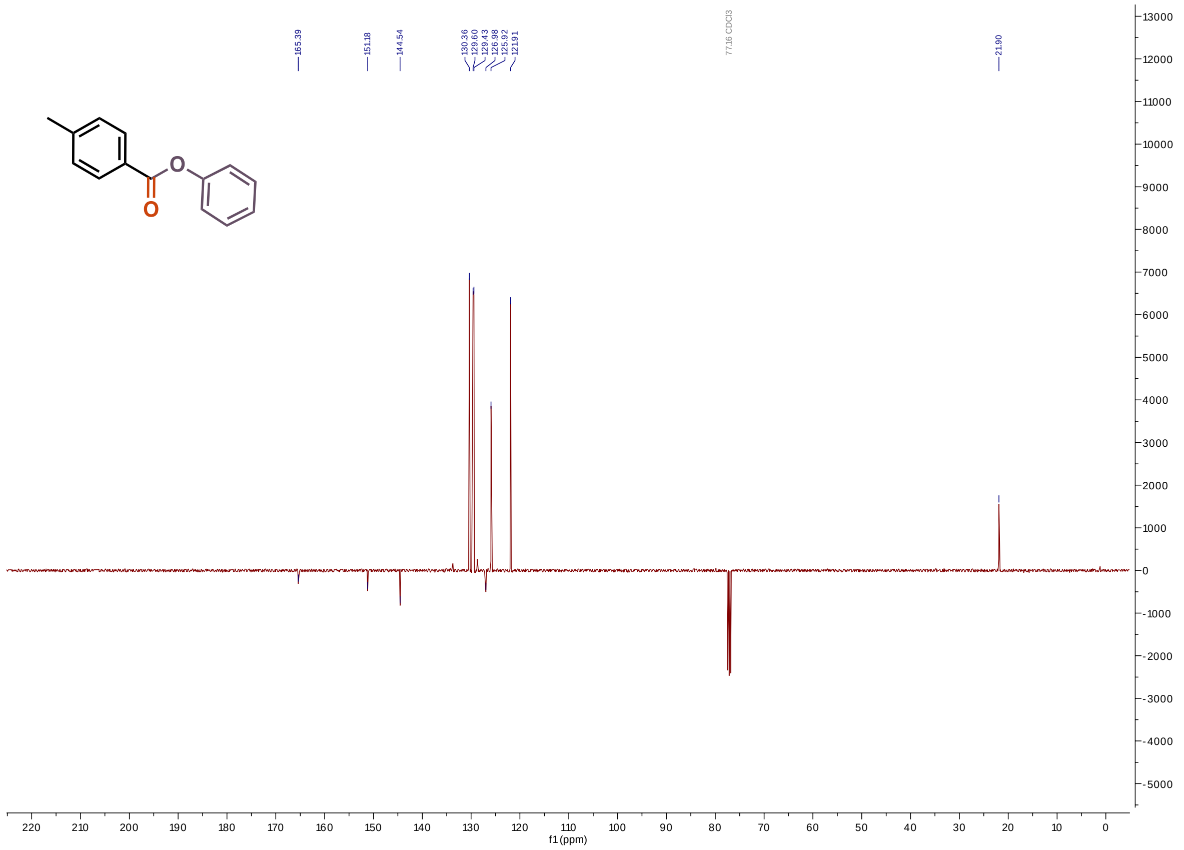

Figure S23. ^{13}C-NMR (APT) of **2b**.

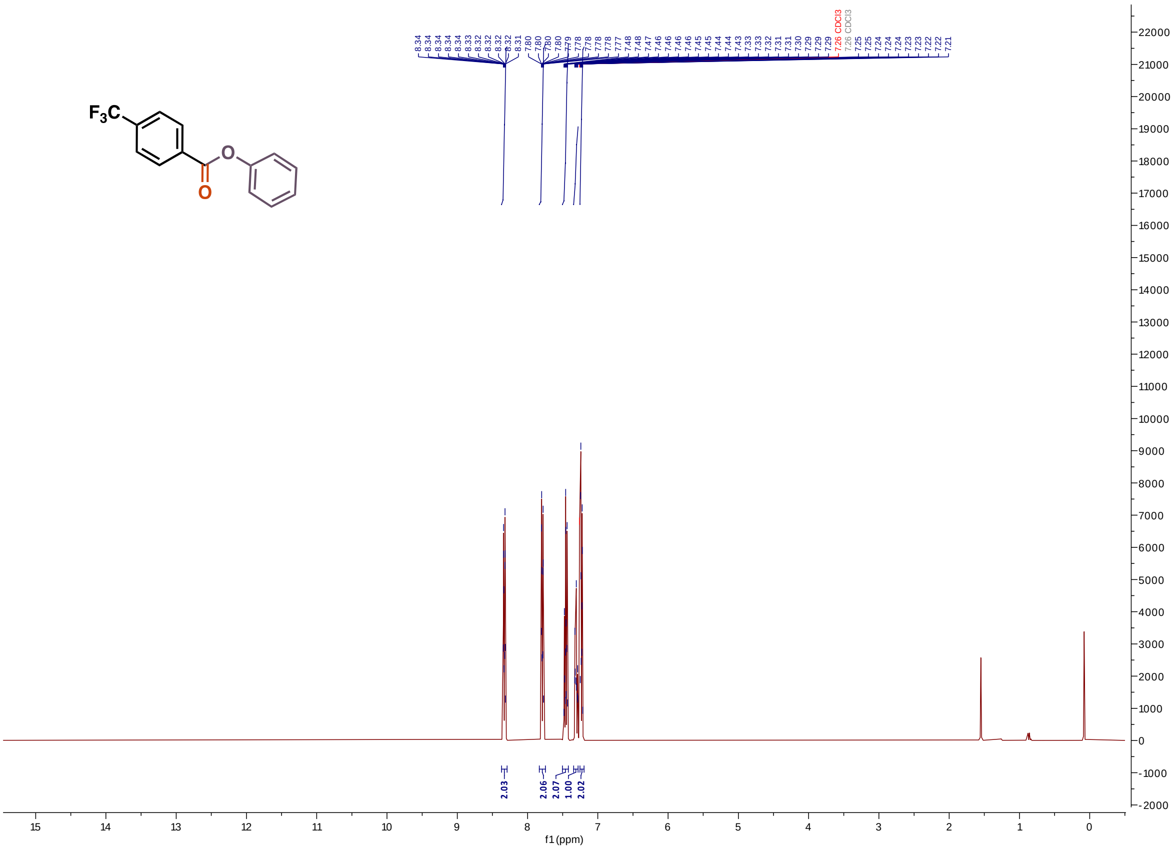

Figure S24. ^{1}H-NMR of **2c**.

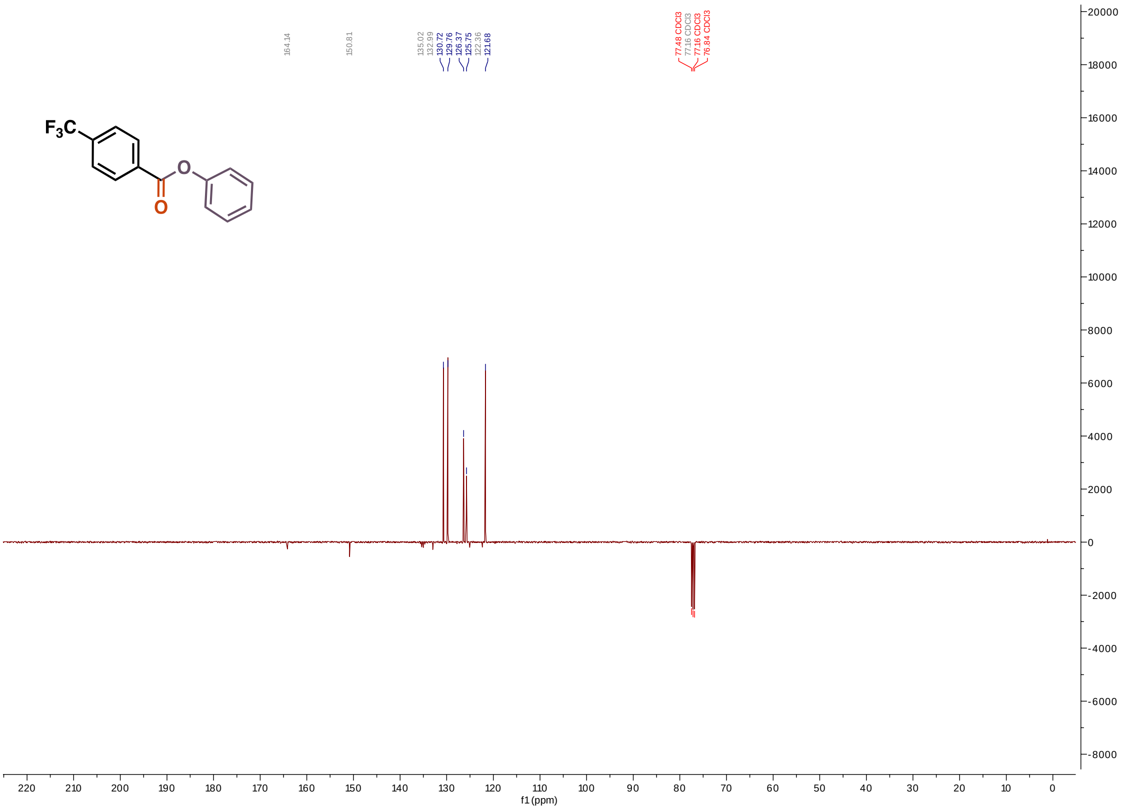

Figure S25. ^{13}C-NMR (APT) of **2c**.

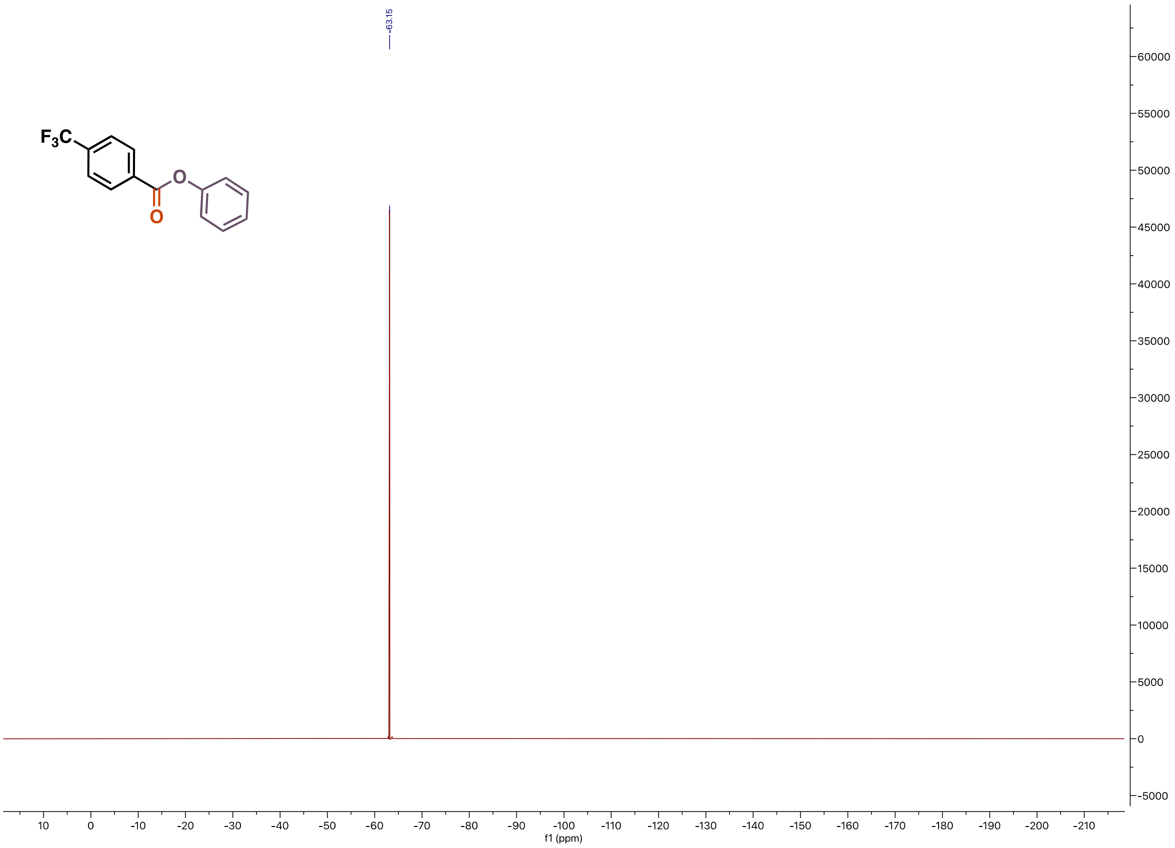

Figure S26. ^{19}F-NMR of **2c**.

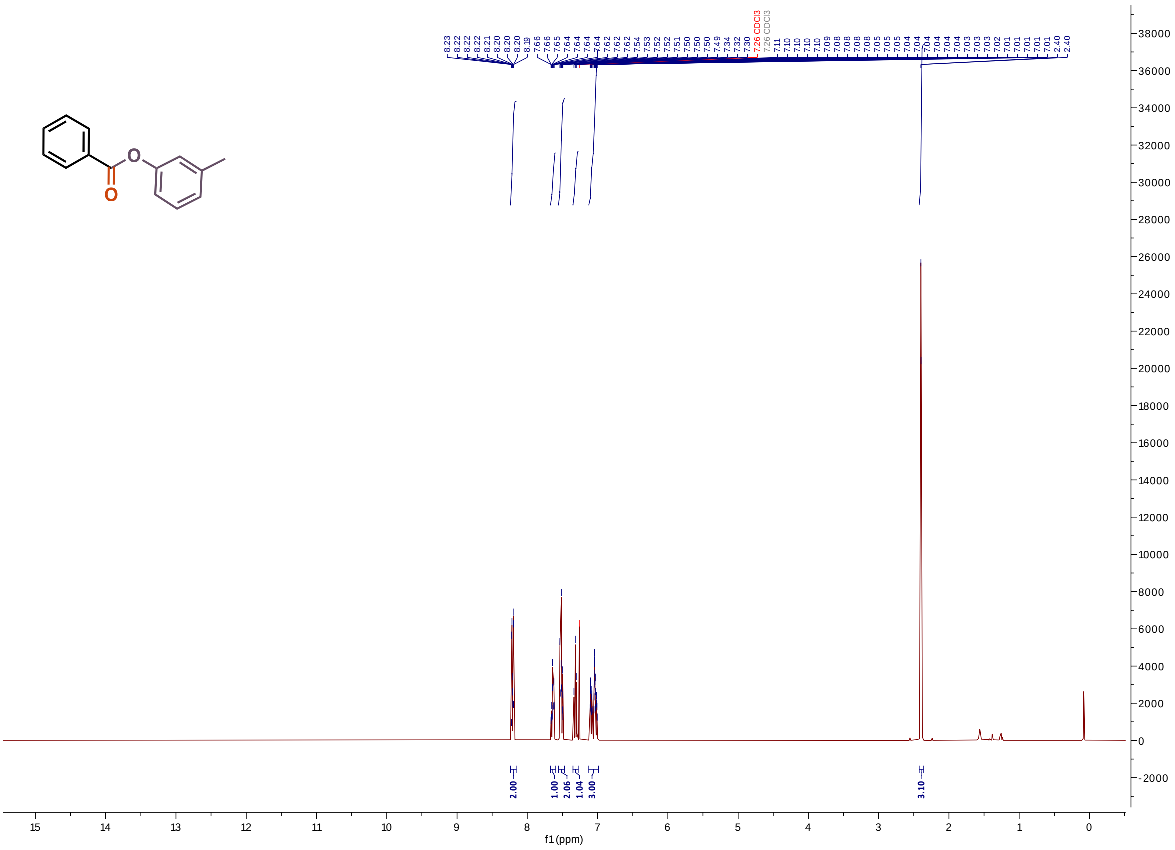

Figure S27. ^{1}H-NMR of **2d**.

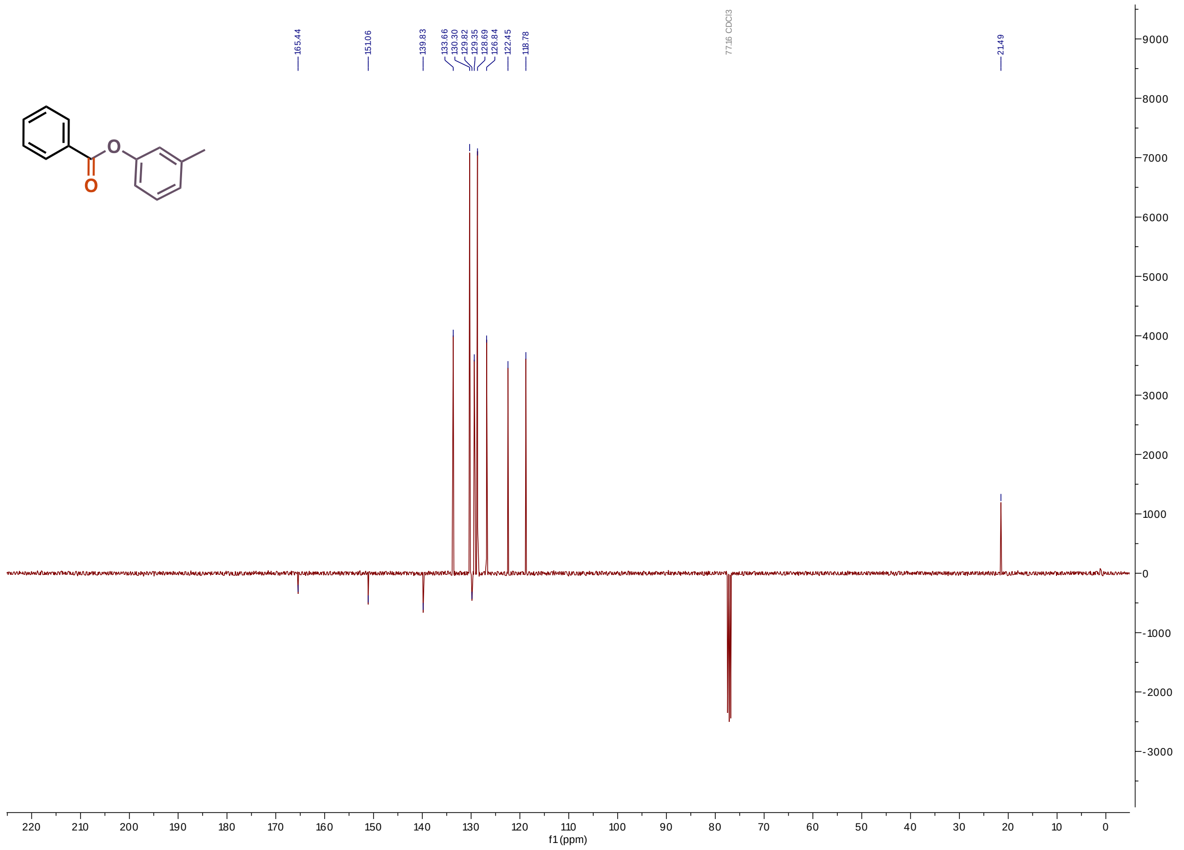

Figure S28. ^{13}C-NMR (APT) of **2d**.

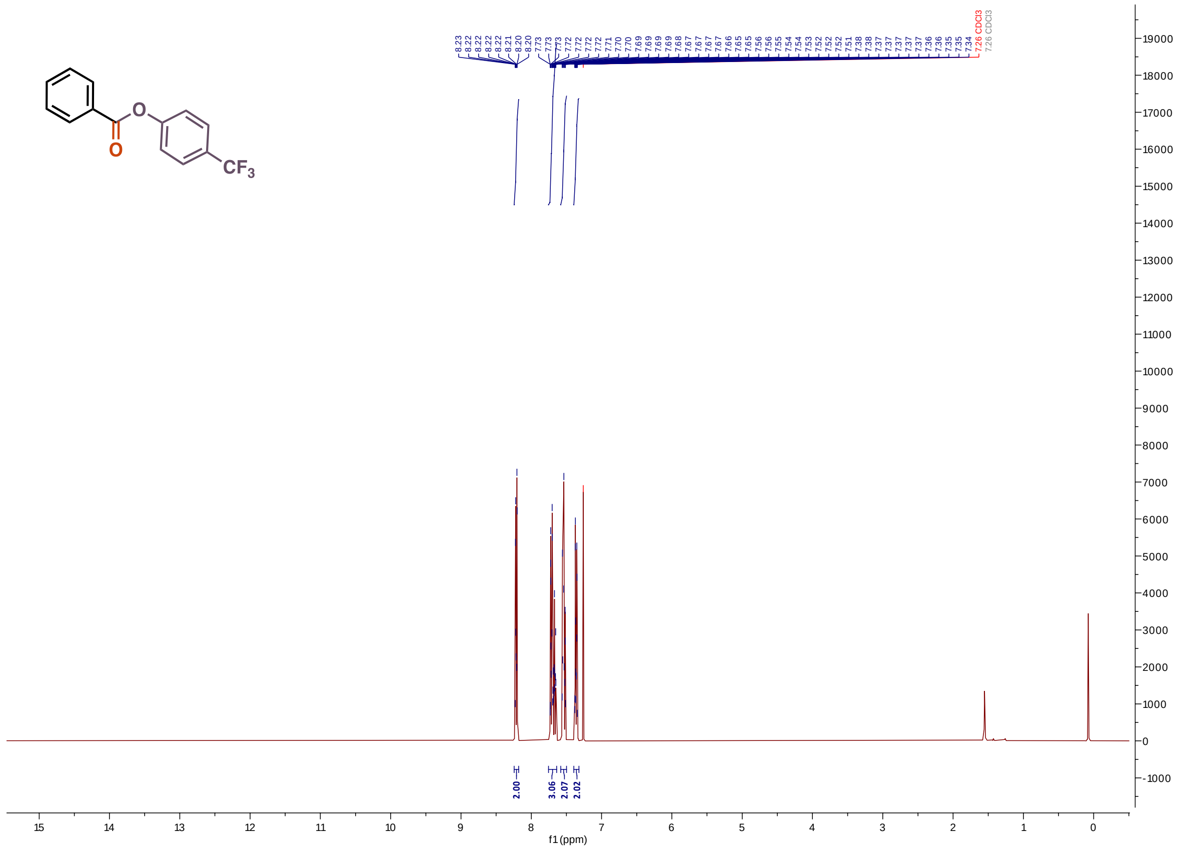

Figure S29. ^{1}H-NMR of **2e**.

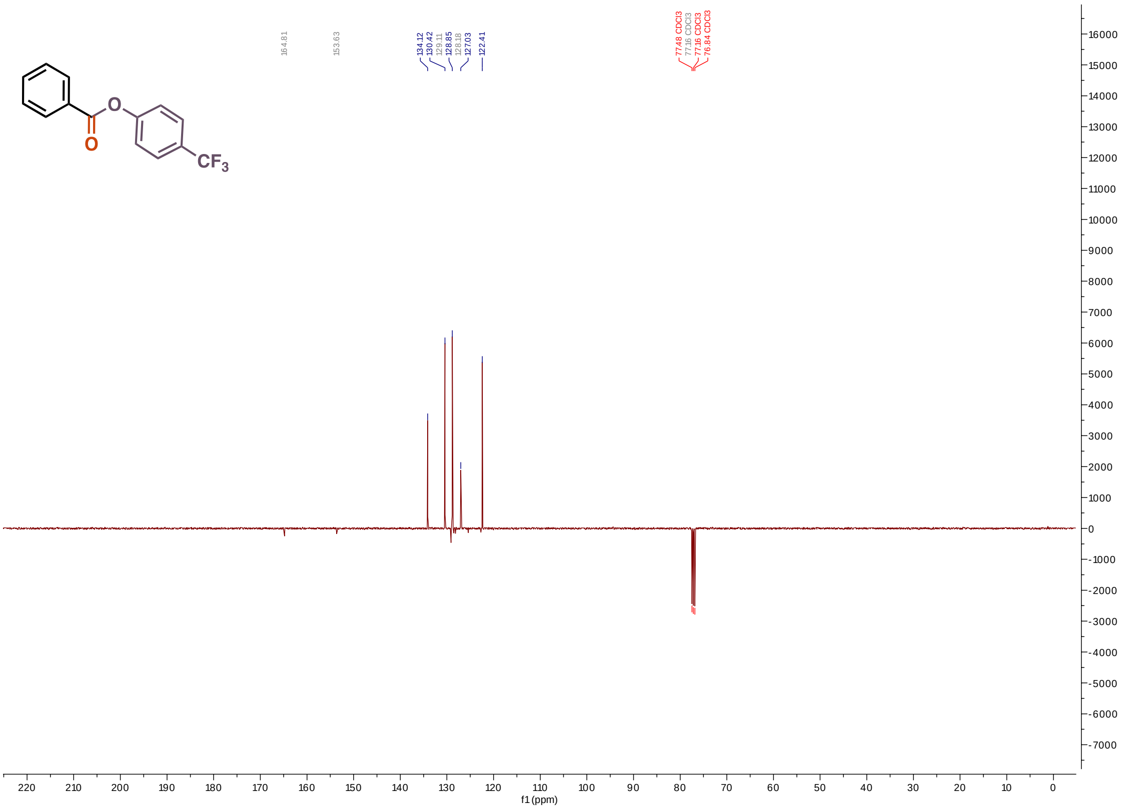

Figure S30. ^{13}C-NMR (APT) of **2e**.

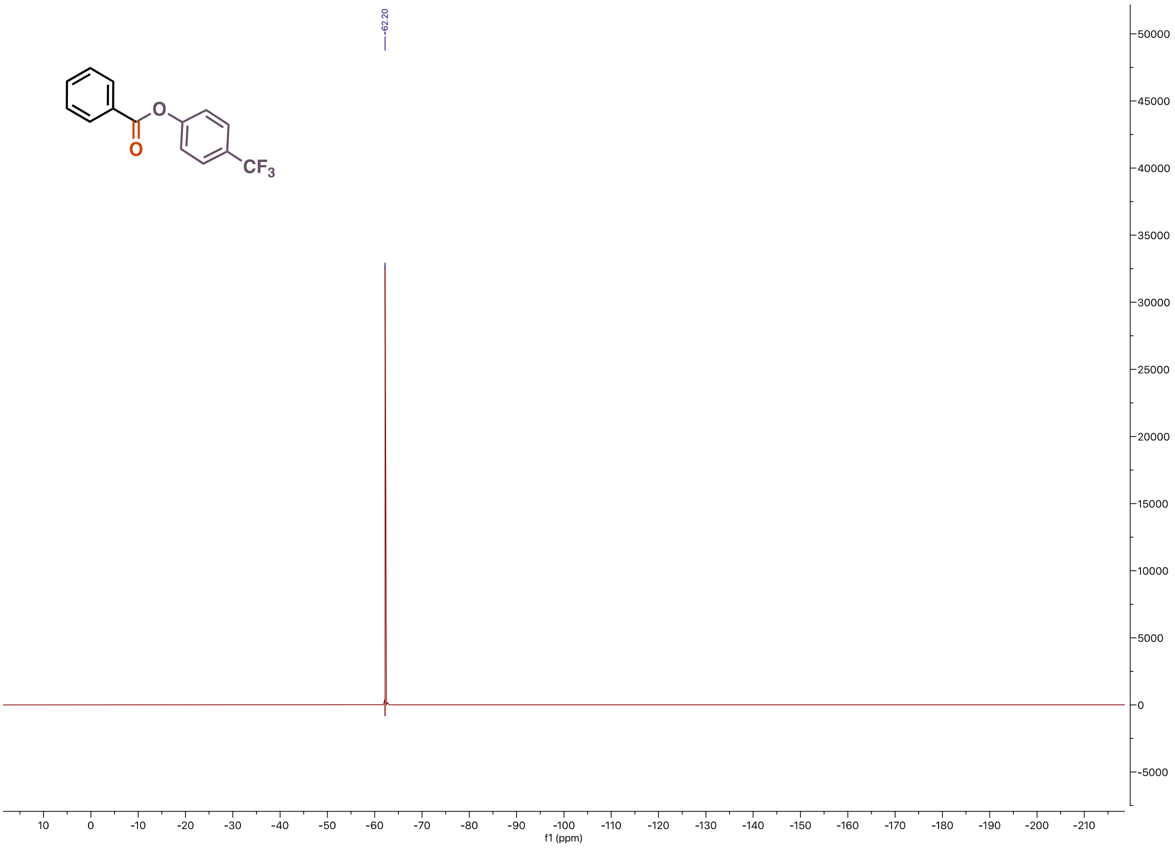

Figure S31. ^{19}F-NMR of **2e**.

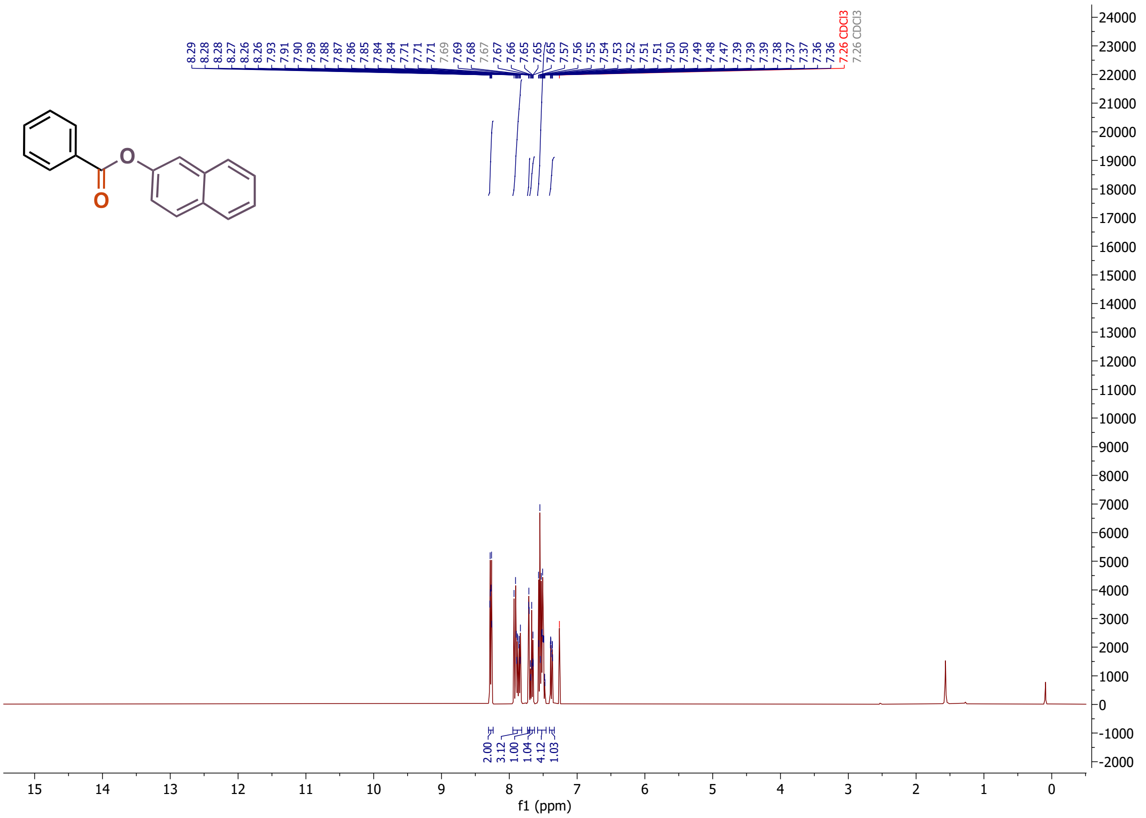

Figure S32. ^{1}H-NMR of **2f**.

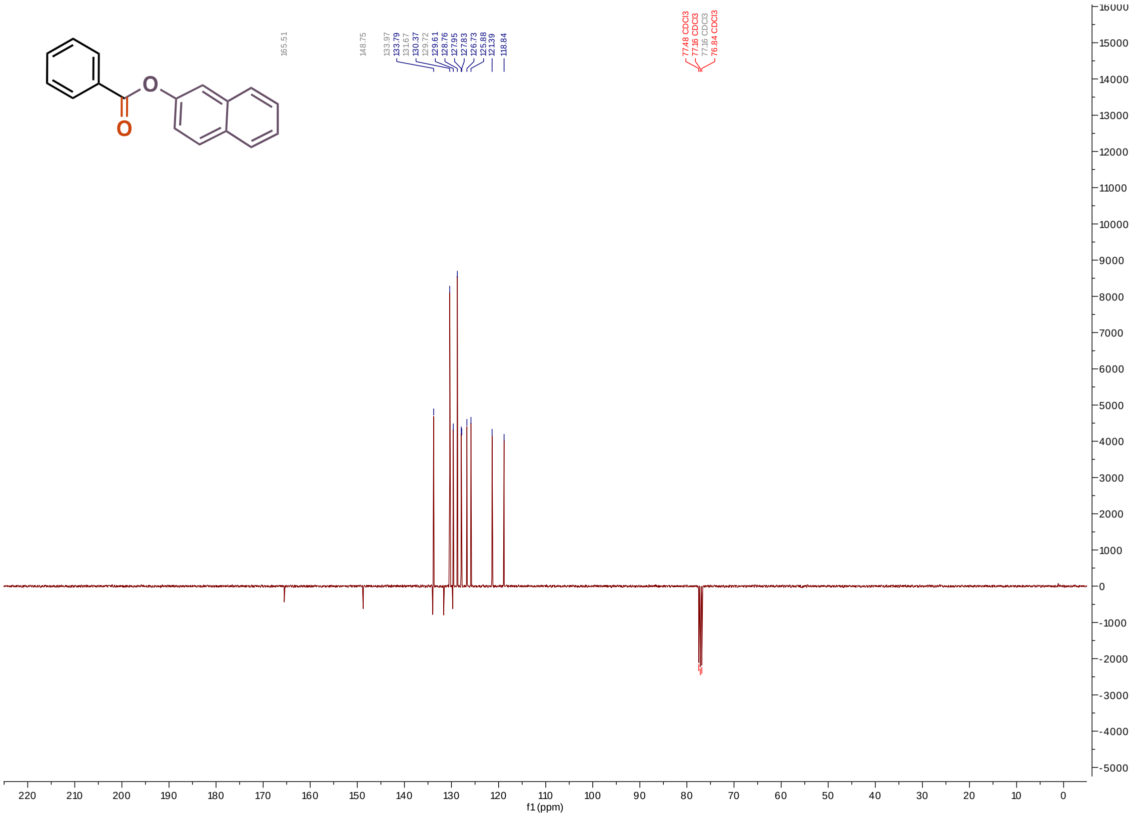

Figure S33. ^{13}C-NMR (APT) of **2f**.

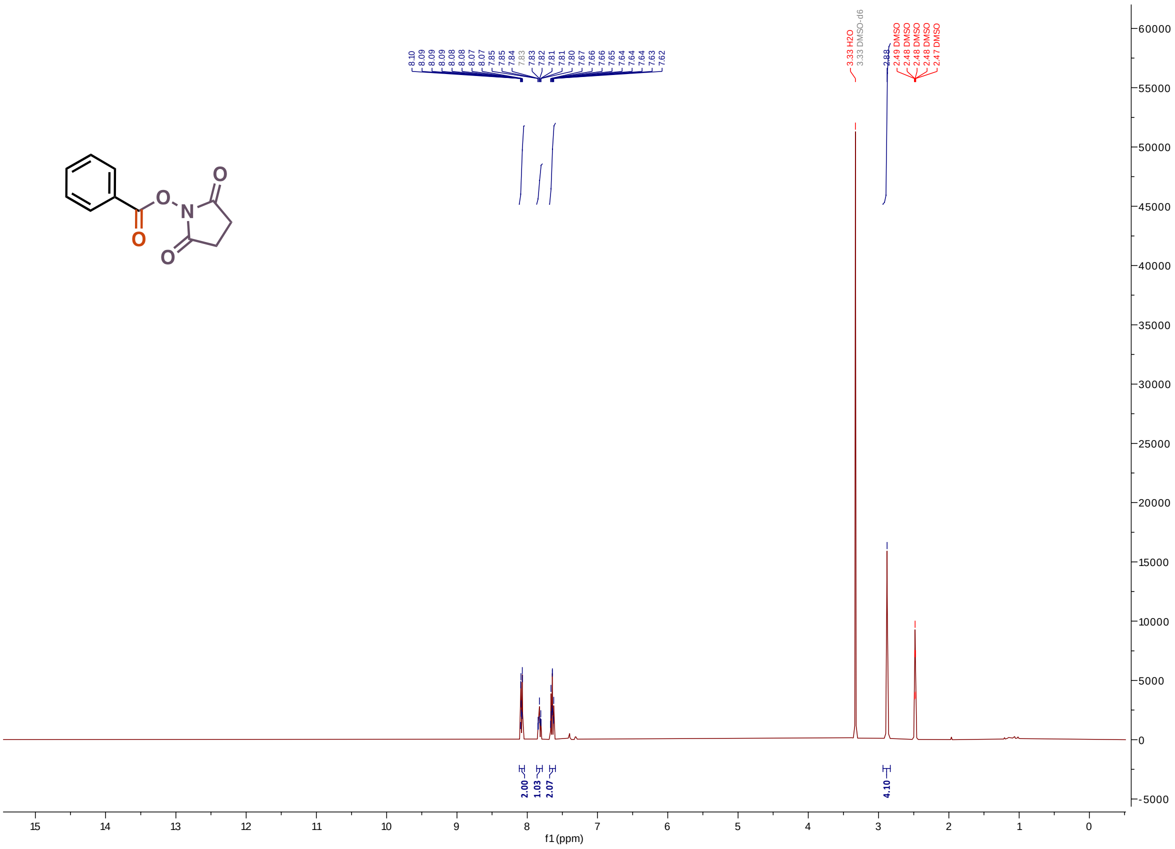

Figure S34. ^{1}H-NMR of **3a**.

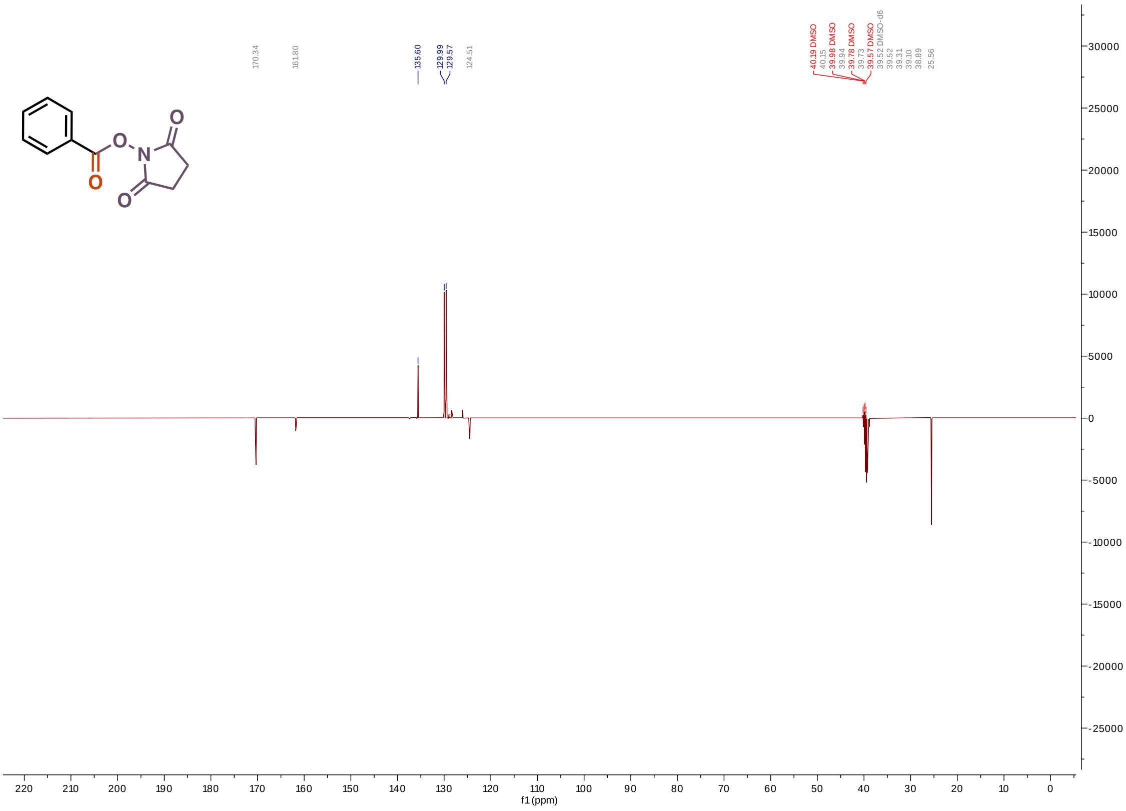

Figure S35. ^{13}C-NMR (APT) of **3a**.

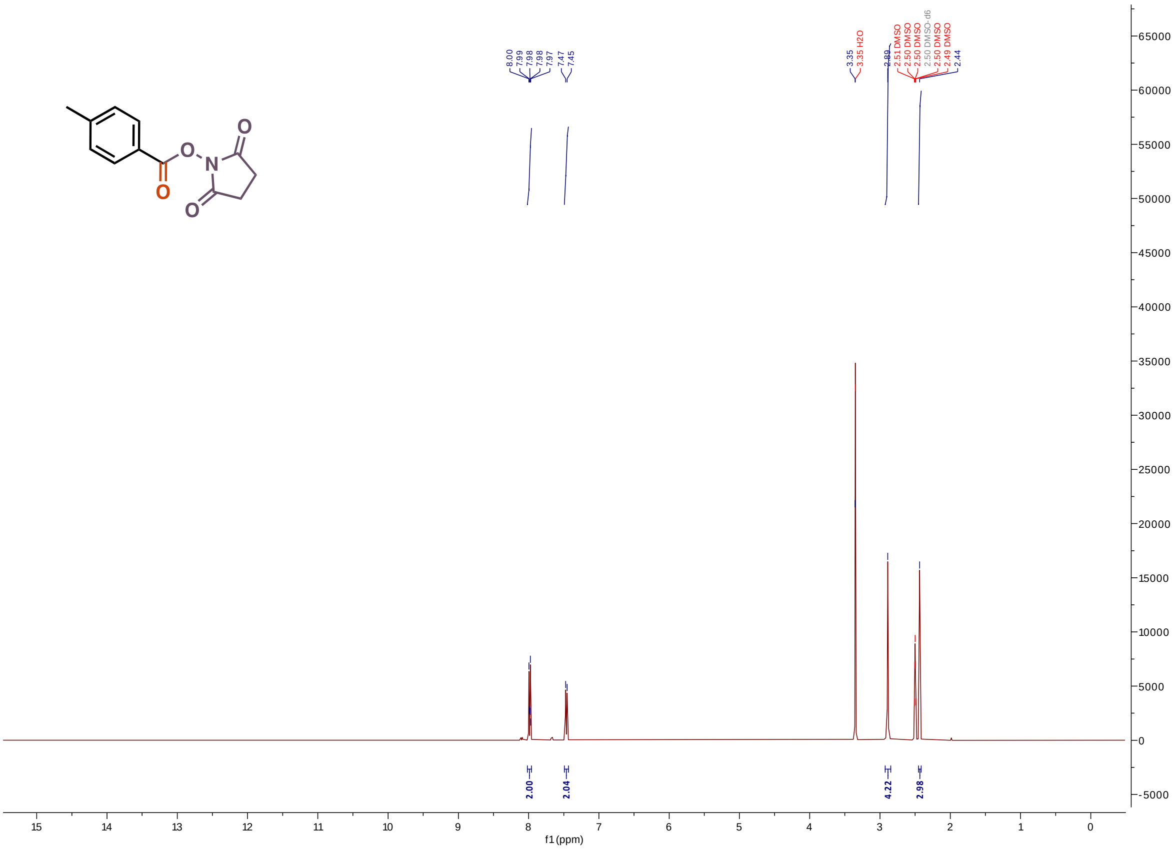

Figure S36. ^{1}H-NMR of **3b**.

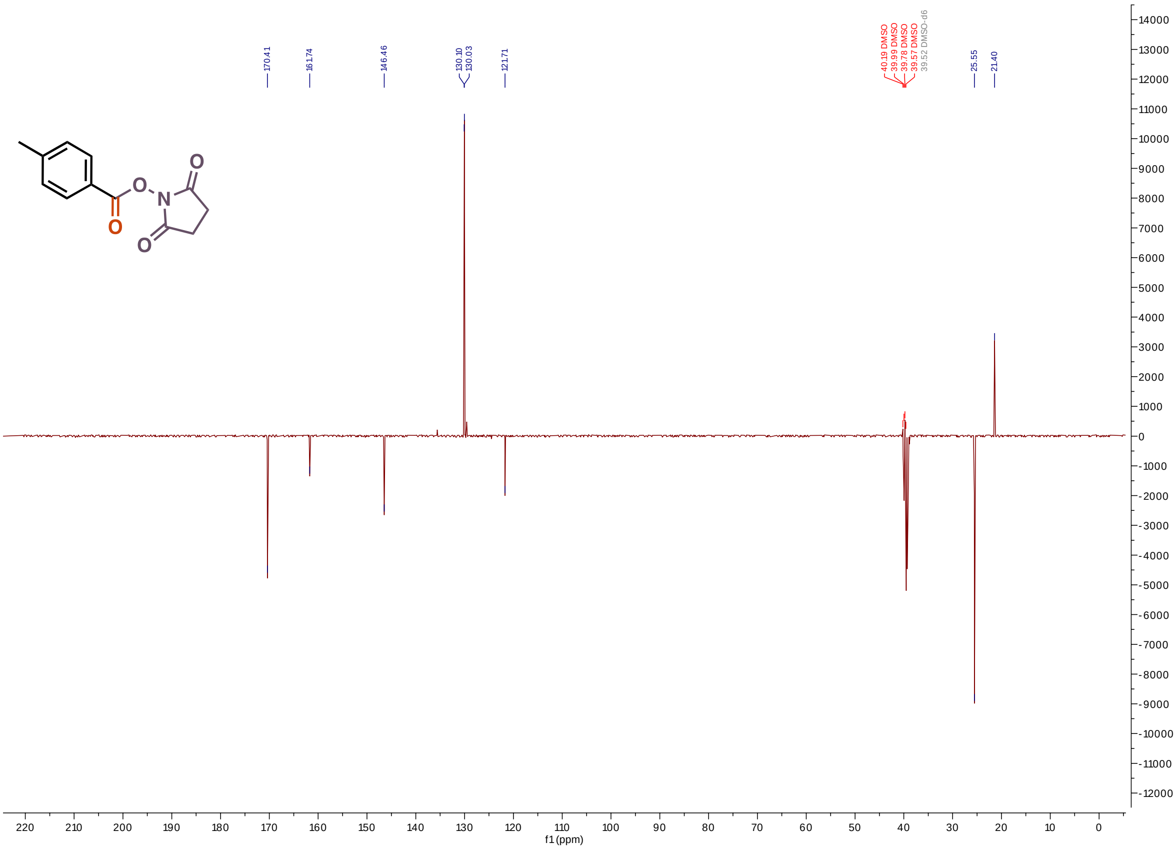

Figure S37. ^{13}C-NMR (APT) of **3b**.

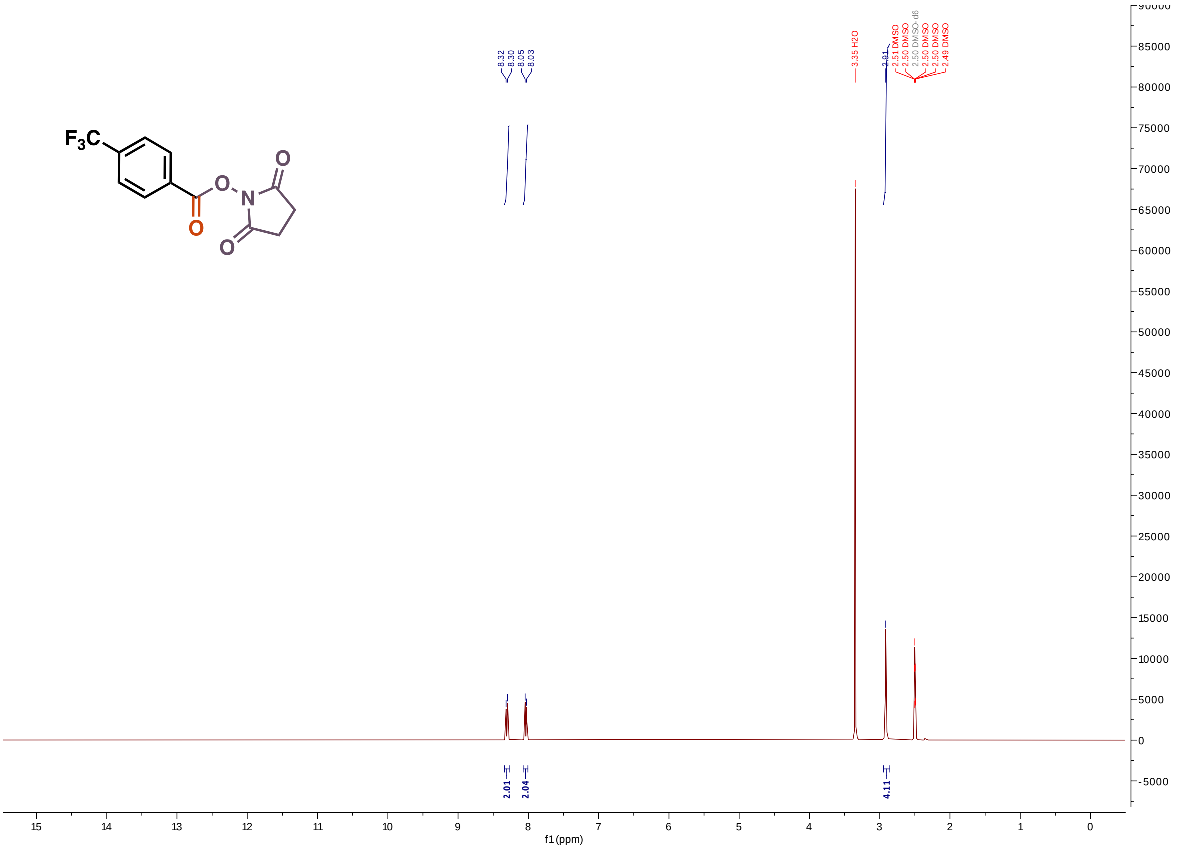

Figure S38. ^{1}H-NMR of **3c**.

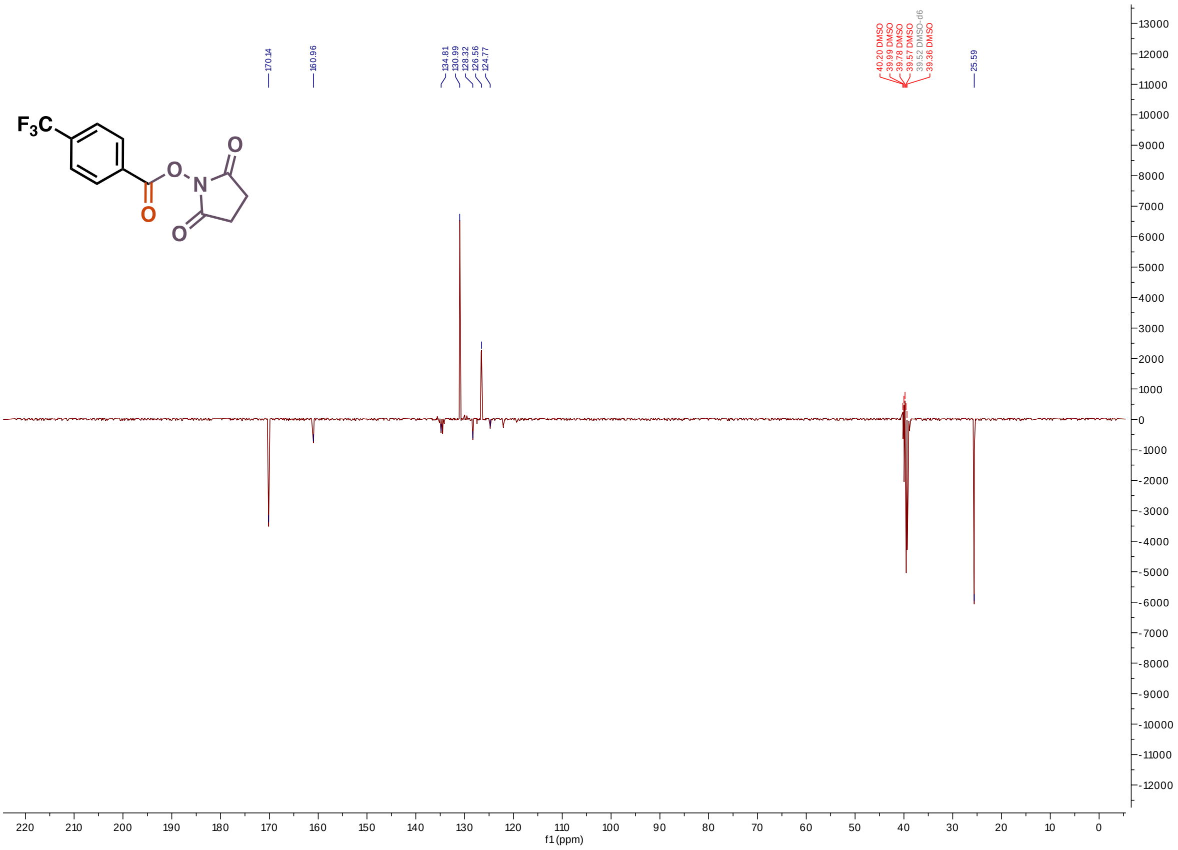

Figure S39. ^{13}C-NMR (APT) of **3c**.

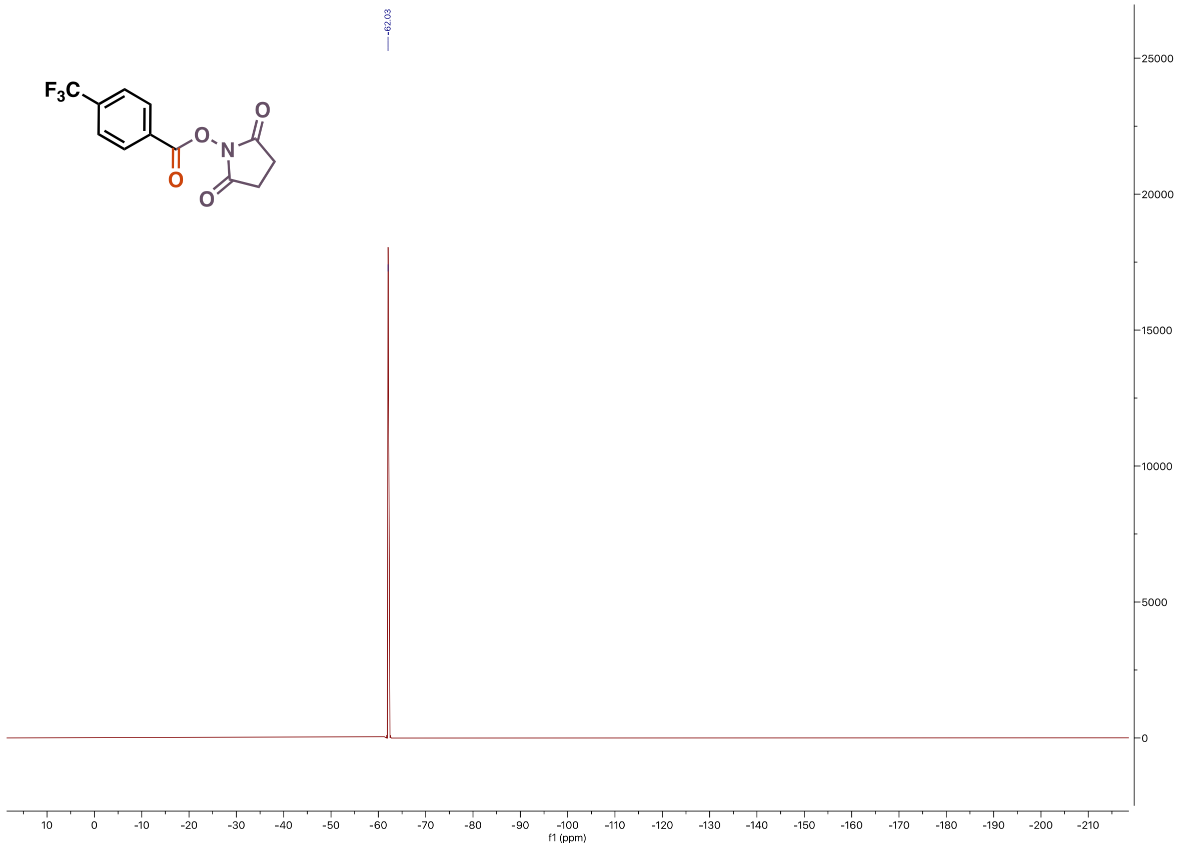

Figure S40. ^{19}F-NMR of **3c**.

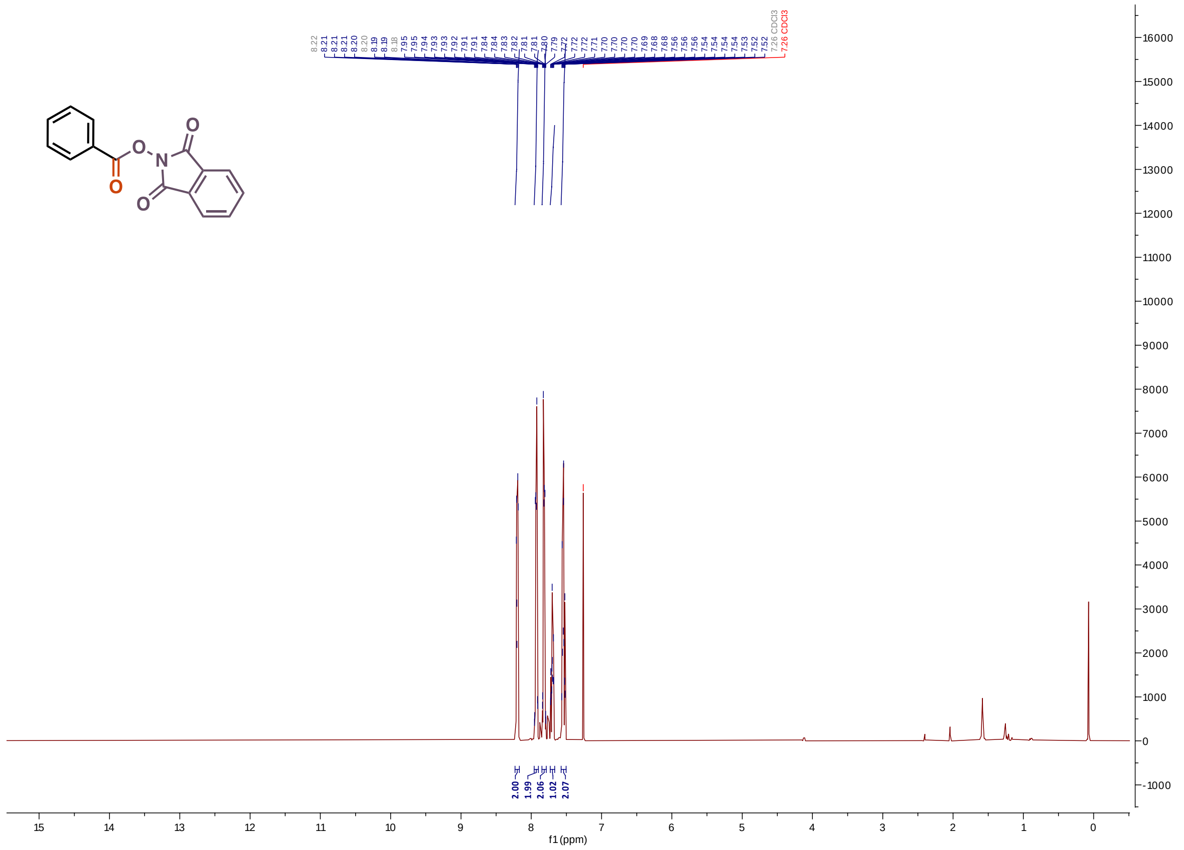

Figure S41. ^{1}H-NMR of **3d**.

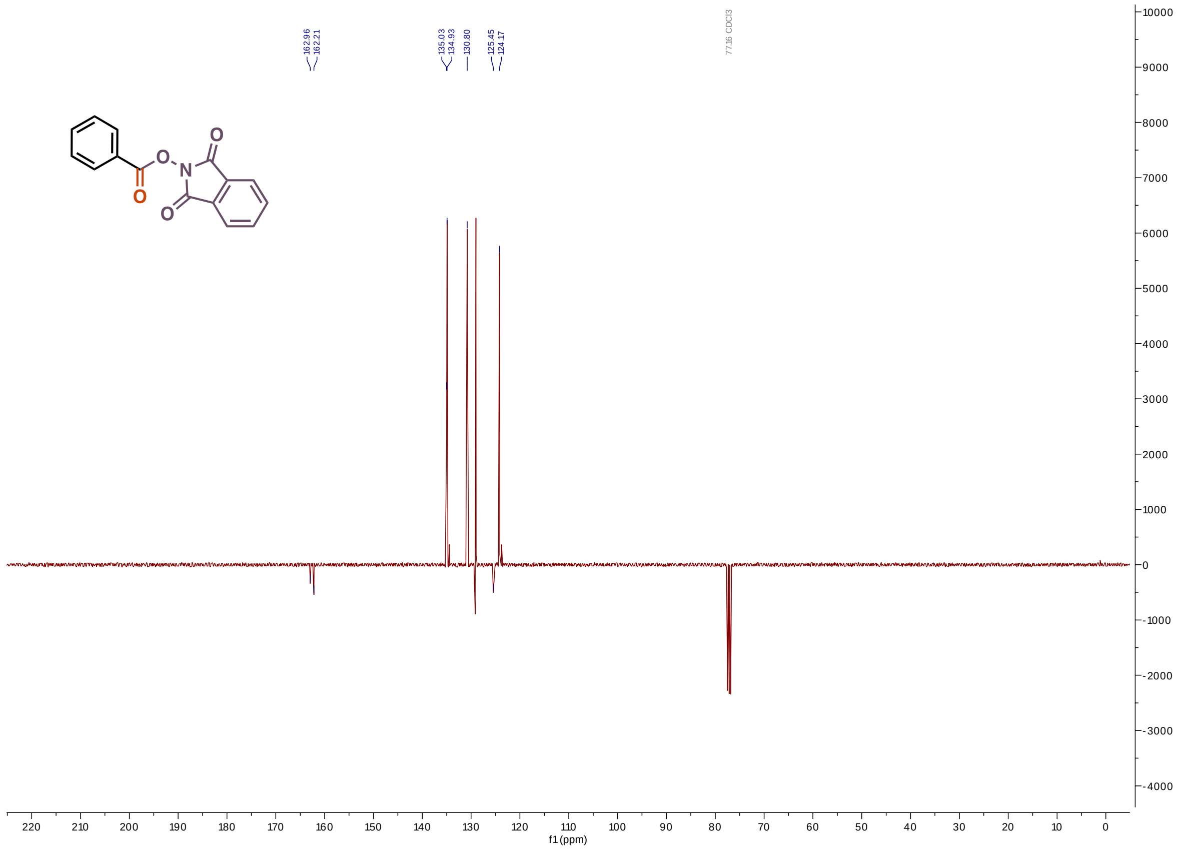

Figure S42. ^{13}C-NMR (APT) of **3d**.

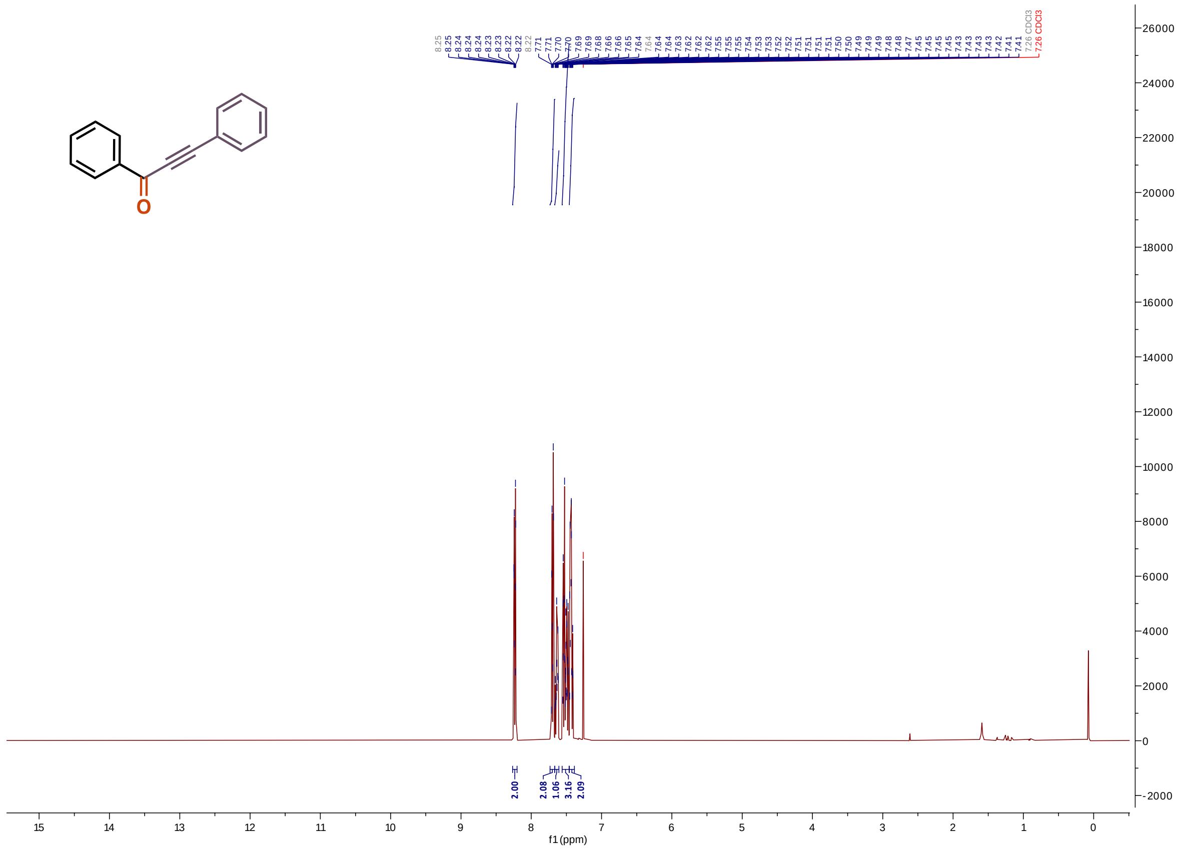

Figure S43. ^{1}H-NMR of **4a**.

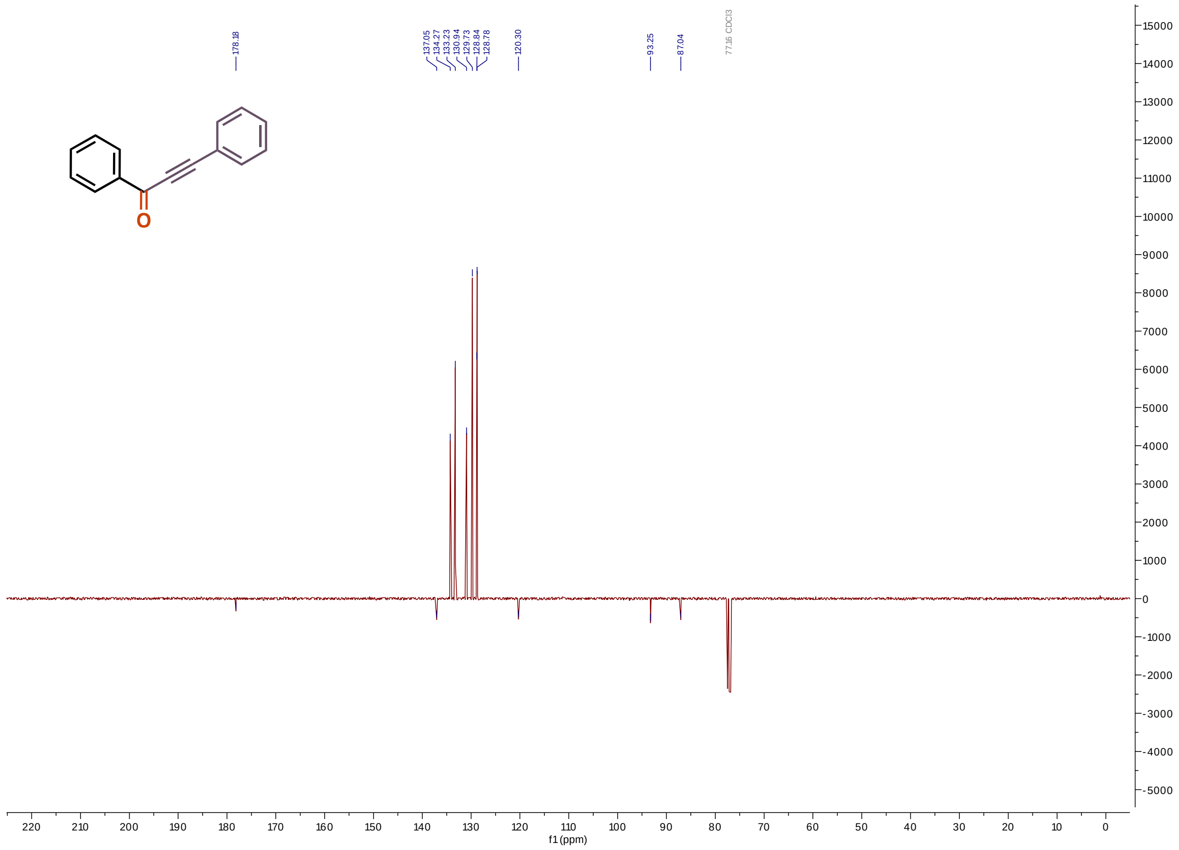

Figure S44. ^{13}C-NMR (APT) of **4a**.

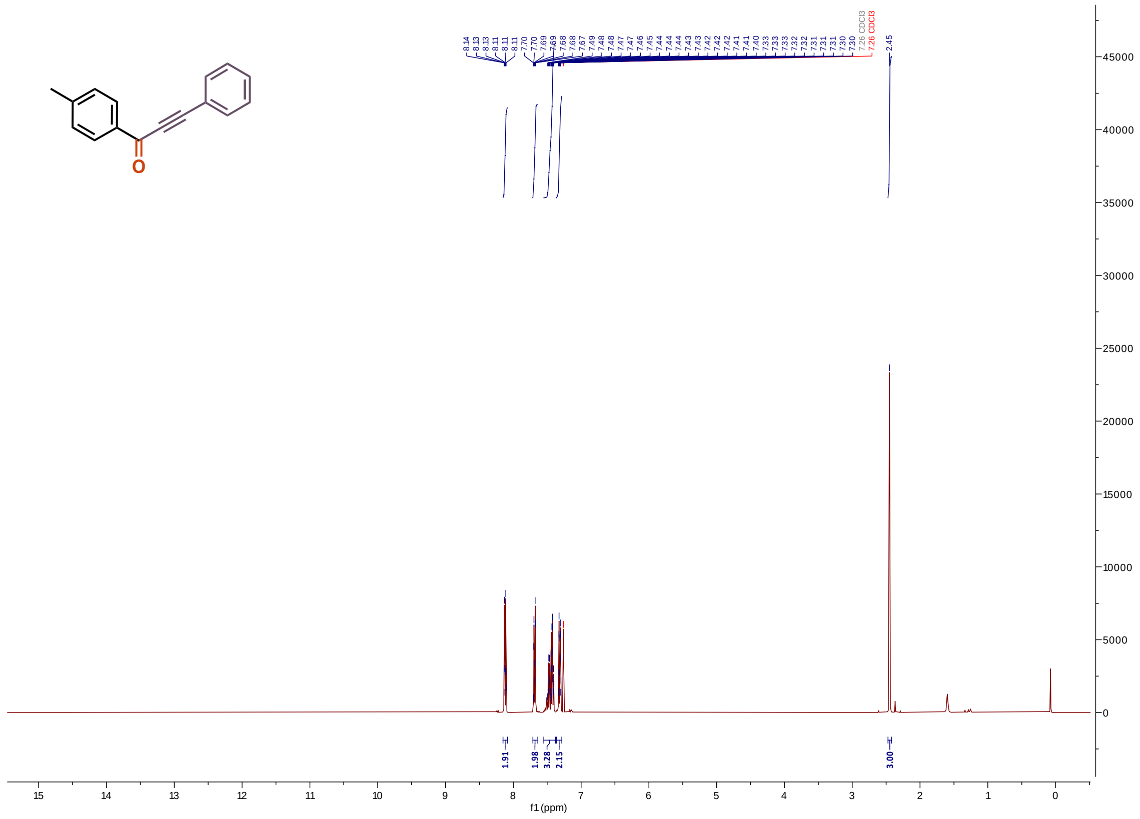

Figure S45. ^{1}H-NMR of **4b**.

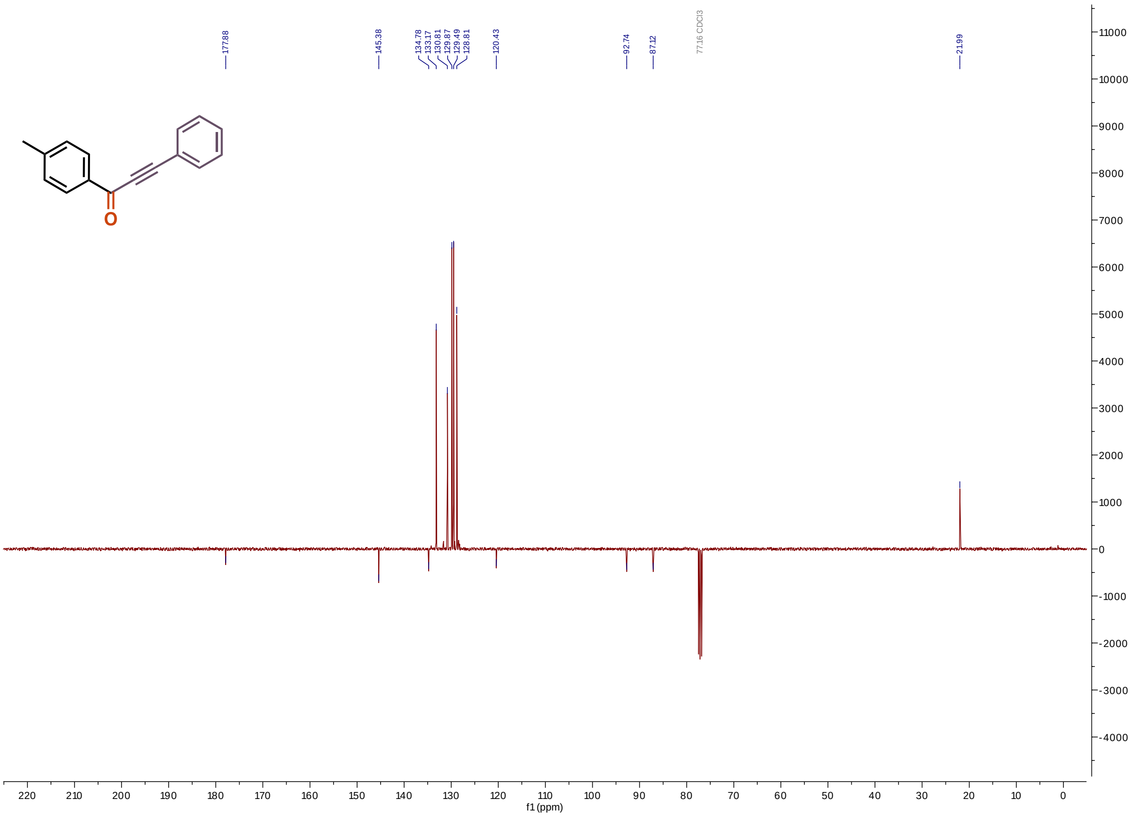

Figure S46. ^{13}C-NMR (APT) of **4b**.

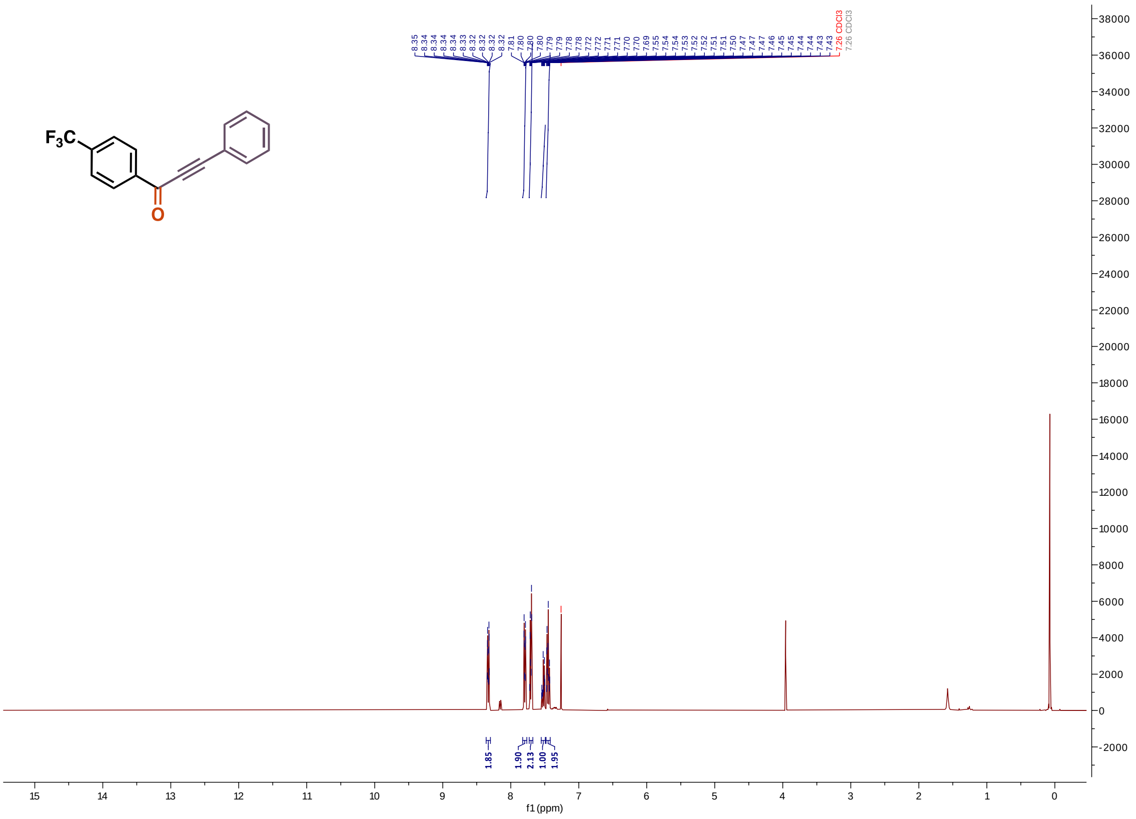

Figure S47. ^{1}H-NMR of **4c**.

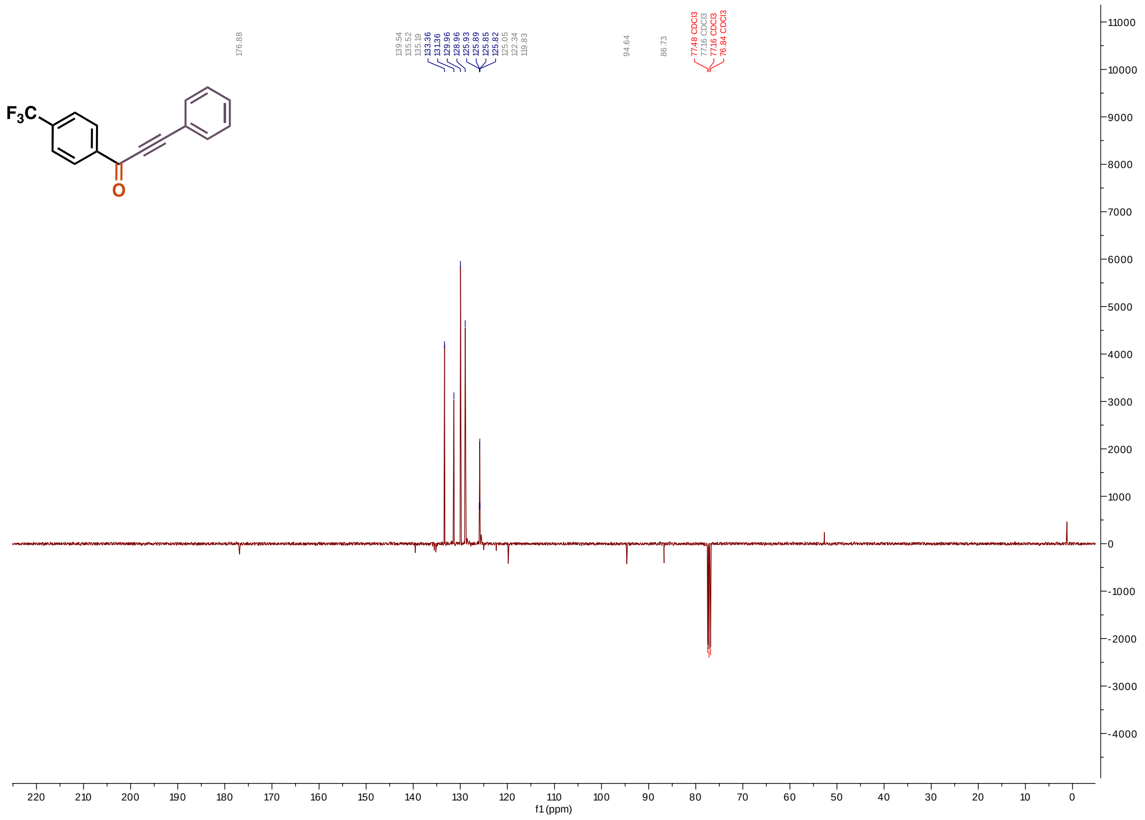

Figure S48. ^{13}C-NMR (APT) of **4c**.

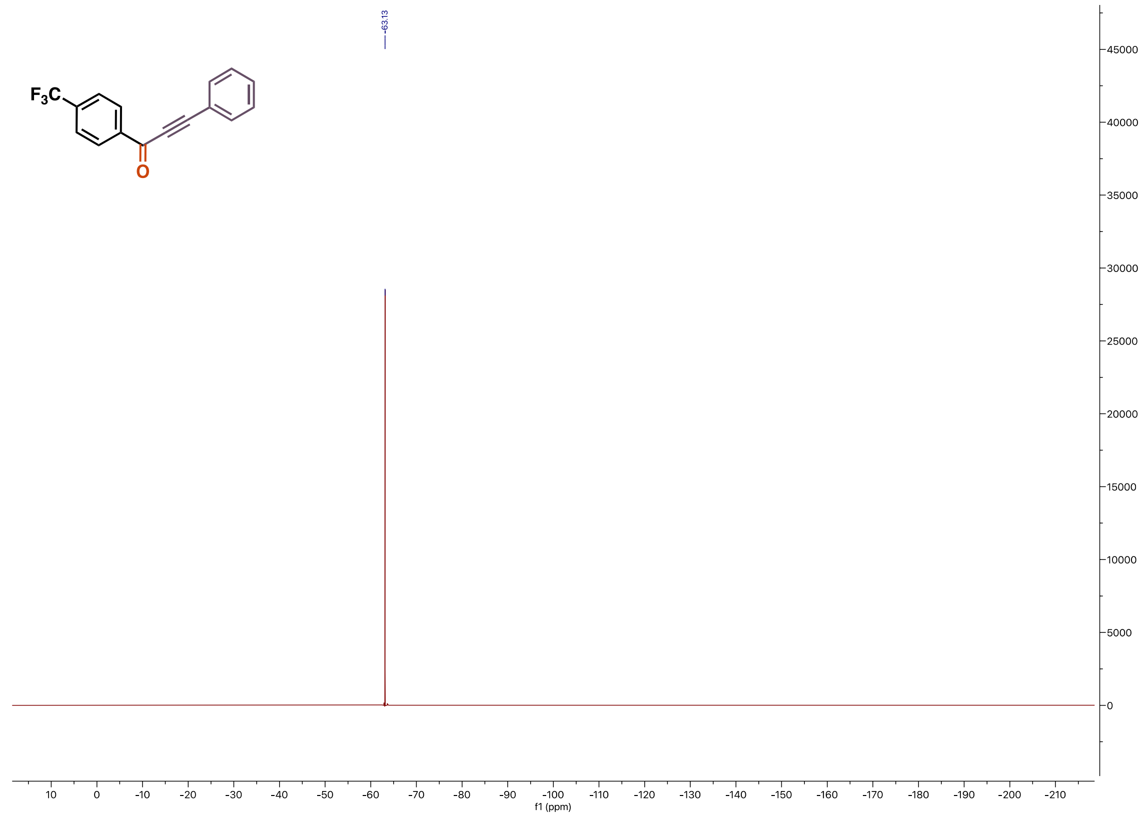

Figure S49. ^{19}F-NMR of **4c**.

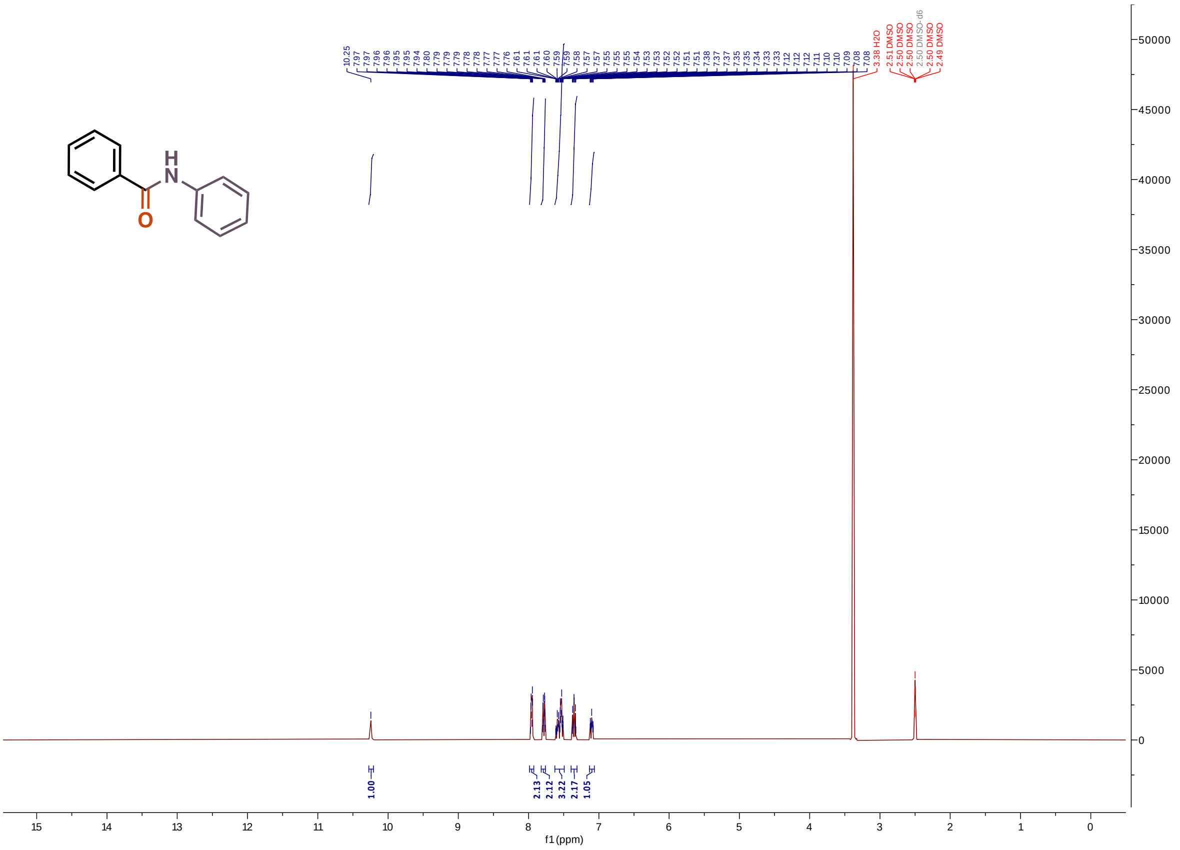

Figure S50. ^{1}H-NMR of **5a**.

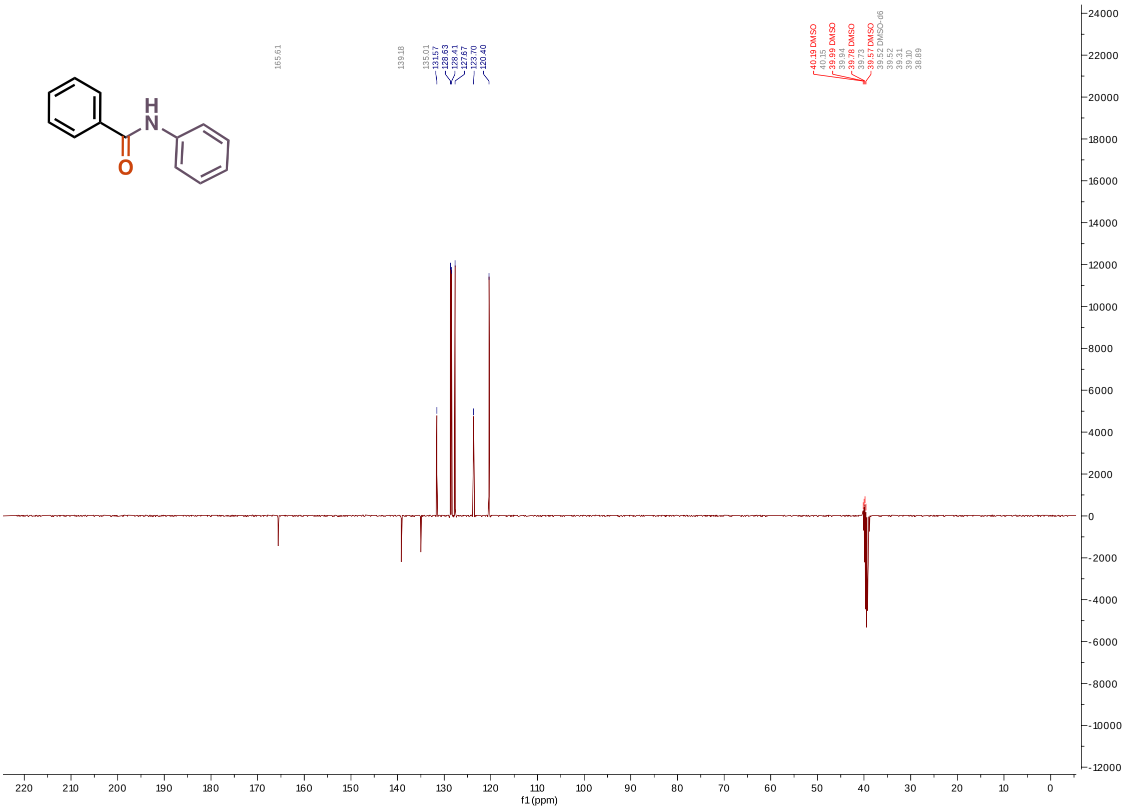

Figure S51. ^{13}C-NMR (APT) of **5a**.

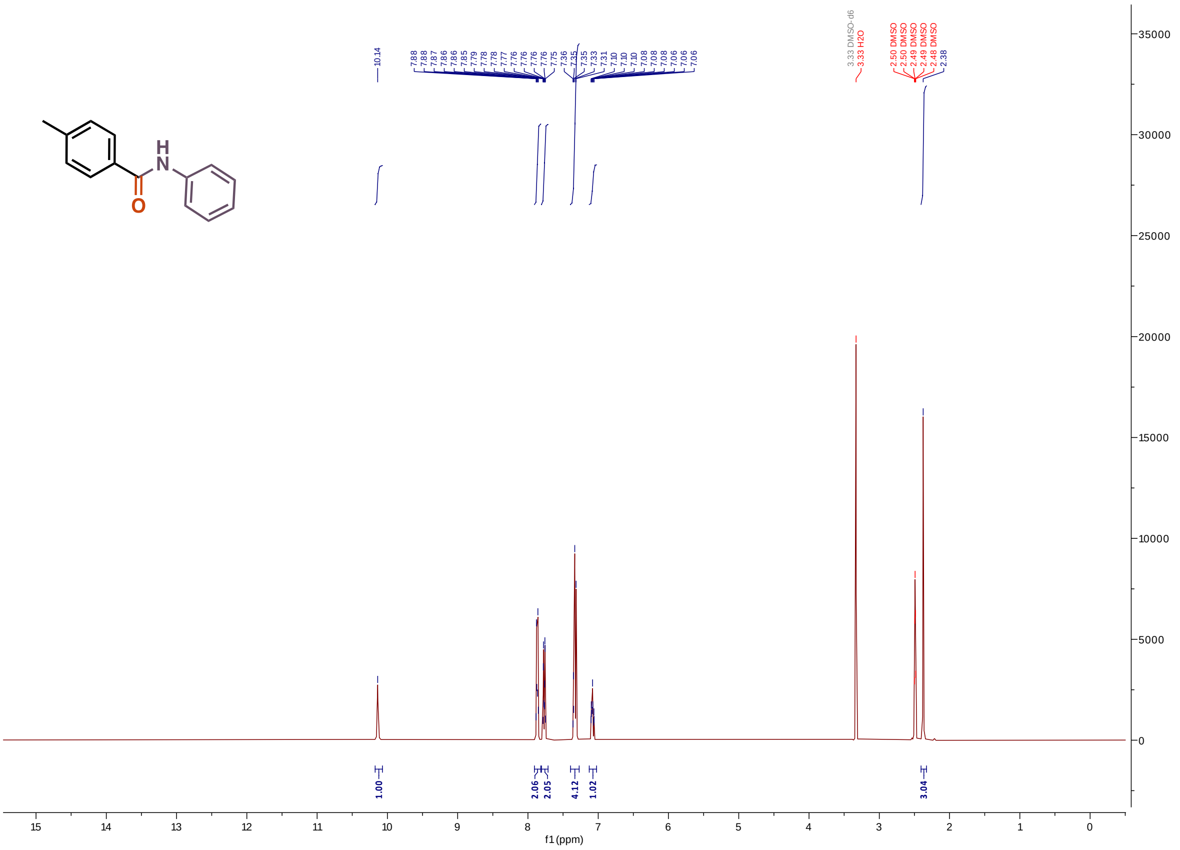

Figure S52. ^{1}H-NMR of **5b**.

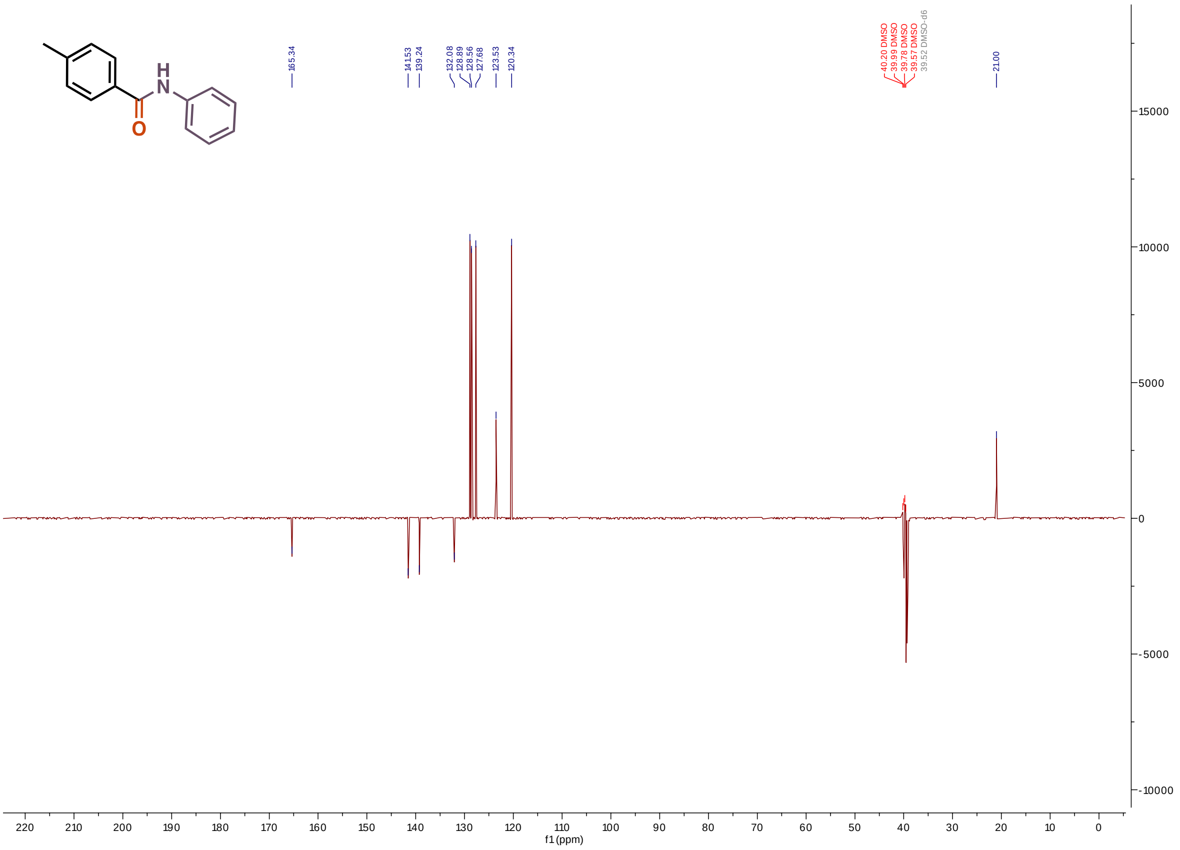

Figure S53. ^{13}C-NMR of **5b**.

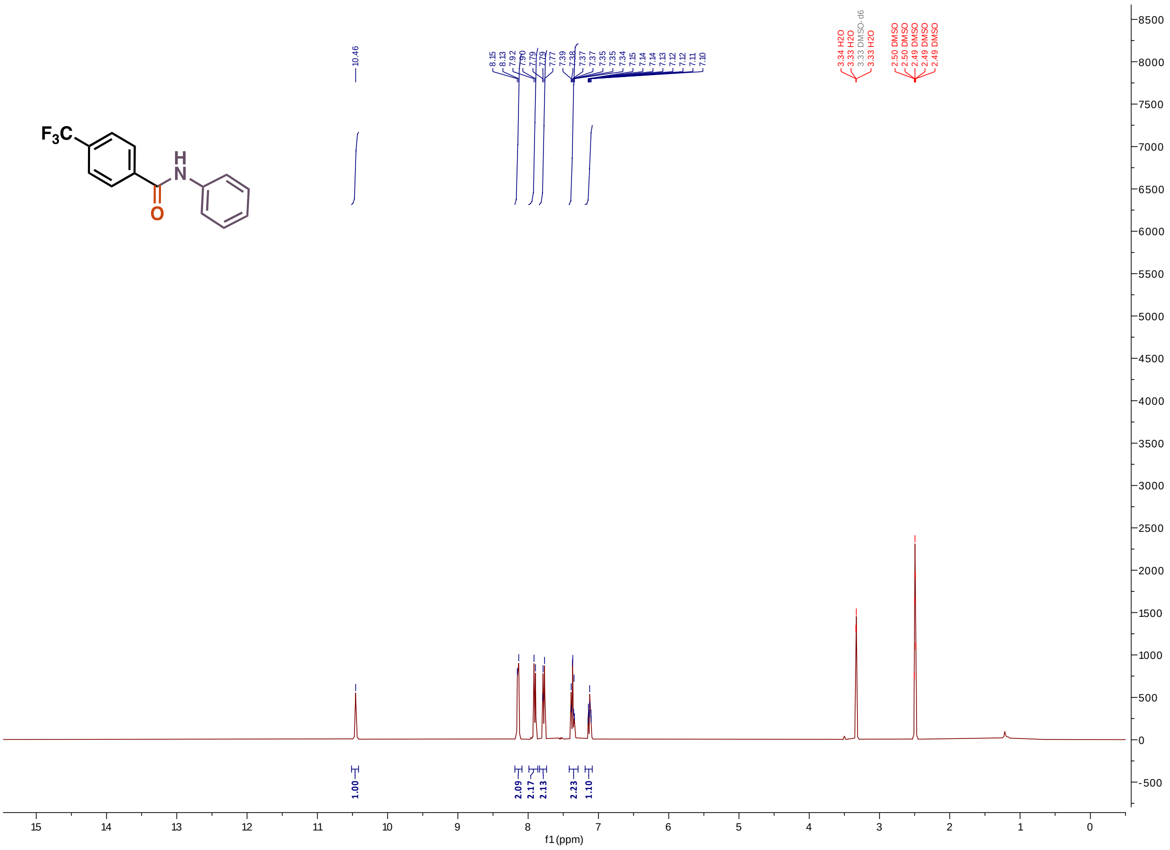

Figure S54. ^{1}H-NMR of **5c**.

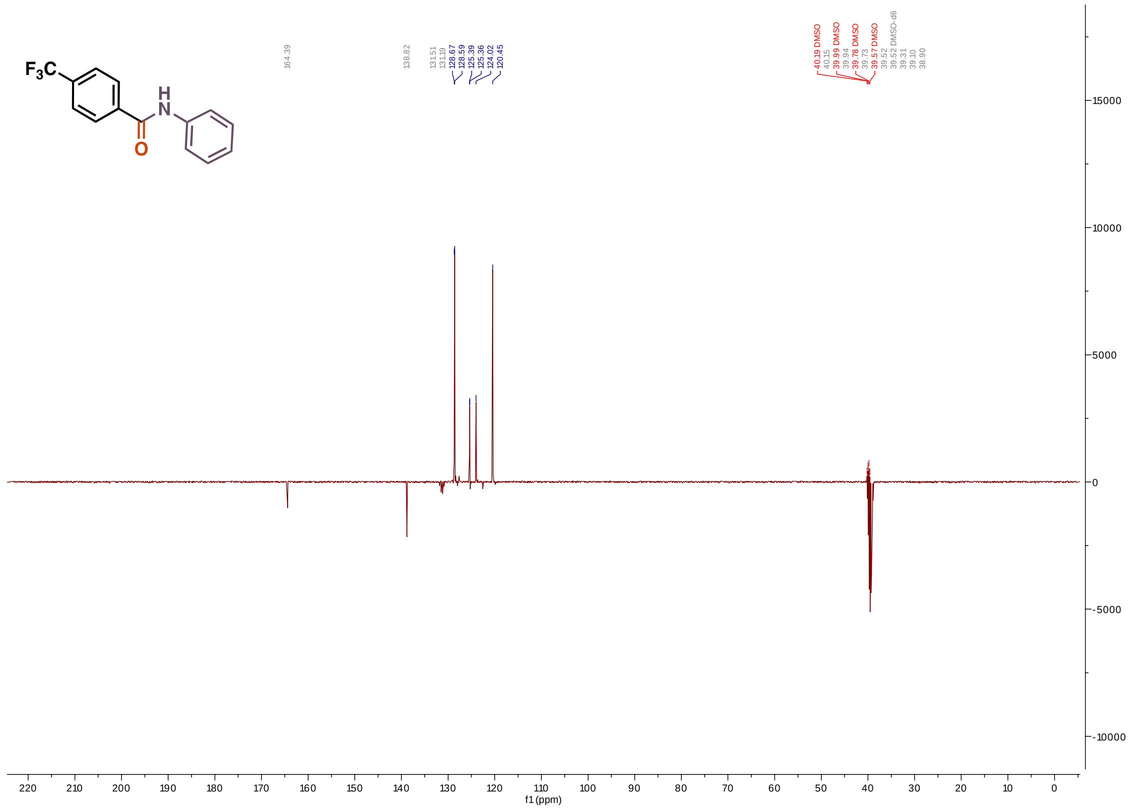

Figure S55. ^{13}C-NMR (APT) of **5c**.

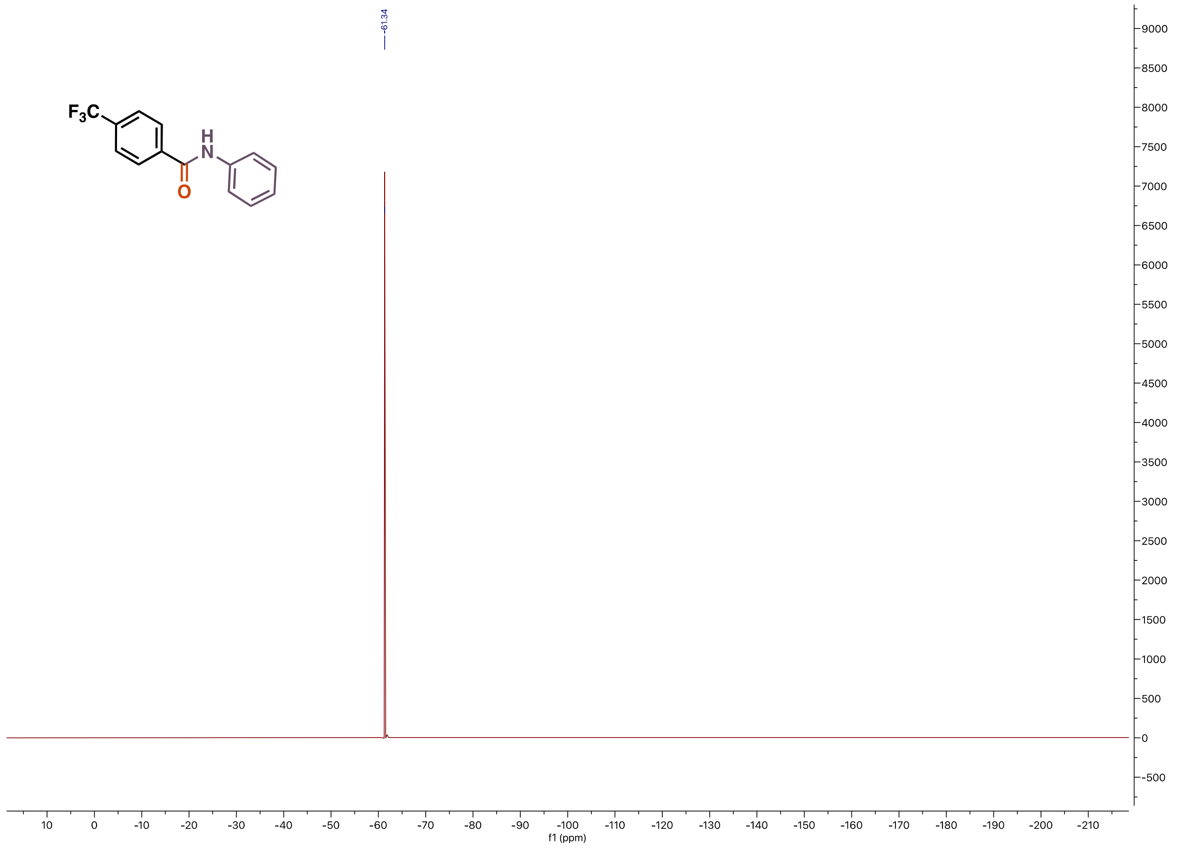

Figure S56. ^{19}F-NMR of **5c**.

20. References

[1] A. Nenning, M. Holzmann, J. Fleig, A. K. Opitz, *Mater. Adv.* **2021**, *2*, 5422-5431.

[2] a) A. Nenning, A. K. Opitz, C. Rameshan, R. Rameshan, R. Blume, M. Hävecker, A. Knop-Gericke, G. Rupprechter, B. Klötzer, J. Fleig, *J. Phys. Chem. C.* **2016**, *120*, 1461-1471; b) M. Gerstl, A. Hutterer, J. Fleig, M. Bram, A. K. Opitz, *Solid State Ionics* **2016**, *298*, 1-8; c) M. Gerstl, A. Nenning, R. Iskandar, V. Rojek-Wöckner, M. Bram, H. Hutter, A. K. Opitz, *Materials* **2016**, *9*, 649; d) A. K. Opitz, A. Nenning, C. Rameshan, M. Kubicek, T. Götsch, R. Blume, M. Hävecker, A. Knop-Gericke, G. Rupprechter, B. Klötzer, J. Fleig, *ACS Appl. Mater. Interfaces* **2017**, *9*, 35847-35860; e) A. Nenning, A. Opitz, *J. Phys. Energy* **2020**, *2*, 014002; f) A. K. Opitz, A. Nenning, V. Vonk, S. Volkov, F. Bertram, H. Summerer, S. Schwarz, A. Steiger-Thirsfeld, J. Bernardi, A. Stierle, J. Fleig, *Nat. Commun.* **2020**, *11*, 4801.

[3] R. Rameshan, A. Nenning, J. Raschhofer, L. Lindenthal, T. Ruh, H. Summerer, A. K. Opitz, T. Martin Huber, C. Rameshan, *Crystals* **2020**, *10*, 947.

[4] Z. A. Feng, M. L. Machala, W. C. Chueh, *Phys. Chem. Chem. Phys.* **2015**, *17*, 12273-12281.

[5] S. Ho, G. Bondarenko, D. Rosa, B. Dragisic, A. Orellana, *J. Org. Chem.* **2012**, *77*, 2008-2012.

[6] M. Haumann, K. Dentler, J. Joni, A. Riisager, P. Wasserscheid, *Adv. Synth. Catal.* **2007**, *349*, 425-431.

[7] B. Urbán, M. Papp, D. Srankó, R. Skoda-Földes, *J. Mol. Catal. A Chem.* **2015**, *397*, 150-157.

[8] C. Jiménez-Rodriguez, A. A. Núñez-Magro, T. Seidensticker, G. R. Eastham, M. R. L. Furst, D. J. Cole-Hamilton, *Catal. Sci. Technol.* **2014**, *4*, 2332-2339.

[9] A. Fusano, S. Sumino, S. Nishitani, T. Inouye, K. Morimoto, T. Fukuyama, I. Ryu, *Chem. Eur. J.* **2012**, *18*, 9415-9422.

[10] P. Losch, A.-S. Felten, P. Pale, *Adv. Synth. Catal.* **2015**, *357*, 2931-2938.

[11] V. V. Gaikwad, B. M. Bhanage, *Appl. Organomet. Chem.* **2019**, *33*, e4741.

[12] X. Pu, J. Hu, Y. Zhao, Z. Shi, *ACS Catal.* **2016**, *6*, 6692-6698.

[13] H. Mei, S. Xiao, T. Zhu, Y. Lei, G. Li, *Trans. Met. Chem.* **2014**, *39*, 443-450.

[14] R. S. Reddy, J. N. Rosa, L. F. Veiros, S. Caddick, P. M. P. Gois, *Org. Biomol. Chem.* **2011**, *9*, 3126-3129.

[15] C.-T. Chen, Y. S. Munot, *J. Org. Chem.* **2005**, *70*, 8625-8627.

[16] G. Wang, Q.-Y. Yu, J. Wang, S. Wang, S.-Y. Chen, X.-Q. Yu, *RSC Adv.* **2013**, *3*, 21306-21310.

[17] J. Dussart-Gautheret, J. Deschamp, M. Monteil, O. Gager, T. Legigan, E. Migianu-Griffoni, M. Lecouvey, *J. Org. Chem.* **2020**, *85*, 14559-14569.

[18] G. Li, M. Arisawa, M. Yamaguchi, *Asian J. Org. Chem.* **2013**, *2*, 983-988.

[19] A. S. Levashov, D. S. Buryi, V. V. Konshin, V. V. Dotsenko, N. A. Aksenov, I. V. Aksenova, *Russ. J. Gen. Chem.* **2017**, *87*, 1627-1630.

[20] S. Karabiyikoglu, Y. Kelgokmen, M. Zora, *Tetrahedron* **2015**, *71*, 4324-4333.

[21] C. Bai, S. Jian, X. Yao, Y. Li, *Catal. Sci. Technol.* **2014**, *4*, 3261-3267.

[22] R. S. Mane, T. Sasaki, B. M. Bhanage, *RSC Adv.* **2015**, *5*, 94776-94785.

[23] Q. Hu, L. Wang, C. Wang, Y. Wu, Z. Ding, R. Yuan, *RSC Adv.* **2017**, *7*, 37200-37207.
